# FIONA1 is an RNA *N*^6^-methyladenosine methyltransferase affecting Arabidopsis photomorphogenesis and flowering

**DOI:** 10.1186/s13059-022-02612-2

**Published:** 2022-01-31

**Authors:** Chunling Wang, Junbo Yang, Peizhe Song, Wei Zhang, Qiang Lu, Qiong Yu, Guifang Jia

**Affiliations:** 1 Synthetic and Functional Biomolecules Center, Beijing National Laboratory for Molecular Sciences, Key Laboratory of Bioorganic Chemistry and Molecular Engineering of Ministry of Education, College of Chemistry and Molecular Engineering, Peking University, Beijing, 100871 China; 2 Peking-Tsinghua Center for Life Sciences, Beijing, 100871 China

**Keywords:** m^6^A, RNA modification, Methyltransferase, mRNA stability, Photomorphogenesis, Early flowering, Arabidopsis

## Abstract

**Background:**

*N*^6^-methyladenosine (m^6^A) mRNA modification is essential for mammalian and plant viability. The U6 m^6^A methyltransferases in other species regulate S-adenosylmethionine (SAM) homeostasis through installing m^6^A in pre-mRNAs of SAM synthetases. However, U6 m^6^A methyltransferase has not been characterized in Arabidopsis and little is known about its role in regulating photomorphogenesis and flowering.

**Results:**

Here we characterize that FIONA1 is an Arabidopsis U6 m^6^A methyltransferase that installs m^6^A in U6 snRNA and a small subset of poly(A)^+^ RNA. Disruption of *FIONA1* leads to phytochrome signaling-dependent hypocotyl elongation and photoperiod-independent early flowering. Distinct from mammalian METTL16 and worm METT-10, FIONA1 neither installs m^6^A in the mRNAs of Arabidopsis SAM synthetases nor affects their transcript expression levels under normal or high SAM conditions. We confirm that FIONA1 can methylate plant mRNA m^6^A motifs in vitro and in vivo. We further show that FIONA1 installs m^6^A in several phenotypic related transcripts, thereby affecting downstream mRNA stability and regulating phytochrome signaling and floral transition.

**Conclusion:**

FIONA1 is functional as a U6 m^6^A methyltransferase in Arabidopsis, distinct from mammalian METTL16 and worm METT-10. Our results demonstrate that FIONA1-mediated m^6^A post-transcriptional regulation is an autonomous regulator for flowering and phytochrome signaling-dependent photomorphogenesis.

**Supplementary Information:**

The online version contains supplementary material available at 10.1186/s13059-022-02612-2.

## Background

Light is one of the most important external signals affecting plant physiology and developmental processes. Upon exposure to light, the critical plant developmental processes, like floral transition and photomorphogenesis, are allowed to proceed. During photomorphogenesis, light inhibits hypocotyl growth, promotes cotyledon opening, and activates the expression of light-regulated genes [1, 2]. Plants have evolved an array of photoreceptors: red and far-red light-absorbing phytochromes (phyA and phyB), blue/ultraviolet (UV)-A light-absorbing cryptochromes (CRY1 and CRY2), and UV-B sensing photoreceptor (UVR8), which transduce light signals to modulate the transcriptome and to trigger photomorphogenic growth and development [3–5]. However, little is known about whether RNA modifications regulate photomorphogenesis.

The most abundant internal mRNA modification *N*^6^-methyladenosine (m^6^A) is reversible and vital in many biological processes through post-transcriptional regulation of mRNA processing and metabolism [6–17]. In both mammals and plants, the m^6^A modification is written by methyltransferase, erased by demethylase, and read by m^6^A-binding proteins [18–21]. Similar to mammals, several Arabidopsis YTH family proteins (ECT2-4 and CPSF30-L) have been characterized as m^6^A binding protein that regulate trichome morphology, leaf growth, nitrate signaling, flowering, and abscisic acid (ABA) response [22–27]; two Arabidopsis (ALKBH9B [28] and ALKBH10B [29]) and one tomato AlkB family proteins (SIALKBH2) [30] have been identified as m^6^A demethylases that regulate viral response, flowering time, and fruit ripening, respectively. Mammals contain two types of mRNA m^6^A methyltransferase: multi-protein complex and methyltransferase like 16 (METTL16) [31, 32]. Multiple subunits of m^6^A methyltransferase complex (comprising METTL3, METTL14, and WTAP three key subunits and others) have been identified in both mammal and plants and are responsible for majority mRNA m^6^A installation [31, 33–39]. Knockout key subunit of m^6^A methyltransferase complex displays embryo lethal in both mammal and plants [36, 40]. Plant WTAP homolog (Arabidopsis FIP37 and rice OsFIP) affects Arabidopsis shoot stem cell fate and early degeneration of rice microspores [38, 41].

Mammalian METTL16 and worm (*Caenorhabditis elegans*) METT-10 were found to install m^6^A on the “UACm^6^AGAGAA” motif of U6 snRNA [32, 42]. The U6 snRNA is an essential component of the spliceosome, contributing to splicing of nuclear pre-mRNAs and serving as ribozyme catalysts of two consecutive transesterifications to ligate two exons concomitant with removal of an intron [43]. They both also methylate pre-mRNAs of SAM synthetases to affect SAM homeostasis with distinct molecular features. Mammalian METTL16 installs m^6^A on the UACm^6^AGAGAA motifs at 3′ UTR of SAM synthetases *MAT2A* pre-mRNA, which leads to intron retention/decay of the RNA and affects downstream SAM homeostasis [32, 44]. The regulation function of METTL16 in SAM homeostasis is essential for mouse early embryonic development [45]. Although depletion of human *METTL16* affects plenty of m^6^A sites in mRNAs [32], recent finding revealed that human METTL16 only methylates U6 m^6^A motif UACm^6^AGAGAA in mRNAs and the increased m^6^A methylation sites lacking U6 m^6^A motif in *METTL16* deficiency were mediated by the reduced intercellular SAM [46]. *Caenorhabditis elegans* (*C. elegans*) METT-10 methylates a variant motif UACm^6^AGAAAC on the 3′ splice site (AG) of SAM synthetase (SAMS) pre-mRNA. The m^6^A on the 3′ splice site (AG) hinders the binding of splicing factor U2AF for splicing; therefore, the unspliced SAMS pre-mRNA is subjected to nuclear degradation [42]. Worms use METT-10’s function in SAM homeostasis to respond to change in worm diet [42]. The UACm^6^AGAGAA and UACm^6^AGAAAC motifs are only conserved in vertebrate and invertebrate SAM synthetase transcripts, respectively [32, 42]. Although sequence alignment suggests that Arabidopsis FIONA1 protein is the homolog of human METTL16 [32], it has not been validated and characterized. It is unknown that whether plant METTL16 would install m^6^A on SAM synthetase to affect SAM homeostasis and whether plant METTL16 relies on the U6 m^6^A motif or a variant U6 m^6^A motif to install m^6^A in poly (A)^+^ RNA.

FIONA1 was identified as a genetic regulator of period length in Arabidopsis circadian clock. The *FIONA1* mutant (*fio1-1*) leads to early flowering and longer hypocotyl in a photoperiod-dependent manner, and lengthens the free-running circadian period of leaf movement [47]. Although FIONA1 was reported to affect the period length of four central oscillator genes’ transcript expression (*CCA1*, *LHY*, *TOC1*, and *LUX*) and the mRNA expression of key flowering regulatory genes *CONSTANS* (*CO*) and *FLOWERING LUCUS T* (*FT*) [47], the molecular mechanism has not been fully elucidated. Little is known about whether and how the methylation activity of FIONA1 regulates the phenotypes in the *fiona1* mutant.

Here we characterized FIONA1 is an Arabidopsis U6 m^6^A methyltransferase that installs m^6^A in U6 snRNA and a small subset of poly(A)^+^ RNA. We asked whether the methylation activity of FIONA1 is required for the reported phenotypes of photoperiod-dependent hypocotyl growth and flowering. Our two homozygous mutant lines *fiona1-1* and *fiona1-2* generated by CRISPR-cas9 displayed hypocotyl cell elongation selectively under continuous red (Rc) and far-red light (FRc) conditions and photoperiod-independent early flowering. Complementation experiments in *fiona1-1* mutant show that the methylation activity of FIONA1 is required for phytochrome signaling-dependent photomorphogenesis and photoperiod-independent flowering time control. We found that the function and methylation activity features of FIONA1 are distinct from mammalian METTL16 and worm METT-10. FIONA1 can methylate plant m^6^A motifs in vitro and in vivo but does not install m^6^A on SAM synthetase transcripts. Then, we asked how the methylation activity of FIONA1 regulates the observed phenotypes. Our results demonstrate that FIONA1-mediated m^6^A post-transcriptional regulation is an autonomous regulator for floral transition and phytochrome signaling-dependent photomorphogenesis.

## Results

### FIONA1 installs m^6^A modification in U6 snRNA and a small subset of poly(A)^+^ RNA in Arabidopsis

To characterize Arabidopsis U6 m^6^A methyltransferase, we processed phylogenetic analysis and sequence alignments and found a single, putative homolog of human METTL16 in Arabidopsis, FIONA1 (*AT2G21070*) (Additional file 1: Fig. S1), which prompted us to test whether FIONA1 deposits m^6^A modification in plants. We generated *fiona1* mutants through CRISPR/Cas9 genome editing system that carried two guiding RNA targeting the coding region of *FIONA1* [48] (Additional file 1: Fig. S2a). Two mutant lines *fiona1-1* and *fiona1-2* were confirmed as homozygous InDel mutants by Sanger sequencing (Additional file 1: Fig. S2b).

**Fig. 1:**
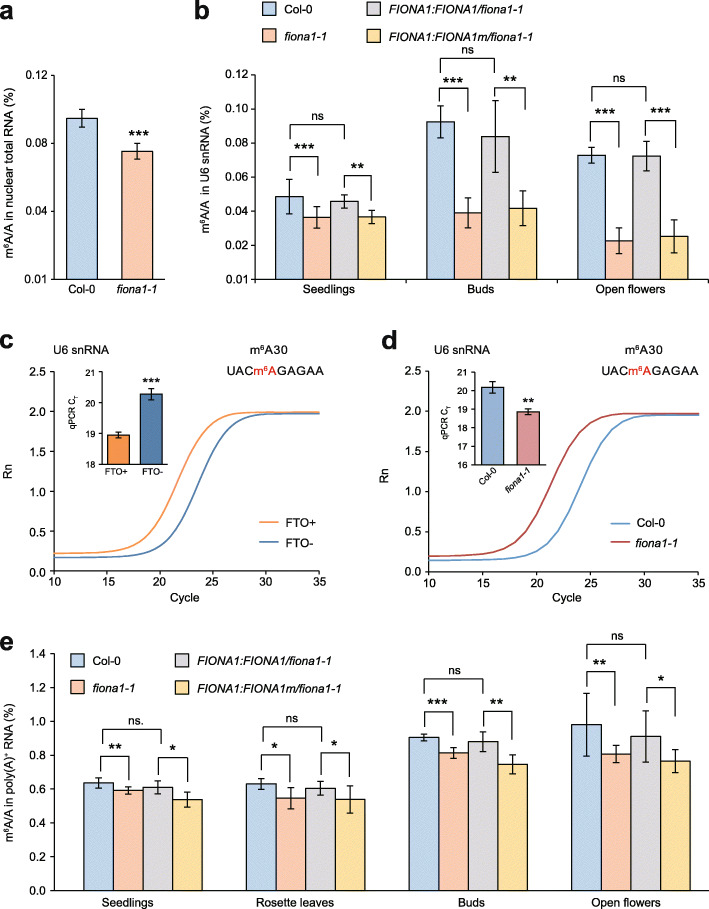
FIONA1 installs m^6^A modification on U6 snRNA and poly(A)^+^ RNA. **a** LC-MS/MS quantification of the m^6^A/A ratio in nuclear total RNA in 12-day-old Col-0 and *fiona1-1* seedlings. **b** LC-MS/MS quantification of the m^6^A/A ratios of U6 snRNA in the indicated genotypic plant tissues. **c-d** Real-time fluorescence amplification curves and bar plot of the threshold cycle (*C*_T_) of qPCR showing SELECT results for detecting m^6^A30 site in U6 snRNA with and without FTO demethylation treatment (**c**) and in Col-0 and *fiona1-1* seedlings (**d**). Rn is the raw fluorescence for the associated well normalized to the fluorescence of the passive reference dye (ROX). **e** LC-MS/MS quantification of the m^6^A/A ratios of poly(A)^+^ RNA in the indicated genotypic plant tissues. Data are means ± SD for 3 biological replicates × 2 technical replicates. * *p* < 0.05, ** *p* < 0.01, *** *p* < 0.001 by *t* test (two-tailed). ns, no significance

To identify the physiological substrates of FIONA1, we firstly separated the nuclear and cytoplasmic fractions of 12-day-old Col-0 and *fiona1-1* seedlings (Additional file 1: Fig. S3a) and isolated their nuclear total RNA for m^6^A quantification. LC-MS/MS results showed that the m^6^A level (the ratio of m^6^A/A) in nuclear total RNA was decreased in *fiona1-1* mutant compared with WT (Fig. 1a). As known that mammalian METTL16 is an m^6^A writer of U6 snRNA [32], we therefore tested whether U6 snRNA is a physiological substrate of FIONA1 in plants. We isolated U6 snRNA from different tissues including 12-day-old seedlings, buds, and flowers (Additional file 1: Fig. S3b) and found that knockout of *FIONA1* significantly reduced m^6^A level in U6 snRNA isolated from these tissues (Fig. 1b). Note that the crystal structure of human METTL16 identified NPPF (residues 184–187) as the catalytic motif for binding substrate adenine base [49] and further biochemical enzymatic assays confirmed METTL16 variants with P185A/P186A or F187G mutations abolish the methyltransferase activity [32, 45]. Based on the sequence alignments among multiple species (Additional file 1: Fig. S1b), we designed an inactive FIONA1 P237A/F239G with mutations of putative catalytic ligand NPPF and named as FIONA1m. To further confirm that U6 snRNA is the physiological substrate of FIONA1, we generated two transgenic complementation lines (*FIONA1:FIONA1/fiona1-1* and *FIONA1:FIONA1m/fiona1-1*) by respectively expressing wild-type FIONA1 and the catalytically inactive mutant FIONA1m in *fiona1-1* mutant plants with the native *FIONA1* promoter (Additional file 1: Fig. S4). The LC-MS/MS results showed that expression of wild-type FIONA1, but not the catalytically inactive mutant FIONA1m in *fiona1-1* mutant, can recover the m^6^A levels in U6 snRNA (Fig. 1b). These results confirmed that U6 snRNA is FIONA1’s substrate and the NPPF ligands in FIONA1 indeed are the catalytic binding motif for adenine base.

The m^6^A modification was reported to locate at A43 position of human U6 snRNA with a hairpin structured conserve sequence UACm^6^AGAGAA [50], but the m^6^A position in plant U6 has not been mapped. We next conducted our developed SELECT method to map m^6^A position in Arabidopsis U6 snRNA. SELECT is an elongation- and ligation-based qPCR quantification method to detect m^6^A locus and fraction in single mRNA/lncRNA at single-base resolution [51]. We run SELECT in two parallel total RNA samples with or without m^6^A demethylase FTO treatments where more than 95% of m^6^A modifications in total RNA were removed by FTO (Additional file 1: Fig. S5a). The FTO-assisted SELECT results showed that m^6^A located at A30 position in Arabidopsis U6 snRNA with the same consensus sequence as human U6 (Fig. 1c; Additional file 1: Fig. S5b). We further performed SELECT in total RNA isolated from 12-day-old Col-0 and *fiona1-1* seedlings and confirmed that FIONA1 is an Arabidopsis U6 m^6^A methyltransferase that is responsible for the m^6^A installation at UACm^6^AGAGAA motif of U6 snRNA (Fig. 1d; Additional file 1: Fig. S5c).

We subsequently assessed the in vivo m^6^A methylation activity of FIONA1 on poly(A)^+^ RNA. Various tissues including seedlings, rosette leaves, buds, and flowers were collected, and isolated poly(A)^+^ RNA were analyzed with LC-MS/MS. In all these tissues, the *fiona1-1* mutant plants had a significantly reduced extent (around 10~15%) of m^6^A modification in poly(A)^+^ RNA compared with Col-0 plants (Fig. 1e). The decreased m^6^A level in *fiona1-1* mutant was restored by expression by wild-type FIONA1, but not by expression of inactive FIONA1m (Fig. 1e). In addition, we measured m^6^A levels in the isolated 28S, 18S, 5S rRNA, and tRNA and revealed that there were no significant differences of m^6^A levels in rRNA and tRNA between Col-0 and *fiona1-1* (Additional file 1: Fig. S6), which excludes rRNA and tRNA from FIONA1’s substrates.

We further monitored mRNA expression levels of known key m^6^A methyltransferase subunits (MTA, MTB, FIP37) and demethylase (ALKBH10B) in seedlings, buds, and flowers by RT-qPCR. We detected no significant differences in mRNA expression of these genes between Col-0 and *fiona1-1* plants (Additional file 1: Fig. S7). The results exclude the effects of MTA-MTB m^6^A methyltransferase complex or m^6^A demethylase on the m^6^A level of poly(A)^+^ RNA in *fiona1-1*. Collectively, we established the finding that FIONA1 is an Arabidopsis U6 m^6^A methyltransferase that installs m^6^A modification on U6 snRNA and a small subset of poly(A)^+^ RNA.

### FIONA1 retains methylation activity towards U6 m^6^A motif and plant mRNA m^6^A motifs in vitro

**Fig. 2:**
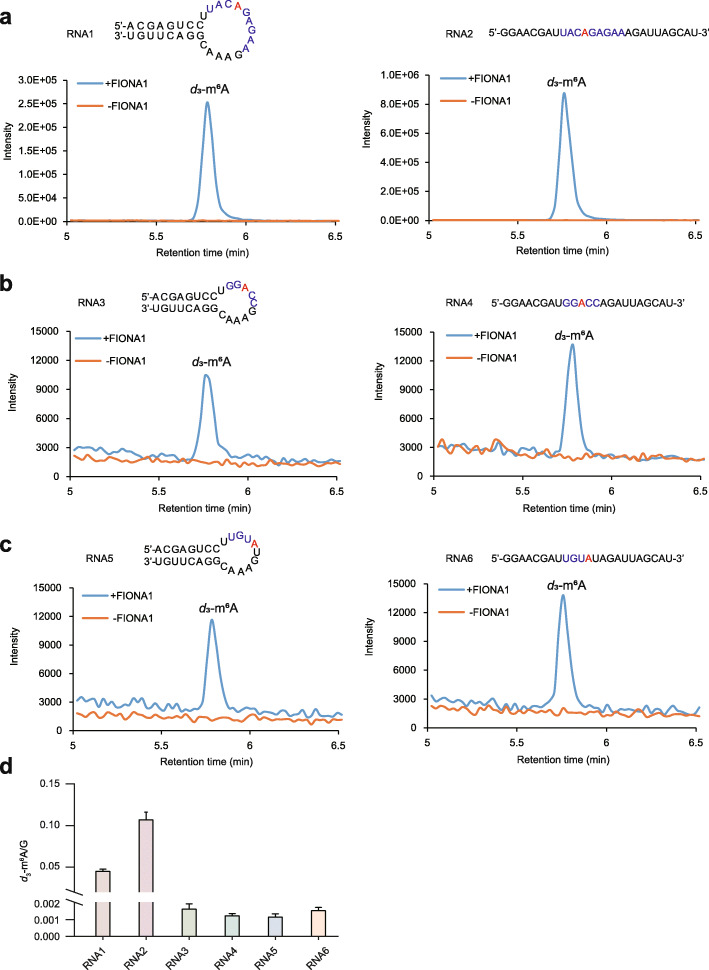
In vitro methylation activity of FIONA1. **a-c** Representative LC-MS/MS chromatograms of *d*_*3*_-m^6^A formed in RNA probes treated with or without FIONA1. The in vitro RNA *N*^6^-adenosine methylation activities of FIONA1 were tested using different RNA probes (numbered 1–6) containing sequence of UACAGAGAA, GGACC and UGUAU in a linear or looped structure in the presence of isotope-labeled cofactor *d*_*3*_-SAM. **d **The peak area ratio of *d*_3_-m^6^A versus one G in the probe (as internal control) was used to evaluate the methylation activities of FIONA1 towards different RNA oligos. Data are means ± SD for 2 biological replicates × 2 technical replicates

Considering that mammalian METTL16 and worm METT-10 respectively install m^6^A modifications only on U6 m^6^A motif UACm^6^AGAGAA or its variant motif UACm^6^AGAAAC within a stem-loop structured RNA [32, 42], we next performed the in vitro *N*^6^-adenosine methylation activity assay to examine whether FIONA1 consistently exhibits the substrate sequence and structure specificity towards U6 m^6^A motif (Additional file 1: Fig. S8). We synthesized six unmethylated RNA oligos with single-strand or stem-loop structure incorporating three different sequences: U6 m^6^A motif UACAGAGAA, and the reported plant mRNA m^6^A motifs GGACC and UGUAU [24, 52]. *S*-(5′-Adenosyl)-L-methionine-*d*_3_ (*d*_3_-SAM) was used as the cofactor in the methylation activity assay and the formation of *d*_3_-m^6^A was used for accurate MS quantification. The peak area ratio of *d*_3_-m^6^A versus one G in the probe (as internal control) was used to evaluate the methylation activities of FIONA1 towards different RNA oligos. The results showed that FIONA1 can transfer methyl group to *N*^6^-adenosine in all six RNA oligos (Fig. 2a–c). Clearly, FIONA1 exhibits higher activity for U6 m^6^A motif over plant mRNA m^6^A motifs GGACC and UGUAU (Fig. 2d). Furthermore, the methylation activity of FIONA1 towards U6 m^6^A motif showed a preference for single-stranded over stem-looped structure (Fig. 2d). The results suggest that unlike mammalian METTL16 and worm METT-10, plant FIONA1 can methylate non-U6 m^6^A motif sequences and its methylation activity does not require RNA secondary structure.

### The m^6^A methylation activity of FIONA1 is required for phytochrome signaling-dependent photomorphogenesis

Consistent with the previous finding that FIONA1 is a nuclear protein [47], our confocal analysis of *FIONA1:FIONA1-GFP/fiona1-1* revealed that FIONA1 was indeed localized in the nuclei of root tip (Additional file 1: Fig. S9). RT-qPCR showed that FIONA1 was widely expressed in diverse Arabidopsis tissues, with especially high expression in floral organs including buds and flowers (Additional file 1: Fig. S10).

**Fig. 3:**
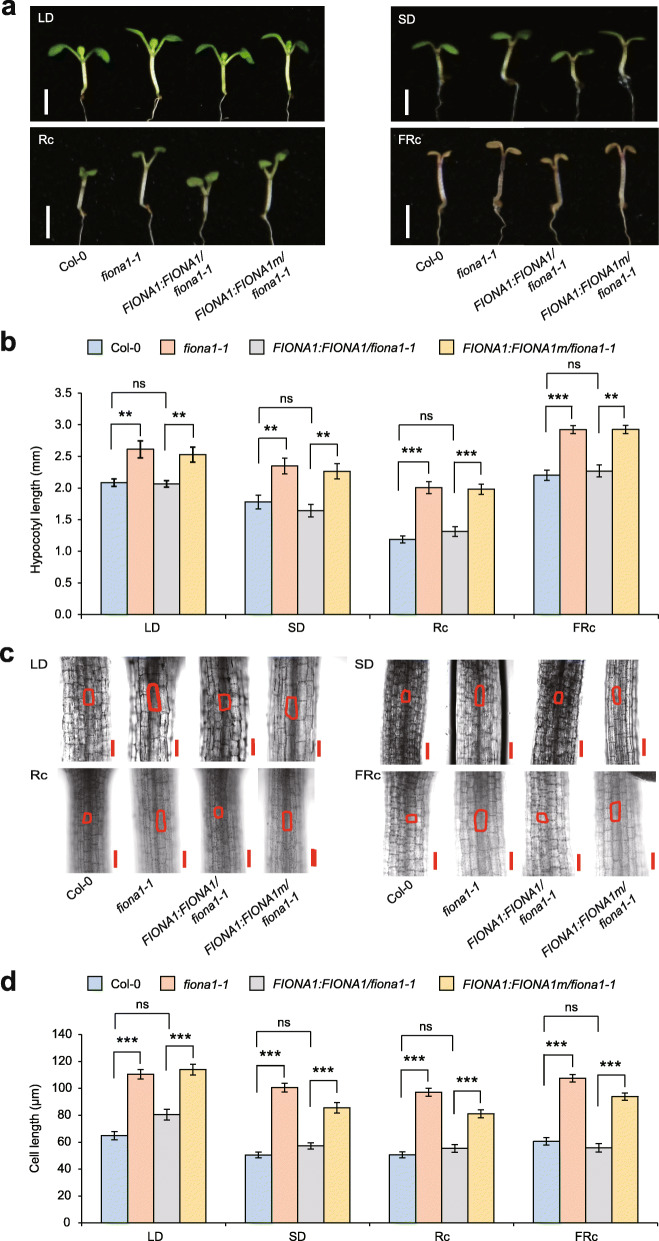
The methylation activity of FIONA1 positively regulates the sensitivity of red light and far-red light and cell elongation. **a** Representative phenotype of Col-0, *fiona1-1*, *FIONA1:FIONA1/fiona1-1*, and *FIONA1:FIONA1m/fiona1-1* seedlings grown under LD, SD, Rc (30 μmol m^−2^ s^−1^), and FRc (11 μmol m^−2^ s^−1^) for 7 days. Bar = 2 mm. **b** Hypocotyl lengths of the seedlings shown in (**a**). Data are means ± SE (*n* ≥ 25). **c** Confocal microscope of hypocotyl epidermal cells of Col-0, *fiona1-1*, *FIONA1:FIONA1/fiona1-1*, and *FIONA1:FIONA1m/fiona1-1* seedlings grown in LD, SD, Rc, and FRc for 7 days. The cell sizes were marked with red lines. Bar = 100 μm. **d** The hypocotyl epidermal cell lengths shown in (c). Data are means ± SE (*n* ≥ 20). ** *p* < 0.01, *** *p* < 0.001 by *t* test (two-tailed). ns, no significance

FIONA1 was previously found to affect photoperiodic hypocotyl growth [47]. Our detailed examination of the hypocotyl growth under long-day (LD, 16 h of light/8 h of dark) and short-day (SD, 8 h of light/16 h of dark) conditions revealed that disruption of *FIONA1* indeed leads to hypocotyl elongation under both LD and SD conditions (Fig. 3a); however, the hypocotyl length ratio of *fiona1-1* versus Col-0 (1.25 for LD and 1.32 for SD) did not show a significant difference between these two photoperiodic conditions (Fig. 3b). In contrast with the previous finding [47], our results suggest the phenotype of hypocotyl elongation in *fiona1-1* is not dependent on photoperiod. Although it was reported that FIONA1 does not affect hypocotyl growth under constant light [47], we next investigated the hypocotyl growth in *fiona1-1* under continuous light with different wavelength. The results showed that *fiona1-1* exhibited elongated hypocotyls only under continuous red (Rc) and far-red light (FRc), but not under continuous blue light (Bc), white light (Wc), and dark (Dk) (Fig. 3a, b; Additional file 1: Fig. S11a, b). Consistently, the homozygous *fiona1-2* mutant also led to hypocotyl elongation phenotype selectively under Rc and FRc conditions compared with Col-0 (Additional file 1: Fig. S12). In higher plants, phytochrome A (phyA) and phytochrome B (phyB) are the most abundant members of the photoreceptors family and their deficiencies are most evident under continuous Rc and FRc, respectively. Although phyA and phyB activities occur under different light conditions, the end-point responses (e.g., hypocotyl growth, cotyledon unfolding, flowering) controlled by which are largely the same [53]. Therefore, the selective hyposensitivity to Rc and FRc in *FIONA1*-defective mutants suggests FIONA1 is a positive regulator of photomorphosis, especially in phyA and phyB signaling transduction in Arabidopsis.

We subsequently investigated whether hypocotyl photomorphogenesis is dependent on the methylation activity of FIONA1. The rescue experiments showed that the selective hyposensitivity to Rc and FRc in *fiona1-1* can be restored by expression of wild-type FIONA1, but not inactive FIONA1m (Fig. 3a, b). Thus, the methylation activity of FIONA1 regulates phytochrome signaling-dependent hypocotyl photomorphogenesis. Furthermore, we sought clue about altered hypocotyl at the histological level and found that the epidermal cells in the upper part of the hypocotyl (the part near the cotyledon) were elongated in *fiona1-1* under LD, SD, Rc, and FRc conditions (Fig. 3c, d; Additional file 1: Fig.S11c, d), suggesting the phenotype of hypocotyl elongation in *fiona1-1* is due to the cell elongation. Expectedly, the elongation of hypocotyl epidermal cells is dependent on the m^6^A methylation activity of FIONA1 (Fig. 3c, d; Additional file 1: Fig. S11c, d).

### The m^6^A methylation activity of FIONA1 is required for floral transition

**Fig. 4:**
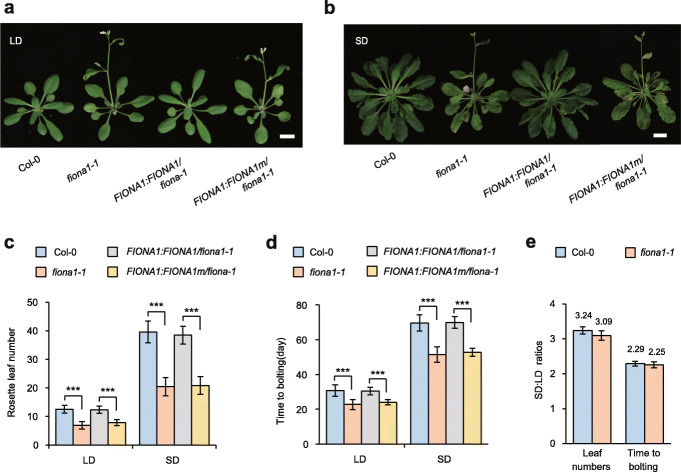
The methylation activity of FIONA1 is required for floral transition. **a-b** Phenotypes of the floral transition in Col-0, *fiona1-1*, *FIONA1:FIONA1/fiona1-1*, and *FIONA1:FIONA1m/fiona1-1* plants grown under LD (**a**, 16 L/8D) and SD (**b**, 8 L/16D) conditions. Bar = 1 cm. **c-d** Statistical analysis of total rosette leaf number (**c**) and days to flowering (**d**) at the bolting stage in Col-0, *fiona1-1*, *FIONA1:FIONA1/fiona1-1*, and *FIONA1:FIONA1m/fiona1-1* plants under LD (16 L/8D) and SD (8 L/16D) conditions. Data are means ± SD (*n* ≥ 20) *** *p* < 0.001 by *t* test (two-tailed). **e** The SD: LD ratios of rosette leaf number and time to bolting at the bolting stage in Col-0 and *fiona1-1* plants

FIONA1 was reported to affect daylength-dependent flowering [47] but its underlying biological function is unknown. We next asked whether the phenotype caused in the *fiona1* mutant was dependent on the m^6^A catalytically activity of FIONA1. Our two CRISPR-cas9 generated homozygous mutant lines *fiona1-1* and *fiona1-2* both display early flowering phenotypes compared with Col-0 under LD and SD conditions (Fig. 4a–d; Additional file 1: Fig.S13), consistent with the previous finding [47]. However, we did not observe a significant difference in photoperiodic flowering response under LD and SD conditions in our *fiona1* homozygous mutant line (Fig. 4e). Expression of wild-type *FIONA1* in *fiona1-1* (*FIONA1:FIONA1 /fiona1-1*) can completely recover the early flowering phenotype in *fiona1-1* under LD and SD conditions; however, complementation of the inactive mutant FIONA1m with P237A/F239G mutations (*FIONA1:FIONA1m/fiona1-1*) did not complement the phenotype (Fig. 4a–d). These results demonstrated that the observed flowering phenotype is dependent on the m^6^A methylation activity of FIONA1.

### Disruption of *FIONA1* leads to transcriptomic m^6^A hypomethylation

**Fig. 5:**
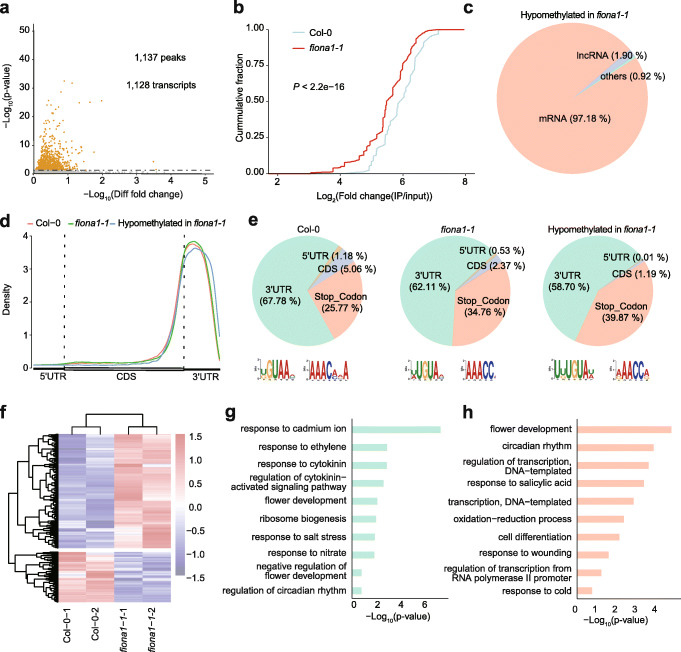
Disruption of *FIONA1* leads to transcriptome m^6^A hypomethylation. **a** Volcano plot of the hypomethylated m^6^A peaks identified in *fiona1-1* across three biological replicates. **b** Cumulative fraction of log_2_ (m^6^A enrichment fold change) for the hypomethylated m^6^A peaks in *fiona1-1* and Col-0*.* Pie chart presenting RNA types of the confident hypomethylated m^6^A peaks identified in *fiona1-1.* **d** Metagene profile presenting the distributions of m^6^A peaks identified in Col-0 and *fiona1-1* and the hypomethylated m^6^A peaks in *fiona1-1* across the indicated mRNA segments. **e** Pie chart presenting the fractions of m^6^A peaks identified in Col-0 and *fiona1-1* and the hypomethylated m^6^A peaks in *fiona1-1* among non-overlapping transcript segments and the MEME-identified motifs for those m^6^A peaks. **f** Heatmap showing differentially expressed genes in two biological replicated Col-0 and *fiona1-1* seedlings. **g** GO analysis of the hypomethylated m^6^A containing genes in *fiona1-1.* **h** GO analysis of the differentially expressed genes in *fiona1-1* compared with Col-0

To investigate the global effects of FIONA1-mediated m^6^A poly(A)^+^ RNA modification landscape, we performed m^6^A-seq that combines anti-m^6^A antibody immunoprecipitation (m^6^A-IP) and high-throughput sequencing [54–56] in 12-day-old Col-0 and *fiona1-1* seedlings (three biological replicates for each genotype). As summarized in Additional file 3: Table S1, around 47–166 million reads per sample were mapped to Arabidopsis TAIR 10 genome. We used MACS2 algorithm with an estimated false discovery rate (FDR) < 0.05 and an enrichment of ≥ 2 to call m^6^A peaks. The m^6^A peaks in both replicates were classified as “confident peaks.” We identified 10,840 m^6^A confident peaks corresponding 12,345 transcripts/genes in Col-0 and 11,238 m^6^A confident peaks corresponding to 12,461 transcripts/genes in *fiona1-1* (Additional file 1: Fig. S14; Additional file 4: Table S2; Additional file 5: Table S3). To seek the potential m^6^A methylation targets of FIONA1, we conducted ExomePeak algorithm (| fold change | > 1 and FDR < 0.05) to calculate the differential m^6^A peaks between Col-0 and *fiona1-1*. We identified 1137 confident hypomethylated m^6^A peaks corresponding 1128 transcripts/genes in *fiona1-1*(Fig. 5a, b; Additional file 6: Table S4), which predominantly belong to mRNA (Fig. 5c). To validate our m^6^A-seq results were reliable and accurate, we randomly selected four m^6^A hypomethylated genes and conducted m^6^A-IP-qPCR of fragmented poly(A)^+^ RNA from Col-0 and *fiona1-1*. These results showed that the m^6^A methylation on these four transcripts were significantly reduced in *fiona1-1* (Additional file 1: Fig. S15), suggesting the identified confident hypomethylated m^6^A peaks in *fiona1-1* could be the methylation targeted sites of FIONA1.

We next evaluated the substrates preference of FIONA1. Three groups of m^6^A peaks—the identified confident hypomethylated m^6^A peaks in *fiona1-1*, the confident m^6^A peaks in Col-0, and the confident m^6^A peaks in *fiona1-1*—were used to investigate the m^6^A distribution across transcripts and the m^6^A motif. The metagene profiles revealed that the confident hypomethylated m^6^A peaks in *fiona1-1* highly located around stop codon and within 3′ UTR, which was the same as the whole m^6^A distribution pattern of Col-0 and *fiona1-1* (Fig. 5d). We divided transcripts into five non-overlapping segments: 5′ UTRs, Start codon (100-nucleotide window centered on the start codon), coding sequences (CDS), Stop codon (100-nucleotide window centered on the stop codon), and 3′ UTRs. The m^6^A peaks from the three groups were mainly located in 3′ UTR and stop codon region, but the fraction of the hypomethylated m^6^A peaks in stop codon portion is slightly higher compared to Col-0 (Fig. 5e). We clustered these three groups of m^6^A peaks in MEME software package to search motifs. We did not observe U6 m^6^A motif enriched in the hypomethylated m^6^A peaks. Instead, we found that the enriched motifs (UKUGUAW (K=U or G; W=U or A) and RAACCR (R = A or G)) in the confident hypomethylated m^6^A peaks in *fiona1-1* were similar as those identified in all m^6^A peaks in either Col-0 or *fiona1-1* (Fig. 5e; Additional file 1: Fig. S16). Note that UGUA is a known plant-unique m^6^A motif [24] and RAACC resembles m^6^A consensus motif RRACH [52, 57]. Thus, FIONA1 exhibits no obvious preference in m^6^A position and motif in comparison to whole m^6^A peaks in Col-0 and *fiona1-1*.

We subsequently analyzed our RNA-sequencing performed in 12-day-old Col-0 and *fiona1-1* seedlings and identified 267 downregulated genes and 605 upregulated genes in *fiona1-1* compared with Col-0 (threshold criteria with | log2(fold change) | ≥ 0.48 and *P* value < 0.05) (Fig. 5f; Additional file 7: Table S5). To gain functional insights into the role of FIONA1, we performed Gene Ontology (GO) analysis using the DAVID tools on 1128 m^6^A hypomethylated genes and 872 differential expressed genes in *fiona1-1*, respectively. GO analysis revealed the m^6^A hypomethylated genes were positively enriched in response to cadmium ion, ethylene, and cytokinin, and regulation of cytokinin-activated signaling pathway, flower development, and circadian rhythm (Fig. 5g). GO analysis of the differential expressed genes showed that *FIONA1* deficiency affects several developmental pathways, including flower development, circadian rhythm, response to salicylic acid, and cell differentiation (Fig. 5h). These gene functions showed clear correlation with the observed phenotypes in *fiona1* mutant.

mRNA modification m^6^A regulates alternative splicing in mammals and mammalian METTL16 affects the splicing of *MAT2A* mRNA for intron retention [12, 13, 32, 58]. It was predicated that removal of m^6^A modification from U6 snRNA also could affect splicing [59, 60]. Thus, we investigated whether FIONA1 affects alternative splicing. We analyzed the changes of alternative splicing between Col-0 and *fiona1-1* using rMATS tools [39, 61] and found only 43 alternative splicing events (FDR < 0.05) occurred in *fiona1-1* compared with Col-0 (Additional file 8: Table S6). Among them, 4 genes contain confident m^6^A hypomethylation peaks. Therefore, these results showed that disruption of *FIONA1* does not affect global alternative splicing.

### Differences in methylation sites between FIONA1 and MTA / MTB/FIP37 complex

The MTA/MTB/FIP37 methyltransferase complex is responsible for the majority of mRNA methylation in plants. Disruption of the key subunit of the methyltransferase complex leads to embryonic lethal in Arabidopsis [36, 38]. Conditional expression of *FIP37* driven by *LEC1* promoter in the *fip37* mutant causes more than 80% of m^6^A decrease in poly(A)^+^ RNA [38]. However, *FIONA1* deficiency has good viability and only reduces 10~15% of m^6^A level in poly(A)^+^ RNA, indicating the small subset of m^6^A sites methylated by FIONA1 would be distinct from the targets of the MTA/MTB/FIP37 methyltransferase complex. To validate it, we termed the hypomethylated m^6^A in *fiona1-1* as “FIONA1-dependent m^6^A” and the rest m^6^A peaks in Col-0 excluding FIONA1-dependent m^6^A as “FIONA1-independent m^6^A.” We compared these two groups with FIP37-dependent m^6^A peaks, which were identified from the published m^6^A-seq results in *LEC1:FIP37/fip37-4* and control plants [38]. We found that 84% (3126 out of 3699) of FIP37-dependent m^6^A peaks were overlapped with FIONA1-independent m^6^A but only 10% (379 out of 3699) overlapped with FIONA1-dependent m^6^A (Additional file 1: Fig. S17a). We further calculated the distance between stop codon and m^6^A sites from the three groups. The results showed that both FIP37-dependent m^6^A and FIONA1-independent m^6^A peaks were highly enriched around the stop codon, while FIONA1-dependent m^6^A were enriched at the downstream of stop codon (Additional file 1: Fig. S17b). Collectively, these results suggest that FIONA1 only methylates a small subset of m^6^A sites in poly(A)^+^ RNA and its targeted sites are distinct from the methylation sites of MTA/MTB/FIP37 methyltransferase complex.

### FIONA1 does not methylate SAM synthetase transcripts

As U6 m^6^A methyltransferase, mammalian METTL16 and worm METT-10 install m^6^A in U6 or U6-like m^6^A motifs of SAM synthetase to affect its gene expression and SAM homeostasis under high SAM conditions [32, 42]. Considering that FIONA1 is Arabidopsis U6 methyltransferase, we next investigated whether FIONA1 methylates SAM synthetases. Arabidopsis contains four genes encoding SAM synthetase: *MAT1* (*AT1G02500*), *MAT2* (*AT4G01850*), *MAT3* (*AT2G36880*), and *MAT4* (*AT3G17390*). We found none of them contain U6 m^6^A motif UACm^6^AGAGAA or U6-like m^6^A motif UACm^6^AGAAAC (Additional file 1: Fig. S18). Our m^6^A-seq results showed that the m^6^A modifications were localized on the transcripts of SAM synthetases but were not installed by FIONA1 (Additional file 1: Fig. S19a). RNA-seq and RT-qPCR results revealed that *FIONA1* deficiency did not affect their expression levels compared with WT under normal growth condition (Additional file 1: Fig. S19b, c). Next, we investigated whether FIONA1 would methylate SAM synthases under high SAM conditions; therefore, we grew Arabidopsis seedlings on the agar plates with different concentrations of SAM or L-methionine (L-Met) for 12 days. We measured and confirmed that the SAM concentration inside seedlings was continuously increased under SAM or L-Met treatments and elevated by 2-fold under 100 mg/L SAM or 50 mg/L L-Met treatment compared non-treatments (Additional file 1: Fig. S20a, b). The RT-qPCR results revealed that no significant differences in pre-mRNA and mRNA expression levels of SAM synthetases *MAT1*-*4* in *fiona1-1* compared to WT seedlings grown under high SAM conditions (Additional file 1: Fig. S20c, d). These results suggest that unlike mammalian and worms, Arabidopsis does not use FIONA1’ methylation activity to affect the expression level of SAM synthetase and SAM homeostasis.

### FIONA1 installs m^6^A on non-U6 m^6^A motifs in photomorphogenic- and flowering-related transcripts in vivo

Excluding the methylation function of FIONA1 on SAM synthetases, we subsequently explored the molecular mechanism by which FIONA1-mediated m^6^A methylation regulates phytochrome signaling-dependent photomorphogenesis and floral transition. The GO analysis revealed that the m^6^A hypomethylated genes and the differential expressed genes in *fiona1* mutant were enriched in flower development pathway (Fig. 5g, h). The m^6^A-seq and RNA-seq results showed that m^6^A at 3′ UTR of *CRY2* (*CRYPTOCHROMES 2*) and *FLC* (*FLOWERING LOCUS C*) transcripts were significantly reduced in *fiona1-1* (Additional file 1: Fig. S21; Additional file 6: Table S4) and their mRNA expression levels were also significantly changed (Additional file 7: Table S5). CO (CONSTANS) is a key regulator of the photoperiodic flowering pathway [62]. The RNA-seq showed that *CO* mRNA was significantly upregulated in *fiona1-1*, consistent with previous report [47]. We did not identify m^6^A peak on *CO* transcript in the m^6^A-seq results, which might be the low expression level of *CO* at Zeitgeber time (ZT) 13 of LD condition. Thus, we chose three flowering-related genes *CRY2*, *FLC*, and *CO* for further validation. PIF4 (PHYTOCHROME INTERACTING FACTOR4) directly interacts with light-activated phytochromes and is a positive regulator in cell elongation [63]. The hypocotyl elongation of *pif4* mutants is specifically defective in responsiveness to red light, the double mutant of *PIF4* and its close homolog *PIF5* (*pif4pif5*) show hypersensitivity to far-red light, suggesting that they redundantly control the far-red light responses [64]. In consistent with phenotype of hypocotyl cell elongation selectively under Rc and FRc in *fiona1* mutants, RNA-seq revealed upregulation of *PIF4* expression level in *fiona1-1* (Additional file 7: Table S5)*.* Among three biological replicate m^6^A-seq, we observed the significant m^6^A decreases in two biological replicates of *fiona1-1* samples (Additional file 1: Fig. S21). Therefore, we selected PIF4 for further validation.

**Fig. 6:**
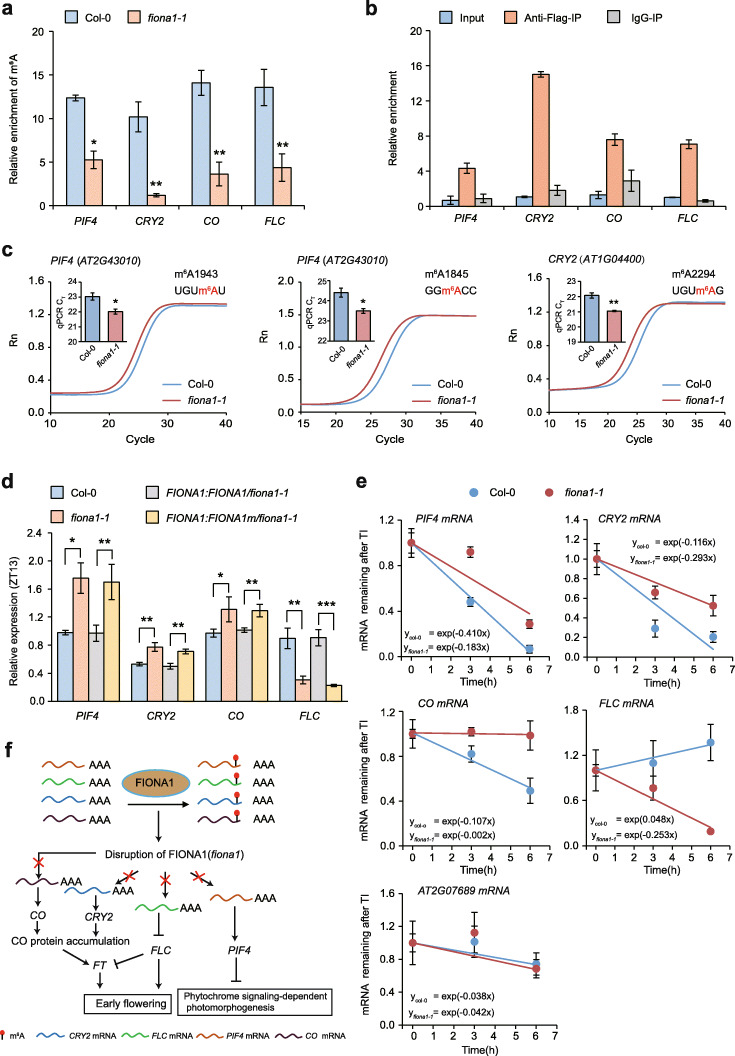
FIONA1-mediated m^6^A installation affects the stability of *PIF4*, *CRY2*, and *FLC* transcripts. **a** m^6^A-IP-qPCR results showing the relative m^6^A levels of *PIF4*, *CRY2*, and *FLC* transcripts in 12-day-old Col-0 and *fiona1-1* seedlings. Data are means ± SD for 3 biological replicates × 3 technical replicates. * *p* < 0.05, ** *p* < 0.01 by *t* test (two-tailed). **b** RIP-qPCR assays in *FIONA1:FIONA1-FLAG*/ *fiona1-1* plants showing that FIONA1 directly binds *PIF4*, *CRY2*, and *FLC* transcripts. Data are means ± SD for 3 biological replicates × 2 technical replicates. **c** Real-time fluorescence amplification curves and bar plot of the threshold cycle (*C*_T_) of qPCR showing SELECT results for detecting the FIONA1-targeted m^6^A sites in *PIF4* and *CRY2* mRNAs in Col-0 and *fiona1-1* seedlings. Rn is the raw fluorescence for the associated well normalized to the fluorescence of the passive reference dye (ROX). Data for bar plot are means ± SD for 3 biological replicates × 2 technical replicates. * *p* < 0.05, ** *p* < 0.01 by *t* test (two-tailed). **d** The relative expression levels of *PIF4*, *CRY2* and *FLC* at ZT13 in the indicated genotypic plants under LD conditions. *TUB2* was used as the internal control gene. Data are means ± SD for 3 biological replicates × 3 technical replicates. * *p* < 0.05, ** *p* < 0.01 by *t* test (two-tailed). **e** The mRNA lifetimes of *PIF4*, *CRY2*, and *FLC* in Col-0 and *fiona1-1*. The *AT2G07689* was used as the negative control. TI: transcription inhibition. Data are represented as means ± SD for 2 biological replicates × 3 technical replicates. **f** Proposed model describing the molecular mechanism through which FIONA1-mediated m^6^A installation regulates Arabidopsis phytochrome signaling-dependent photomorphogenesis and floral transition

To further verify *PIF4*, *CRY2*, *CO*, and *FLC* were directly methylation targets of FIONA1, we performed m^6^A-IP qPCR assays on fragment poly(A)^+^ RNA isolated from 12-day-old Col-0 and *fiona1-1* seedlings at ZT13 of LD condition. We confirmed that the m^6^A levels (or m^6^A enrichment) on these identified m^6^A peaks of *PIF4*, *CRY2*, *CO*, and *FLC* were significantly reduced in *fiona1-1* compared with Col-0 (Fig. 6a). RNA immunoprecipitation (RIP)-qPCR assays using 12-day-old *FIONA1:FIONA1-Flag/fiona1-1* seedlings at ZT13 of LD condition showed that FIONA1 protein directly binds *PIF4*, *CRY2*, *CO*, and *FLC* transcripts (Fig. 6b). Considering FIONA1 is an m^6^A methyltransferase and does not affect the intercellular SAM level, these results revealed that FIONA1 directly installs m^6^A on *PIF4*, *CRY2*, *CO*, and *FLC* transcripts.

There are no U6 m^6^A motifs or variant U6 m^6^A motifs in *PIF4*, *CRY2*, *CO*, and *FLC* transcripts. Considering our finding that FIONA1 can methylate plant mRNA m^6^A motifs GGACC and UGUAU in vitro (Fig. 2), we next chose *PIF4* and *CRY2* to investigate whether FIONA1 can install m^6^A on non-U6 m^6^A motif in vivo. The expression levels of *PIF4* and *CRY2* are highly abundant, which allows us to determine m^6^A sites using SELECT method, a qPCR method for quantitative detection of m^6^A locus and fraction in mRNA at single-base resolution [51]. We firstly performed FTO-assisted SELECT on Arabidopsis total RNA to determine m^6^A positions in the identified FIONA1-targeted m^6^A peaks (Fig. 6a, b). Two m^6^A sites in UGUm^6^A1943U and GGm^6^A1845CC sequences and one m^6^A site in UGUm^6^A2294G sequence were respectively identified within 3′ UTR regions of *PIF4* and *CRY2* (Additional file 1: Fig. S22). Direct SELECT performed in total RNA isolated from Col-0 and *fiona1-1* showed that these three m^6^A sites identified in *PIF4* and *CRY2* were methylated by FIONA1 in vivo (Fig. 6c; Additional file 1: Fig. S23). These findings collectively reveal that FIONA1 directly installs m^6^A on non-U6 m^6^A motifs in *PIF4*, *CRY2*, *CO*, and *FLC* transcripts in vivo.

### FIONA1-mediated m^6^A methylation in *PIF4*, *CRY2*, *CO*, and *FLC* transcripts affects post-transcriptional gene regulation

We next investigated the subsequent effects of the reduced m^6^A on *PIF4*, *CRY2*, *CO*, and *FLC* in *fiona1* mutant. We measured the transcript expression levels of these four genes in 12-day-old Col-0 and *fiona1-1* seedlings under different growth conditions. Consistent with the observed phenotypes in *fiona1* mutants, the RT-qPCR results showed that the expression levels of *CRY2* and *CO* transcripts were increased and *FLC* expression level was decreased in *fiona1-1* mutant compared with Col-0 under both LD and SD conditions (Fig. 6d; Additional file 1: Fig. S24). *PIF4* transcript expression level was increased in *fiona1-1* mutant compared with Col-0 under LD (ZT13), SD (ZT10), and continuous Rc and FRc conditions (Fig. 6d; Additional file 1: Fig. S25a). Consistently, PIF4 protein level was also increased in *fiona1-1* mutant plants (Additional file 1: Fig. S25b). Furthermore, the expression levels of these four transcripts in *fiona1-1* can be recovered in *FIONA1:FIONA1 /fiona1-1* plants but not in *FIONA1:FIONA1m/fiona1-1* (Fig. 6d; Additional file 1: Fig. S24e and S25a), revealing that the expression effect of these four transcripts was dependent on the m^6^A methylation activity of FIONA1.

The m^6^A modification in human has been confirmed to facility protein translation, mRNA degradation, and mRNA stabilization [10, 18, 65]. The m^6^A functions in Arabidopsis also have been found to promote mRNA stabilization or mRNA degradation [19, 24, 38], thus, we further performed transcription inhibition assays using actinomycin D to measure the lifetimes of these four transcripts. The results showed that in *fiona1-1* mutant, *PIF4*, *CRY2*, and *CO* transcripts were degraded more slowly and *FLC* transcript was degraded more rapidly compared to Col-0 (Fig. 6e). Thus, our results collectively demonstrate that loss-of-function of FIONA1 reduces m^6^A in *PIF4*, *CRY2*, *CO*, and *FLC* transcripts, which promotes stabilization of *PIF4*, *CRY2*, and *CO* mRNA and degradation of *FLC* mRNA and thereby leads to the observed phenotypes related in phytochrome signaling-dependent photomorphogenesis and floral transition (Fig. 6f).

Additionally, we further showed that the expression level of the downstream florigen *FT* (*FLOWERING LOCUS T*) was increased in *fiona1-1* mutant under both LD and SD conditions whereas its m^6^A level was not altered in *fiona1-1* mutant compared with Col-0 (Additional file 1: Fig. S26), confirming *FT* is not the direct m^6^A methylation target of FIONA1. Thus, the increased *FT* transcript expression is due to the positively transcription regulation from FIONA1-mediated m^6^A regulation in its upstream *FLC* and photoperiodic regulator *CRY2* and *CO* (Fig. 6f).

## Discussion

m^6^A modification is the ubiquitous mRNA modification in eukaryotes [52, 66–68]. Characterization of plant mRNA m^6^A writer complex, erasers, and readers revealed that mRNA modification m^6^A regulates developmental timing, leaf morphogenesis, flowering transition, trichome morphology, leaf growth, nitrate signaling, viral response, fruit ripening, ABA response, and salt tolerance [22–30, 69, 70]. However, whether Arabidopsis contains other independent mRNA m^6^A writers is not fully known. Here, we characterized that FIONA1 as Arabidopsis U6 snRNA m^6^A methyltransferase methylates m^6^A on non-U6 m^6^A motifs in a small subset of poly(A)^+^ RNA, and demonstrated the molecular mechanism how the m^6^A methylation activity of FIONA1 regulates phytochrome signaling-dependent photomorphogenesis and floral transition.

As U6 m^6^A methyltransferase, mammalian METTL16 and worm METT-10 install m^6^A on U6 or variant U6 m^6^A motif in pre-mRNAs of SAM synthetases, which affects splicing and subsequent SAM homeostasis [32, 42], the effect of intercellular SAM level by METTL16 or METT-10-mediated m^6^A methylation in SAM synthetases could tune other DNA/histone/RNA methylation modifications formed by SAM as a donor. It has been confirmed that some mRNA m^6^A sites written by METTL3/METTL14 complex and some cap m^6^A_m_ sites written by PCIF1 are affected by disruption of human METTL16 [46]. Here we found several unique features of FIONA1, which are distinct from mammalian METTL16 and worm METT-10. (1) FIONA1 neither installs m^6^A in the transcripts of Arabidopsis SAM synthetases (*MAT1-4*) nor affects their transcript expression levels under normal or high SAM conditions (Additional file 1: Fig. S19, 20). (2) FIONA1 can install m^6^A on plant mRNA m^6^A motifs in vitro and in vivo. We performed in vitro methylation experiments to reveal that FIONA1 not only methylates U6 m^6^A motif, but also assembles m^6^A for RNA containing GGAAC or UGUA motif (Fig. 2). The single-base m^6^A site validation by SELECT method in *fiona1-1* and WT plants further confirmed that FIONA1 can install m^6^A in UGUAU and GGACC sequences of *PIF4* mRNA and UGUAG sequence of *CRY2* mRNA in vivo (Fig. 6c). (3) The methylation activity of FIONA1 does not depend on stem-loop structure. We found FIONA1 exhibits higher activity towards U6 m^6^A motif in a linear RNA over in a stem-loop structured RNA (Fig. 2). Furthermore, we showed that FIONA1 prefers U6 m^6^A motif over plant mRNA m^6^A motifs in vitro (Fig. 2); however, we confirmed that FIONA1 indeed installs m^6^A on plant mRNA m^6^A motifs in vivo (Fig. 5e and Fig. 6c). Therefore, we speculate there might be some interaction proteins to assist FIONA1’s methylation on mRNA in vivo.

It is well known that the m^6^A writer complex comprising the key subunits MTA, MTB, and FIP37 is responsible for the majority of poly(A)^+^ RNA m^6^A methylation. More than 80% of m^6^A level in poly(A)^+^ RNA was reduced in the *fip37-4 LEC1:FIP37* plants. We found around 10~15% of m^6^A level of poly(A)^+^ RNA was decreased in the *fiona1-1* mutant plants compared with Col-0 (Fig. 1e). The m^6^A sequencing results identified 1137 hypomethylated m^6^A peaks in *fiona1-1* compared with Col-0 (Fig. 5a, b; Additional file 6: Table S4), which could be potential methylation targets of FIONA1. Considering 10,840 m^6^A confident peaks identified in poly(A)^+^ RNA of Col-0 (Additional file 1: Fig. S14a), the fraction of the potential FIONA-methylated m^6^A sites (1,137 hypomethylated m^6^A peaks) was around 10% of total m^6^A sites in Col-0 poly(A)^+^ RNA, in consistent with the 10~15% of m^6^A level decreased in the *fiona1-1* mutant plants. A comparison among the published FIP37 potential methylation targets, FIONA1 potential methylation targets, and non-FIONA1 targets revealed that FIONA1 and FIP37 methylate different m^6^A sites and FIP37 targets are largely overlapped with non-FIONA targets (Additional file 1: Fig. S17). Collectively, our results suggest that FIONA1 methylates no more than 15% of m^6^A sites in poly(A)^+^ RNA; the rest m^6^A sites in poly(A)^+^ RNA are installed by the m^6^A writer complex containing MTA/MTB/FIP37.

The function of m^6^A in U6 snRNA was predicted to affect splicing [32, 60]. Although mammalian METTL16 and worm METT-10 were discovered as U6 m^6^A methyltransferase, their regulatory functions on U6 still remain unclear. Here we preliminarily investigated whether m^6^A in U6 affects splicing. Our analysis showed that disruption of FIONA1 only affects 43 genes’ alternative splicing events and none of them are related with the observed phenotypes (Additional file 8: Table S6), suggesting removal of U6 m^6^A does not globally affect splicing. Our results rule out the possible effects of loss of U6 m^6^A methylation on the phenotypes in *fiona1*-1 mutant. The m^6^A function on U6 snRNA needs further exploration.

FIONA1 was previously found to affect circadian rhythms and daylength-dependent flowering and hypocotyl growth [47]; however, the underlying molecular mechanisms have not been fully illuminated. Our phenotypic analysis showed that disruption of *FIONA1* leads to early flowering under both LD and SD conditions, and its early flowering phenotype is not clearly dependent on daylength/photoperiod (Fig. 4; Additional file 1: Fig. S13). Our two *fiona1* homozygous mutant lines displays hypocotyl and its cell elongation phenotypes selectively under continuous Rc and FRc conditions, confirming that FIONA1 regulates phytochrome signaling-dependent photomorphogenesis, not daylength-dependent hypocotyl growth (Fig. 3; Additional file 1: Fig. S11, 12).

In mechanism study, we found that FIONA1 directly binds and methylates *PIF4*, *CRY2*, *CO*, and *FLC* transcripts and showed FIONA1-mediated m^6^A post-transcriptional regulation in these transcripts (Fig. 6; Additional file 1: Fig. S24, 25). The *FIONA1* transcript level is not affected by diurnal cycle and light [47], suggesting its m^6^A methylation activity is not affected by diurnal cycle and light. Indeed, our results showed that the FIONA1 regulation on *CRY2*, *CO*, and *FLC* transcripts is independent on LD and SD growth conditions (Fig. 6d; Additional file 1: Fig. S24e). CRY2 and CO are positive regulators and FLC is a negative regulator in flowering time control [62, 71–73]. CRY2 is a blue light receptor [74]; the transcript expression levels of *CO* and *FLC* are respectively regulated by photoperiod and vernalization/autonomous pathways [62, 75, 76]. FIONA1-mediated m^6^A post-transcriptional regulation in these three transcripts explained our phenotypic observation that disruption of *FIONA1* leads to early flowering that is independent on photoperiod (Fig. 4, 6; Additional file 1: Fig. S24, 26). PIF4 is phytochrome-interacting bHLH transcription factor that directly interacts with red light-activated phyB and weakly binds to far-red light-activated phyA [3, 63, 77]. PIF4 positively regulates cell elongation [1, 64, 77], and overexpression of *PIF4* causes long hypocotyl phenotype both under Rc (30 μmol m^−2^ s^−1^) and FRc (10 but not 7 μmol m^−2^ s^−1^). We found disruption of *FIONA1* reduces m^6^A on *PIF4* transcript and increases its mRNA stability (Fig. 6), in consistent with our phenotype that disruption of *FIONA1* led to cell length and long hypocotyl phenotypes selectively under Rc (30 μmol m^−2^ s^−1^) and FRc (11 μmol m^−2^ s^−1^) conditions (Fig. 3). We should note that other genes/regulators could be the methylation targets of FIONA1 that collectively contribute to the observed phenotypes in flowering and phytochrome signaling. Thus, like histone modifications, FIONA1-mediated m^6^A post-transcriptional regulation is an autonomous regulator for flowering and phytochrome signaling-dependent photomorphogenesis.

FIONA1 was reported as a genetic regulator of period length in the circadian clock, which affects the free-running circadian period of leaf movement and the period length of four central oscillator genes’ transcript expression (*CCA1*, *LHY*, *TOC1*, and *LUX*) [47]. Consistently, our GO analysis revealed that the differentially expressed genes in *fiona1-1* mutant were enriched in circadian rhythm (Fig. 5h). Our m^6^A-seq results showed that m^6^A modification level in *CCA1* and *LHY* transcripts were reduced in *fiona1-1* mutant (Additional file 6: Table S4), suggesting *CCA1* and *LHY* could be the methylation targets of FIONA1. It needs to further investigate how FIONA1-mediated m^6^A post-transcriptional regulation in central oscillator genes. Moreover, GO analysis revealed pathways in hormone responses, especially response to ethylene, cytokinin, and cytokinin-activated signaling pathway (Fig. 5h). PIF4 has been reported to transcriptionally regulate ethylene biosynthesis and to affect their signaling pathways [77]. Whether FIONA1 functions in hormone signaling and the related phenotypes through FIONA1-mediated m^6^A regulation in PIF4 or other genes remains unknown. In summary, our findings demonstrate that the m^6^A methylation of FIONA1-mediated post-transcriptional regulation is a new autonomous regulator for flowering and phytochrome signaling-dependent photomorphogenesis.

## Conclusions

FIONA1 as Arabidopsis U6 m^6^A methyltransferase is distinct from mammalian METTL16 and worm METT-10: (1) FIONA1 does not install m^6^A in pre-mRNAs of SAM synthetases to regulate intercellular SAM level under normal and high SAM conditions; (2) FIONA1 can install m^6^A on plant mRNA m^6^A motifs in vitro  and in vivo; (3) The methylation activity of FIONA1 does not depend on stem-loop structure. Our results demonstrate that FIONA1-mediated m^6^A post-transcriptional regulation is an autonomous regulator for flowering and phytochrome signaling-dependent photomorphogenesis.

## Methods

### Generation of *fiona1* mutant by the CRISPR/Cas9 system

The *fiona1* mutant was obtained following the published protocol [48]. In brief, two pair’s primers containing two single guide RNAs (sgRNAs) sequences of *FIONA1*, the *pDT1T2* vector as template, were programed PCR amplified. The products were purified and digested with *Bsa*I and ligated into a binary vector *pHEE401E.* We transformed the *PHEE401E-2sgRNA* vector into Col-0 plants *via Agrobacterium* strain GV3101-mediated floral dip method. The seeds were collected from the T0 plants and screened on 1/2MS plates containing 25 mg/L hygromycin. The positive seedlings (T1) obtained were transplanted to soil. To analyze mutations of FIONA1, genomic DNA of rosette leaf from T1 transgenic plants grown in soil were extracted, and the fragments surrounding the target sites amplified by PCR using gene-specific primers fiona1-Mut-F-1/R-1, fiona1-Mut-F-2/R-2, respectively. We submitted purified PCR products for direct Sanger sequencing with the same primers.

### Plasmid construction

One microgram of total RNA was extracted using M5 SuperPure Total RNA Extraction Reagent (SuperTRIgent) (Mei5, China) from Col-0 seedlings and reverse transcribed into cDNA using PrimeScript RT reagent Kit with gDNA Eraser (TaKaRa, Japan). The full-length FIONA1 cDNA was amplified via 2× Phanta Master (Vazyme, China) with specific primers, which were replaced other deoxynucleotide sequences to ensure that the mRNA of *FIONA1* would not be cut by Cas9 protein, while the amino acid sequence of FIONA1 protein remained unchanged in vivo. By aligning Arabidopsis FIONA1 proteins with those of other organisms, we found conserved amino acid residues-NPPFF in the methyltransferase domain. According to previous reports, both the METTL16 PP185/186AA and F187G mutants are catalytically inactive in human and mouse [32, 45]. Therefore, two conserved amino acid residues located in the methyltransferase domain were mutated to generate a putative catalytically function-abolished form of FIONA1, FIONA1m (FIONA1P237A/F239G). The cloned native promoter (1.5 kb upstream of *FIONA1*) and full-length *FIONA1* (or *FIONA1m*) CDS were cloned into a pCAMBIA1305 vector between the *Eco*RI and *Sal*I sites via ClonExpress MultiS One Step Cloning Kit (Vazyme, China). The pCAMBIA1305 vector was modified before and contains GFP or 3× Flag tag. Thus, the expression vector containing *P*_*FIONA1*_-*FIONA1*-Flag, *P*_*FIONA1*_-*FIONA1m*-Flag, and *P*_*FIONA1*_-*FIONA1*-eGFP and *P*_*FIONA1*_-*FIONA1m*-eGFP constructs were obtained. Moreover, the clone 35S promotor and *FIONA1* fragment were also cloned into pCAMBIA1305 between the *Eco*RI and *Sal*I sites to generate *P35S*-FIONA1-eGFP. Schematic representations of the constructs are shown in Additional file 1: Fig. S4a-c. All constructs were confirmed by Sanger sequencing, and the primers used in their generation are shown in Additional file 9: Table S7.

### Plant materials and growth conditions

*Arabidopsis thaliana* genotypes in this study included wild-type Col-0 and the following two representative mutant lines: *fiona1-1, fiona1-2*. All of the mutant lines were in Col-0 background and obtained via CRISPR/Cas9 editing system. The complementation lines (*FIONA1:FIONA1-GFP*/*fiona1-1, FIONA1:FIONA1m-GFP*/*fiona1-1, FIONA1:FIONA1-Flag*/*fiona1-1* and *FIONA1:FIONA1m-Flag*/*fiona1-1*) were obtained by transforming plasmids into the *fiona1-1*. The transgenic lines described are simplified as *FIONA1:FIONA1/fiona1-1* and *FIONA1:FIONA1m/fiona1-1* in this paper.

Surface-sterilized Arabidopsis seeds were plated on half-strength Murashige–Skoog (1/2 MS) (PhytoTechnology Laboratories, USA) and incubated at 4 °C for 2 days. The seeds then were grown the under different lighting conditions. In terms of flowering phenotype, the seeds were grown at 22 °C under long-day (LD) conditions (16 h light/8 h dark, 80 μmol m^−2^ s^−1^) or short-day (SD) conditions (8 h light/16 h dark, 350 μmol m^−2^ s^−1^) for 12 days, then the seedlings were transferred into the soil until they bloom under same conditions, respectively. To observe the morphology of hypocotyl, the plants were grown under continuous red light (30 μmol m^−2^ s^−1^), far-red light (11 μmol m^−2^ s^−1^), blue light (2 μmol m^−2^ s^−1^), white light (30 μmol m^−2^ s^−1^), or darkness at 22 °C.

Seedlings were collected from 12-day-old plants grown on 1/2MS nutrient agar plates at Zeitgeber time 13 (ZT13) of LD condition and Zeitgeber time 10 (ZT10) of SD condition. The rosette leaves and cauline leaves were harvested at flowering time. Flowers and buds were collected randomly from 5-week-old plants and divided into three biological replicates. The root and juvenile siliques were collected from 2-week-old seedlings and 7-week-old plants respectively. All samples described above were used for LC-MS/MS or RT-qPCR analyses. In addition, 12-day-old seedlings were collected every 3 h during one photoperiod cycles under LD and SD conditions, and subsequently the expression levels of photoperiod - related genes were detected via RT-qPCR for the diurnal course. The primers used for RT-qPCR are shown in Additional file 9: Table S7.

### Phenotypic analysis

The epidermal cells of hypocotyl were photographed by laser scanning confocal microscopy (Zeiss LSM 700, Germany). The hypocotyl length and cell length were measured using ImageJ software (http://imagej.nih.gov/ij/).The flowering phenotype under LD and SD conditions and hypocotyl of seedlings grown under various light conditions as indicated were photographed with a digital camera. Flowering time was determined as the number of days before the first flower opened and the number of rosette leaves at flowering.

### Extraction of nuclear total RNA, U6 snRNA, and tRNA

According to a published method [78], we collected 12-day-old Col-0 and *fiona1-1* seedlings (one biological repeat per 3 g) to perform nuclear-cytoplasmic fractionation. The final cytoplasmic fraction (supernatant) and the pellet (nuclear fraction) were subjected to western blotting respectively to determine whether both of that were completely separated (Additional file 1: Fig.S3a). As a quality control of the fractionation procedure, PEPC (phosphoenolpyruvate carboxylase) and HSP90, as the cytoplasmic markers, were detected using anti-PEPC polyclonal antibody (Huaxingbio, China) and anti-HSP90 polyclonal antibody (Huaxingbio, China), and histone H3 (Sigma-Aldrich, USA) was used as the nuclear marker. An appropriate amount of SuperTRIgent was added to the isolated nucleus to extract the total RNA in the nucleus.

The 8 μg total RNA from 12-day-old Col-0 and *fiona1-1* seedlings were analyzed with 10% TBE gel analysis. The U6 or tRNA bands were cut and extracted for LC-MS/MS analysis (Additional file 1: Fig. S3b).

### LC-MS/MS for m^6^A quantification

In total, 100 or 200 ng of RNA was digested with 1 U Nuclease P1 in 40 μL of buffer containing 10% 0.1 M ammonium acetate NH_4_AC (pH 5.3) at 42 °C for 3 h, followed by the addition of 1 U Shrimp Alkaline Phosphatase (NEB, USA) and 4.5 μl 1 M of 2-(N-morpholino) ethanesulfonic acid (MES). The mixture was incubated at 37 °C for an additional 3 h. The samples were then centrifuged at 15,000 rpm for 30 min, and the aqueous phase was injected into an LC-MS/MS system. Nucleosides were separated using a UPLC pump (Shimadzu, Japan) with a ZORBAX SB-Aq column (Agilent, USA) and analyzed by MS/MS using a Triple Quad^TM^ 5500 (AB SCIEX, USA) mass spectrometer running in positive ion mode and the multiple reaction-monitoring (MRM) feature. MS parameters were optimized for m^6^A detection. Nucleosides were quantified using the nucleoside-to-base ion mass transitions of m/z 268.0 to 136.0 (A), m/z 282.0 to 150.1 (m^6^A), m/z 244.0 to 112.0 (C), m/z 284.0 to 152.0 (G), m/z 245.0 to 113.1 (U). Standard curves were generated using a concentration series of pure commercial nucleosides (Sigma-Aldrich, USA) analyzed using the same method. Concentrations of nucleosides and m^6^A/A ratio in samples were calculated by fitting the signal intensities to the standard curves.

### Expression and purification of FIONA1 protein

The MBP tag and coding sequence of FIONA1 were cloned into PET28a vector containing His tag for protein expression and purification. The MBP tag can promote the expression and stability of the recombinant protein. The recombinant plasmid containing MBP-FIONA1-His was transfected into *E. coli* strain BL-21 Gold competent cells. The *E. coli* cells were grown at 37 °C to an OD600 of 0.6–0.8, and recombinant protein expression was then induced at 18 °C with 500 nM IPTG for 20 h. Then the pellet from each 2 L culture was collected, resuspended in 30 mL of lysis buffer (10 mM Tris-HCl, pH 8.0, 500 mM NaCl, 1 mM PMSF, 3 mM DTT, 5% glycerol), and sonicated for 10 min. The sample was centrifuged at 13,000 rpm for 30 min, and the supernatant was filtered through a 0.22-μm filter (Millipore), then loaded on a Ni-NTA column (GE Healthcare). After washing in 20 ml Buffer A (10 mM Tris pH 7.9, 150 mM NaCl) and then 20 ml 8% Buffer B (10 mM Tris-HCl pH 7.9, 150 mM NaCl, 500 mM imidazole), protein was eluted by Buffer B. The collected fraction was then purified by a Superdex 75 gel-filtration column (GE Healthcare, 10 mM Tris-HCl pH 7.9, 150 mM NaCl, and 3 mM DTT. Protein was concentrated into 17.8 mg ml^− 1^ and 20% glycol was added. Aliquots of protein were frozen by liquid N_2_ then stored in − 80 °C.

### Biochemistry assay for m^6^A methyltransferase activity in vitro

The in vitro methyltransferase activity following a previously published method [31]. In short, the activity assay was performed in a standard 50 μL of reaction mixture containing the following components: 0.15 nmol RNA oligos, 1.5 nmol (for RNA1 and RNA2) or 3 nmol (for RNA3-RNA6) fresh recombinant FIONA1 protein, 0.8 mM *d*_*3*_-SAM, 80 mM KCl, 1.5 mM MgCl_2_, 0.2 U μL^− 1^ RNasin, 2 mM DTT, 4% glycerol, and 15 mM HEPES (pH 8.0). Prior to the reaction, the RNA probes were annealed with a program of (i) 90 °C for 3 min and (ii) − 2 °C/cycle for 40 cycles within 30 min. The reaction was incubated at 16 °C for 12 h. The resultant RNA was recovered by phenol/chloroform extraction followed by ethanol precipitation and was digested by nuclease P1 and alkaline phosphatase for QQQ LC/MS/MS analysis. The formation of *d*_3_-m^6^A (the nucleoside-to-base ion mass transitions of 285 to 153) in oligo RNAs through methylation was used for accurate MS quantification. The peak area ratio of *d*_3_-m^6^A versus one G in the probe (as internal control) was used to evaluate the methylation activities of FIONA1 towards different RNA oligos. The RNA oligos (RNA1-RNA6) sequence are listed in Additional file 9: Table S7.

### FTO-assisted SELECT method

The FTO-assisted SELECT method is used for determining a putative m^6^A site which is m^6^A-modified in mRNAs and lncRNAs from biological samples and the m^6^A fraction at biological sites [51]. In this method, m^6^A in RNA is selected twice in a one-tube reaction. In the first of two selection steps, an m^6^A mark hinders the ability of DNA polymerase to elongate the target sequence by preventing the addition of a thymidine on the “Up Probe” opposite to the m^6^A site. In the second selection step, m^6^A marks that are present in the RNA template selectively prohibit DNA-ligase-catalyzed nick ligation between the elongated Up Probe and Down Probe. The final elongated and ligated products are then quantified by qPCR.

Total RNA was treated with FTO following a previously published method [51]. FTO-treated and untreated total RNA were mixed with 800 fmol Up Primer, 800 fmol Down Primer, and 1 pmol dTTP in 1 × CutSmart Buffer. The primers and RNA were annealed by incubating under the following conditions: 90 °C for 1 min, 80 °C for 1 min, 70 °C for 1 min, 60 °C for 1 min, 50 °C for 1 min, and then 48 °C for 6 min hold. Then, 5 μl mixture of 0.001 U Bst 2.0 WarmStart DNA Polymerase in 1× CutSmart Buffer was added in the former mixture. The reaction was incubated at 48 °C for 20 min and kept at 35 °C. Subsequently, a 10 μl mixture containing 0.5 U SplintR ligase and 10 nmol ATP was added to the final volume 20 μl. The reaction mixture was incubated at 35 °C for 15 min, denatured at 95 °C for 5 min, and then kept at 4 °C. RT-qPCR was performed with 2 μl reaction mixture as per the DNA template. Data was analyzed with QuantStudio RT-qPCR Software v.1.3. The detected results from the m^6^A site were corrected by the neighboring control site. All primers used in SELECT are listed in Additional file 9: Table S7.

### Gene expression analysis by RT-qPCR

One microgram of total RNA was extracted from different plant tissues and reverse transcribed into cDNA according to the kit mentioned above. RT-qPCR was carried out using Hieff qPCR SYBR Green Master Mix (Low Rox) (Yeasen, China) on a ViiA 7 Dx instrument (Applied Biosystems, USA). The relative expression levels were determined based on *ACTIN2 or TUB2* as the internal control. The 2^−ΔΔCT^ method was used to calculate the gene expression levels. The qPCR primers involving all genes are listed in Additional file 9: Table S7.

### M^6^A-Seq

m^6^A sequencing was carried out according to previously described m^6^A-seq method with only slight changes [54]. Briefly, 5 μg of poly(A)^+^ RNA was enriched from total RNA of 12-day-old Col-0 and *fiona1-1* seedlings at the ZT13 stage using a Dynabeads^TM^ mRNA Direct ^TM^ kit (Invitrogen, USA). The poly(A)^+^ RNA samples were then fragmented into ~ 100-nt-nucleotide-long fragments using RNA Fragmentation Reagents (Ambion, USA). Fifty nanograms fragmented poly(A)^+^ RNA samples were as input for RNA-seq. The left mRNA samples were programmed according to Instruction Manual of N^6^-Methyladenosine Enrichment Kit (NEB, USA) for enrichment of m^6^A-containing fragments. Input poly(A)^+^ RNA and immunoprecipitated poly(A)^+^ RNA were used to construct libraries with a NEBNext Ultra II RNA Library Prep Kit. Sequencing was performed on the Illumina HiSeq X Ten platform. Read numbers for two biological replicates are summarized in Additional file 3: Table S1.

### Analysis of m^6^A-seq data

For m^6^A profiling, sequencing reads were trimmed and mapped to the reference genome (TAIR10) by using Cutadapt (v1.18 ) [79], and the length of trimmed reads ≥ 20 nt were retained. Clean reads were mapped to the reference genome (TAIR10) with HISAT2 (v2.1.0) [80]. Picard Toolkit was employed to remove PCR duplication. The m^6^A-enriched regions in Col-0 and *fiona1-1* were identified using the MACS2 [81] peak-calling algorithm based on enrichment criteria (IP/Input) ≥ 5 and FDR < 0.05. FIONA1-dependent m^6^A peaks were identified by exomePeak [82] based on enrichment criteria of fold change < 1 and FDR < 0.05. We used Bedtools [83] and python scripts (https://github.com/joybio/m6A-seq/tree/main/feature_annotation/) for peak annotation. GO functional annotations (GO enrichment) were performed using DAVID.

### Analysis of RNA-seq data

Sequencing reads were trimmed and mapped to the reference genome (TAIR10) by using Cutadapt (v1.18) [79] and HISAT2 (v2.1.0) [80], respectively. The differentially expressed genes between *fiona1-1* and Col-0 were screened by R package (edgR) based on a cutoff criterion of | log2(fold change) | > 0.48 and *P* value < 0.05. We used rMATS [61] to test the effects of *fiona1* on global splicing. The Col-0 and *fiona1-1* were compared using the --cstat parameter set to 0.1, summary outputs filtered by FDR < 0.01.

### Measurement of SAM contents

The 12-day-old seedlings were used for the SAM concentration measurements. In short, 100 mg seedlings from different treatment conditions were ground into powder with in nitrogen. We added 300 μl PBS buffer (pH 7.4) to 100 mg plant powder, and vortexed for 1 min, extracted for 30 min on ice, and then centrifuged at 4 °C for 10 min at 12,000*g*. The supernatant was filtered and then analyzed via Plant SAM ELISA Kit (Shuangying, China). After the reaction is completed according to the kit, the OD value of samples is measured at 450 nm by enzyme labeling instrument, and the concentration of SAM is calculated according to the formula. The experiments were performed in three independent biological replicates with technical triplicate.

### Gene specific of m^6^A-IP-qPCR

To determine mRNA methylation level of individual genes in different plant lines, we performed an m^6^A antibody IP-enrichment according to previous reports [54] with minor modification. The 300~400 ng of mRNA (for every biological sample) was extracted from 12-day-old Col-0 and *fiona1-1* seedlings at ZT13 seeing above m^6^A-seq method. Add 5 pg of single strand of oligonucleotide containing m^6^A as spike in to these RNA samples. Since its m^6^A level is determined, it can be used as a control for normalization. Specifically, 40 ng mRNA was saved as input sample, the rest mRNA was incubated in 200 μl IP buffer (150 Mm NaCl, 0.1% NP-40, 10 mM Tris, pH 7.4, 100 U RiboLock RNase Inhibitor ( Thermo) at 4 °C for 4 h, which contained complex of m^6^A antibody (Synaptic Systems, Germany) and Dynabeads® Protein A (Invitrogen, USA). The Dynabeads were washed three times with IP buffer, and then m^6^A IP portion was eluted by 200 and 150 μL m^6^A-elute buffer (IP buffer, 6.7 mM m^6^A, 30 U RNase inhibitor) with incubating and shaking at 4 °C for 1 h, respectively. Finally, m^6^A IP portion was washed with 50 μl IP buffer once again, the supernatant is preserved. The total 400 μl supernatant was recovered by phenol-chloroform extraction and 70% ethanol precipitation. The resultant RNA concentration was measured with Equalbit^TM^ RNA HS Assay Kit (Vazyme, China). The input mRNA was further analyzed by RT-qPCR along with the m^6^A-IP mRNA using primers listed in Additional file 9: Table S7. The relative enrichment of m^6^A in each sample was calculated by normalizing the value of amplification cycle (Cq) of the m^6^A-IP portion to the Cq of the corresponding input portion.

### In vivo RNA immunoprecipitation (RIP)-qPCR

RNA immunoprecipitation was performed according to previously described method [24]. Briefly, 12-day-old *FIONA1:FIONA1-Flag/fiona1-1* seedlings were harvested at ZT13, fixed 1% formaldehyde for 15 min under vacuum, and terminated with 150 mM glycine for additional 10 min. Two grams of fixed plant material was ground and homogenized in 2 ml of lysis buffer (50 mM Tris-HCl, pH 7.5, 150 mM KCl, 5 mM EGTA, 1 mM PMSF, 0.1 U/μl Ribolock RNase inhibitor, and 1 × Roche Protease inhibitor Cocktail). Take part of lysate was saved as the input sample. The remainder was divided into two equal volumes and subsequently immunoprecipitated with anti-Flag M2 magnetic beads (Sigma-Aldrich, USA) or normal rabbit IgG (Cell Signaling Technology, USA) bound to Dynabeads Protein A, respectively. After washing and ethanol precipitation, the recovered RNA fractions were used for reverse transcribed into cDNA to calculate the relative enrichment fold via qRT–PCR. *AT2G07689*, which does not have an m^6^A peak from m^6^A profiling data, was used as the internal control.

### mRNA stability measurements

An mRNA stability measurement assay in vivo was performed as previously described [84] with minor modification. Briefly, 12-day-old Col-0, *fiona1-1* Arabidopsis seedlings grown on 1/2 MS medium were transferred to 10-cm Petri dishes containing 1/2 MS liquid medium at ZT13. After 30 min incubation, 0.2 mM actinomycin D was added to the buffer. The tissues were collected at 1 h after the transcription inhibitor was added; these samples are referred to as 0 h samples. The 3 h and 6 h samples were collected and immediately frozen in liquid nitrogen. The total RNA was isolated from these tissues at 3 different time points, and the remaining mRNA levels were quantified by RT-qPCR with gene-specific qPCR primers (Additional file 9: Table S7). 18S RNA was used as the internal control, and *AT2G07689* was used as a negative control [24].

## Supplementary Information

**Additional file 1: Figure S1.** Phylogenetic relationships and sequence alignment of METTL16 proteins among different species. **Figure S2.** The generation of *fiona1* mutants by CRISPR/Cas9 genome editing. **Figure S3.** Separation of nuclear-cytoplasmic fractions, U6 snRNA, and tRNA. **Figure S4.** The transgenic plants associated with *FIONA1.* **Figure S5.** The FTO-assisted SELECT for identification of m^6^A site in U6 snRNA. **Figure S6.** LC-MS/MS quantification of the m^6^A/A ratios in rRNA and tRNA isolated from Col-0 and *fiona1-1* seedlings. **Figure S7.** Relative expression levels of m^6^A-related regularity genes in Col-0 and *fiona1-1* plants. **Figure S8.** SDS-PAGE gel showing the purified recombinant Arabidopsis FIONA1 proteins for *in vitro* methylation assays. **Figure S9.** FIONA1 is a nuclear localized protein in Arabidopsis. **Figure S10.** FIONA1 is ubiquitously expressed in diverse Arabidopsis tissues. **Figure S11.** Hypocotyl phenotypes of the indicated genotypic seedlings under continuous blue light, white light, and dark. **Figure S12.** Disruption of *FIONA1* leads to hyposensitivity of *fiona1* mutants to red and far-red lights. **Figure S13.** Disruption of *FIONA1* leads to early flowering. **Figure S14.** Transcriptome m^6^A profiling in Col-0 and *fiona1-1*. **Figure S15.** Representative integrative genomics viewer of hypomethylated m^6^A peaks in *fiona1-1* and verification of m^6^A-seq results. **Figure S16.** m^6^A-binding motif identified by MEME. **Figure S17.** Differences in methylation sites between FIONA1 and m^6^A writer complex containing MTA/MTB/FIP37. **Figure S18.** Homologous sequence alignment between mammalian *MAT2A* gene and Arabidopsis SAM synthetases *MAT1* (*AT1G02500*), *MAT2* (*AT4G01850*), *MAT3* (AT2G36880) and *MAT4* (*AT3G17390*). **Figure S19.** m^6^A level and transcriptional expression results of SAM synthetase genes *MAT1* (*AT1G02500*), *MAT2* (*AT4G01850*), *MAT3* (*AT2G36880*) and *MAT4* (*AT3G17390*) in *fiona1-1* and Col-0 plants. **Figure S20.** FIONA1 does not affect the transcript expression levels of Arabidopsis SAM synthetases under normal and high SAM conditions. **Figure S21.** Genomics viewer showing the m^6^A-seq results for *PIF4*, *CRY2*, and *FLC* mRNA in Col-0 and *fiona1-1*. **Figure S22.** The FTO-assisted SELECT for identification of m^6^A sites in *PIF4* and *CRY2* mRNA. **Figure S23.** SELECT for identification of the FIONA1-targeted m^6^A sites in *PIF4* and *CRY2* transcripts. **Figure S24.** The expression level of *CRY2*, *CO*, and *FLC* in the indicated genotypic plants. **Figure S25.** The relative expression levels of *PIF4* in the indicated genotypic plants under SD, Rc, and FRc. **Figure S26.** The expression level of *FT* in the indicated genotypic plants.

**Additional file 2.** Uncropped western blotting and gel analysis.

**Additional file 3: Table S1.** Statistics of mapping rate.

**Additional file 4: Table S2.** Confident m^6^A peaks identified by m^6^A-seq in WT.

**Additional file 5: Table S3.** Confident m^6^A peaks identified by m^6^A-seq in *fiona1-1*.

**Additional file 6: Table S4.** Hypomethylated m^6^A peaks identified in *fiona1-1*.

**Additional file 7: Table S5.** Differentially expressed genes identified in *fiona1-1* compared to WT by RNA-seq.

**Additional file 8: Table S6.** Summary of splicing events altered in the *fiona1-1*.

**Additional file 9: Table S7.** Primers and oligonucleotide probes used in this study.

**Additional file 10.** Review history.

## Data Availability

The raw sequencing data reported in this paper have been deposited in the Genome Sequence Archive in the National Genomics Data Center (NGDC), China National Center for Bioinformation / Beijing Institute of Genomics, Chinese Academy of Sciences (GSA: CRA004052) which is publicly accessible at https://ngdc.cncb.ac.cn/gsa [85]. The published sequencing data related with FIP37 can be downloaded from the NCBI database (GSE75508) [38]. All the other datasets supporting the conclusions in this study are included in the article and the Additional files.
