# Supplementary material for: FIONA1 is an RNA N6-methyladenosine methyltransferase affecting Arabidopsis photomorphogenesis and flowering
Source: Genome Biol. 2022 Jan 31;23:40. doi: 10.1186/s13059-022-02612-2 (PMC8802475; doi:10.1186/s13059-022-02612-2)
Supplement: Supplementary file 1 — Additional file 1: Figure S1. Phylogenetic relationships and sequence alignment of METTL16 proteins among different species. Figure S2. The generation of fiona1 mutants by CRISPR/Cas9 genome editing. Figure S3. Separation of nuclear-cytoplasmic fractions, U6 snRNA, and tRNA. Figure S4. The transgenic plants associated with FIONA1. Figure S5. The FTO-assisted SELECT for identification of m6A site in U6 snRNA. Figure S6. LC-MS/MS quantification of the m6A/A ratios in rRNA and tRNA isolated from Col-0 and fiona1-1 seedlings. Figure S7. Relative expression levels of m6A-related regularity genes in Col-0 and fiona1-1 plants. Figure S8. SDS-PAGE gel showing the purified recombinant Arabidopsis FIONA1 proteins for in vitro methylation assays. Figure S9. FIONA1 is a nuclear localized protein in Arabidopsis. Figure S10. FIONA1 is ubiquitously expressed in diverse Arabidopsis tissues. Figure S11. Hypocotyl phenotypes of the indicated genotypic seedlings under continuous blue light, white light, and dark. Figure S12. Disruption of FIONA1 leads to hyposensitivity of fiona1 mutants to red and far-red lights. Figure S13. Disruption of FIONA1 leads to early flowering. Figure S14. Transcriptome m6A profiling in Col-0 and fiona1-1. Figure S15. Representative integrative genomics viewer of hypomethylated m6A peaks in fiona1-1 and verification of m6A-seq results. Figure S16. m6A-binding motif identified by MEME. Figure S17. Differences in methylation sites between FIONA1 and m6A writer complex containing MTA/MTB/FIP37. Figure S18. Homologous sequence alignment between mammalian MAT2A gene and Arabidopsis SAM synthetases MAT1 (AT1G02500), MAT2 (AT4G01850), MAT3 (AT2G36880) and MAT4 (AT3G17390). Figure S19. m6A level and transcriptional expression results of SAM synthetase genes MAT1 (AT1G02500), MAT2 (AT4G01850), MAT3 (AT2G36880) and MAT4 (AT3G17390) in fiona1-1 and Col-0 plants. Figure S20. FIONA1 does not affect the transcript expression levels of Arabidopsis SAM synthetases under no [file 13059_2022_2612_MOESM1_ESM.docx]

**Supplementary information**

**FIONA1 is an RNA *N*^6^-methyladenosine methyltransferase affecting Arabidopsis photomorphogenesis and flowering**

Chunling Wang ^1,^ **^†^**, Junbo Yang^1,^ **^†^**, Peizhe Song^1^, Wei Zhang^1^, Qiang Lu^1^, Qiong Yu^1^, and Guifang Jia^1, 2★^

^1^Synthetic and Functional Biomolecules Center, Beijing National Laboratory for Molecular Sciences, Key Laboratory of Bioorganic Chemistry and Molecular Engineering of Ministry of Education, College of Chemistry and Molecular Engineering, Peking University, Beijing 100871, China

^2^Peking-Tsinghua Center for Life Sciences, Beijing 100871, China

**^†^**These authors contributed equally: Chunling Wang, Junbo Yang.

^★^Correspondence: [guifangjia@pku.edu.cn](mailto:guifangjia@pku.edu.cn) (G.J.)

**Supplementary Figures and Figure legends**

**
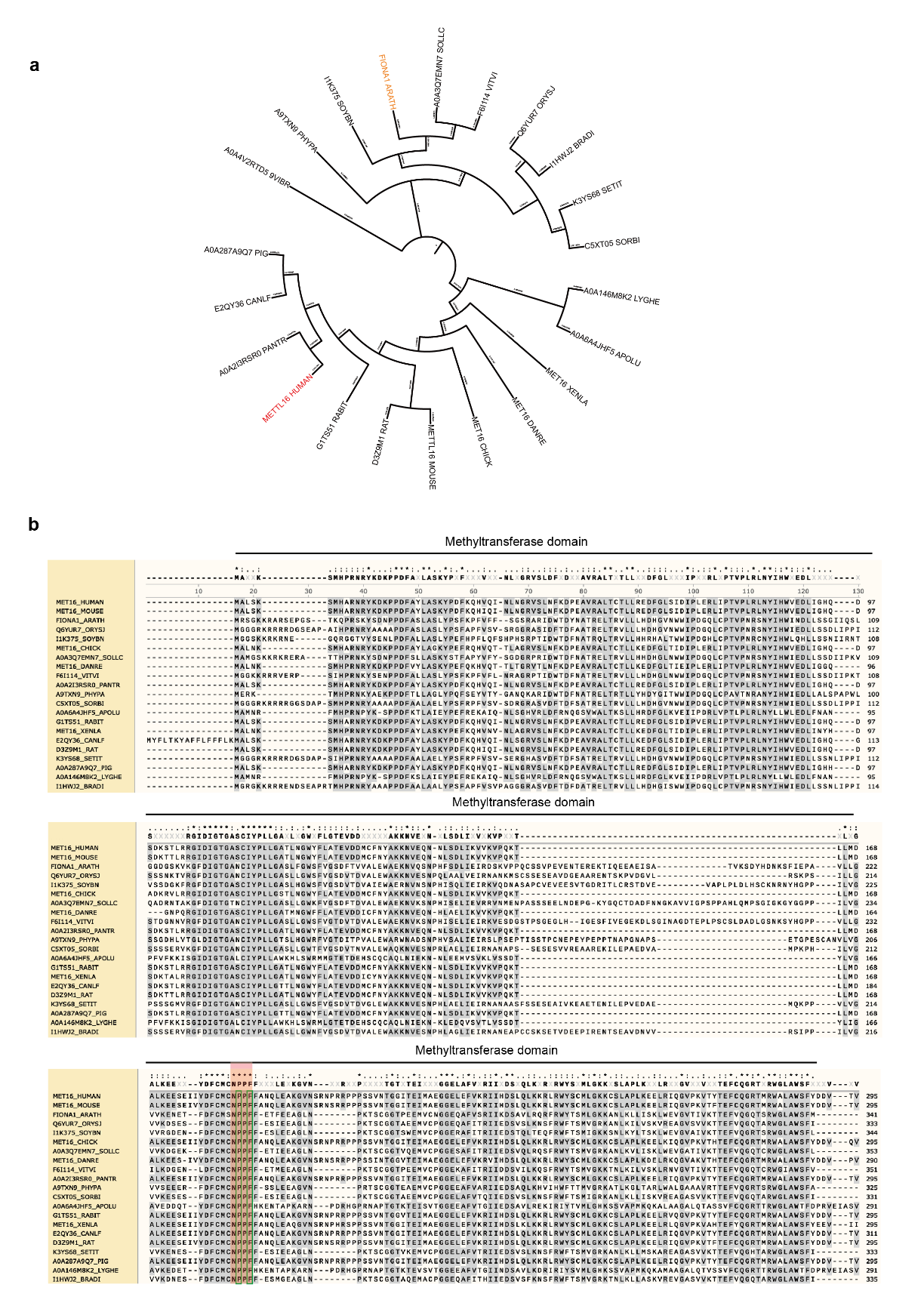
**

**Additional file 1: Fig. S1 Phylogenetic relationships and sequence alignment of METTL16 proteins among different species.** **a** The phylogenetic tree was built using human METTL16 protein compared with several other species, including *Arabidopsis thaliana* and so on. **b** Protein sequence alignment of the methyltransferase domain of human METTL16 with its homologous proteins from other organisms, including Arabidopsis FIONA1 protein. The key catalytic ligands NPPF (highlighted in pink color) are conserved among the organisms. Two sites of them (Pro237 and Phe239 in FIONA1, highlighted in the green box) were mutated to Ala and Gly, which became the catalytically inactive mutant form FIONA1 P237A/F239G (FIONA1m). The asterisk represents the same amino acid conserved among these proteins. The dot represents the level of the similarity of amino acids conserved among these proteins. The level of the similarity indicated by two dots is higher than that of one dot.


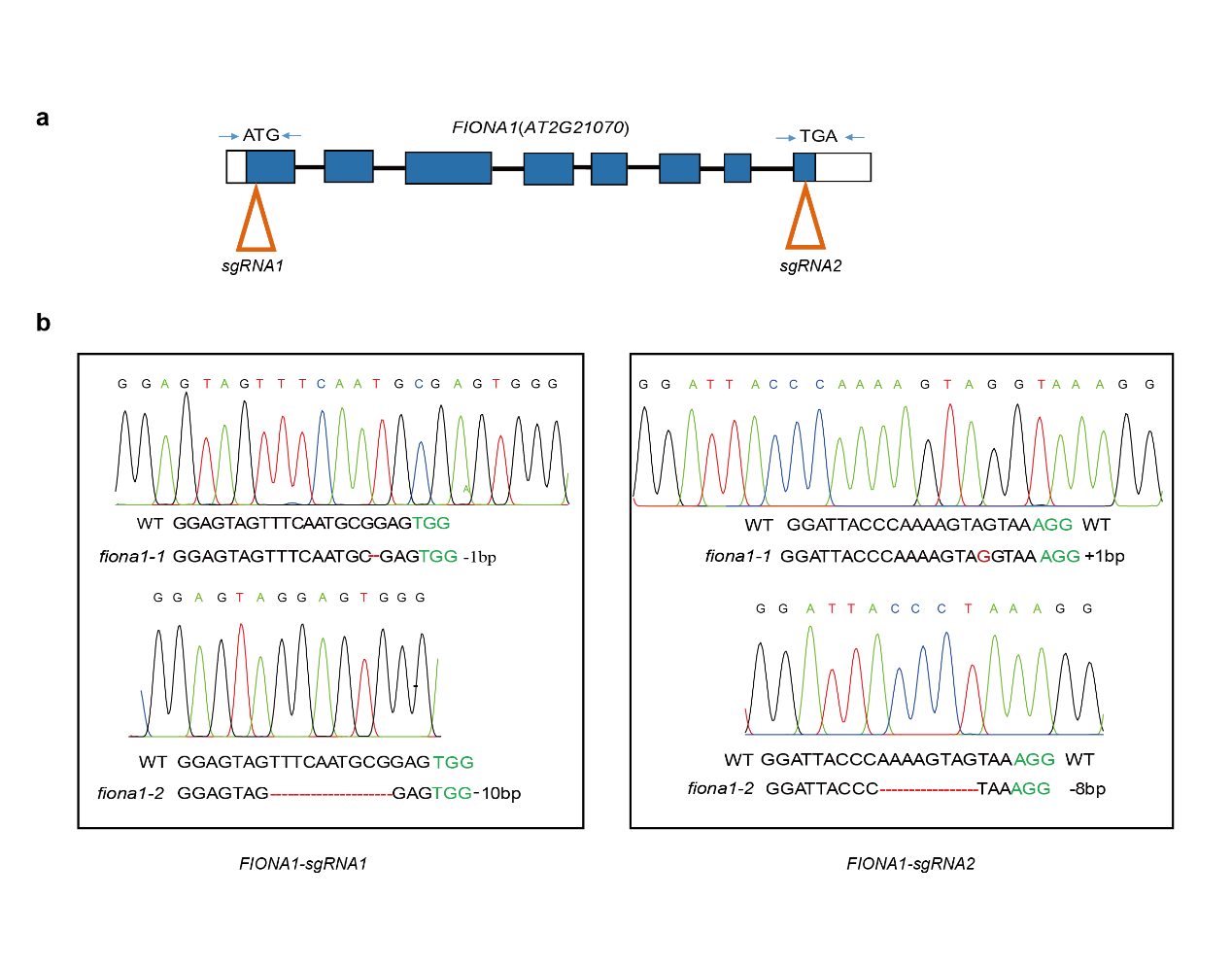


**Additional file 1: Fig. S2 The generation of *fiona1* mutants by CRISPR/Cas9 genome editing. a** Diagram shows the location of sgRNA-edited sites in FIONA1 genome (*AT2G21070*). Blue boxes: exons; Dark lines: introns; White boxes: 5′ and 3′ UTRs. Orange triangles: the editing positions of single guide RNAs (sgRNAs) used to generate CRISPR alleles. Two pairs of reverse blue arrows represent PCR primers for detecting two sgRNA-edited sites. **b** Sanger sequence chromatograms showing the sgRNA-edited nucleotide sequences in two different mutant lines. The PAM region is colored in green. The single-nucleotide deletion is marked by a red short line, one additional nucleotide is marked in red color. As shown in the figure, at the first editing site (sgRNA1), one base and 10 bases were deleted in *fiona1-1* and *fiona1-2* plants, respectively; at the second editing site (sgRNA2), One base (G) was added and eight bases were deleted in *fiona1-1* and *fiona1-2* plants, respectively.


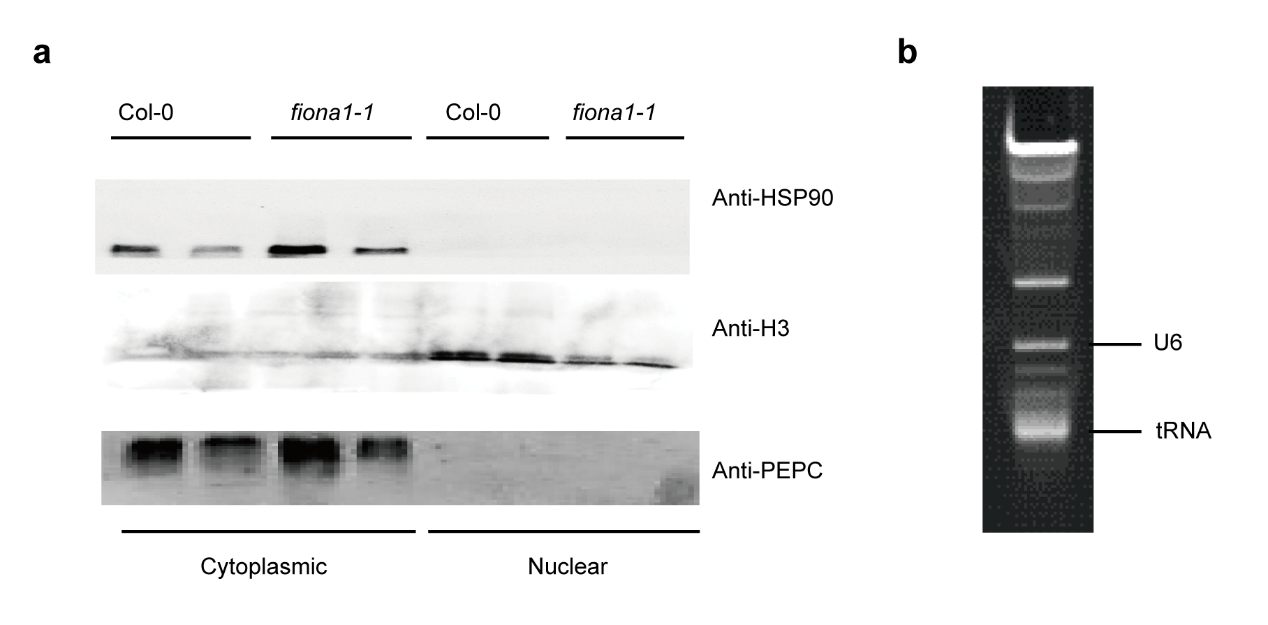


**Additional file 1: Fig. S3** **Separation of nuclear-cytoplasmic fractions, U6 snRNA, and tRNA.** **a** Western blotting validating the results of separating nuclear-cytoplasmic fractions. The cytoplasmic proteins HSP90 and PEPC and the nuclear protein histone H3 were used as indicators. **b** The 10% TEB-Urea-gel showing the isolated positions of U6 snRNA and tRNA.


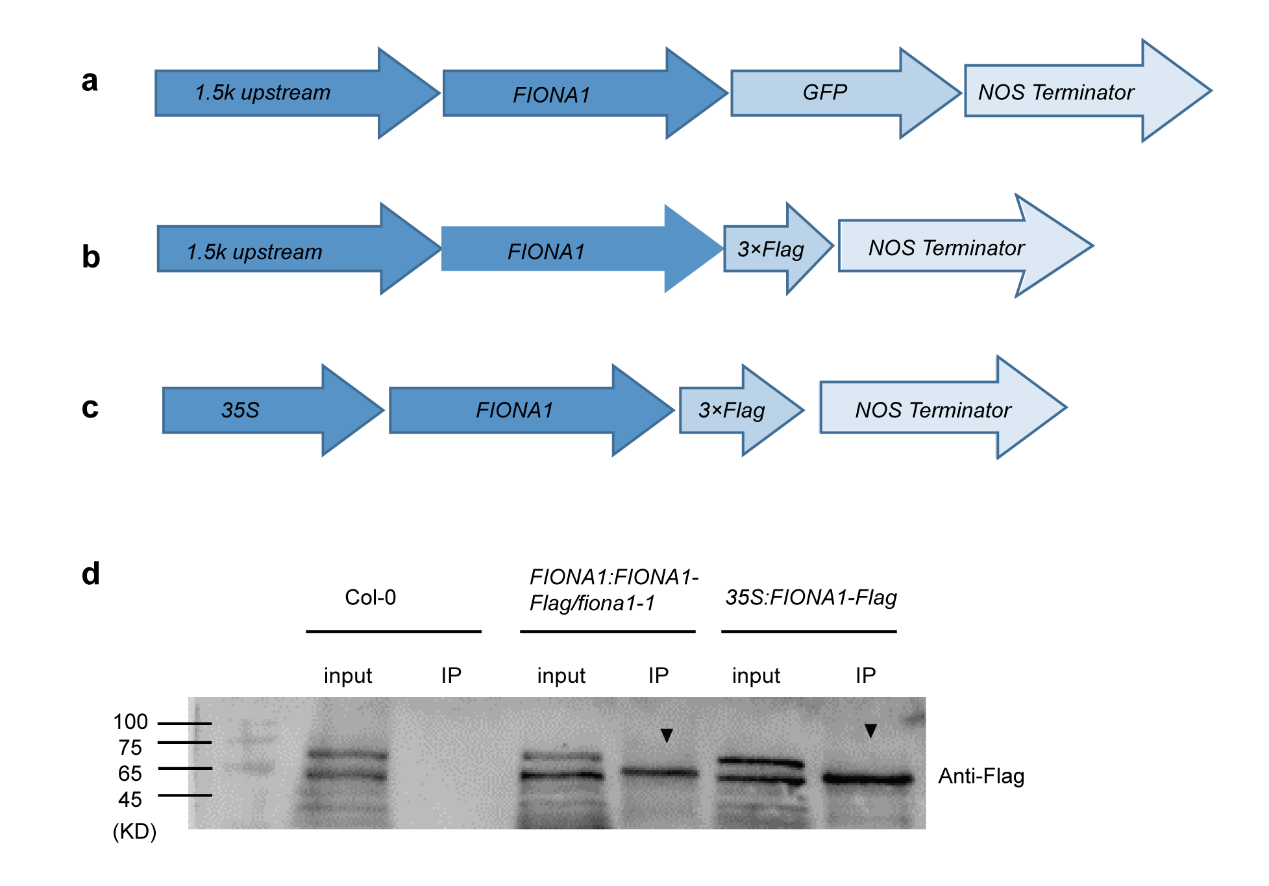


**Additional file 1: Fig. S4 The transgenic plants associated with *FIONA1.*** **a** Schematic of construct used to generate the complementation lines: *FIONA1:FIONA1-eGFP/fiona1-1* and *FIONA1:FIONA1m-eGFP/fiona1-1.* **b** Schematic of construct used to generate the complementation lines: *FIONA1:FIONA1-Flag/fiona1-1* and *FIONA1:FIONA1m-Flag/fiona1-1*. **c** Schematic of construct used to generate *35S:FIONA1-eGFP* overexpression line. **d** Western blotting showing FIONA1-Flag expressed in the *fiona1-1* complemented lines *FIONA1:FIONA1-Flag/fiona1-1* and overexpression line *35S:FIONA1-Flag* after enrichment by anti-Flag M2 magnetic beads (Sigma Aldrich, USA). The protein bands of the three input lanes are background bands, the arrow shows FIONA1-flag protein. The molecular mass of FIONA1 protein was 57,532 Da.


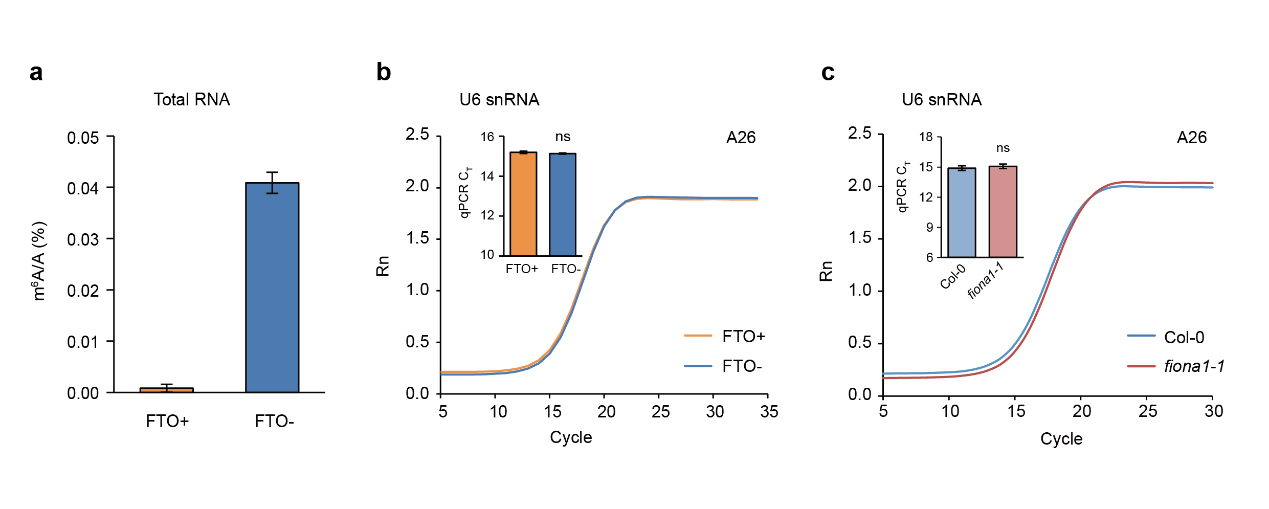


**Additional file 1: Fig. S5 The FTO-assisted SELECT for identification of m^6^A site in U6 snRNA. a** The FTO-mediated m^6^A demethylation on Arabidopsis total RNA. Data are means ± SD for 4 biological replicates × 3 technical replicates. Total RNA was isolated from 12-day-old Col-0 seedlings at ZT13. **b-c** Real-time fluorescence amplification curves and bar plot of the threshold cycle (C_T_) of qPCR showing SELECT results for detecting A26 site (for input control) in U6 snRNA with and without FTO demethylation treatment (**b)** and in Col-0 and *fiona1-1* seedlings (**c)**. Rn is the raw fluorescence for the associated well normalized to the fluorescence of the passive reference dye (ROX). Data are means ± SD for 3 biological replicates × 2 technical replicates. ns, non-significant by *t*-test (two-tailed).


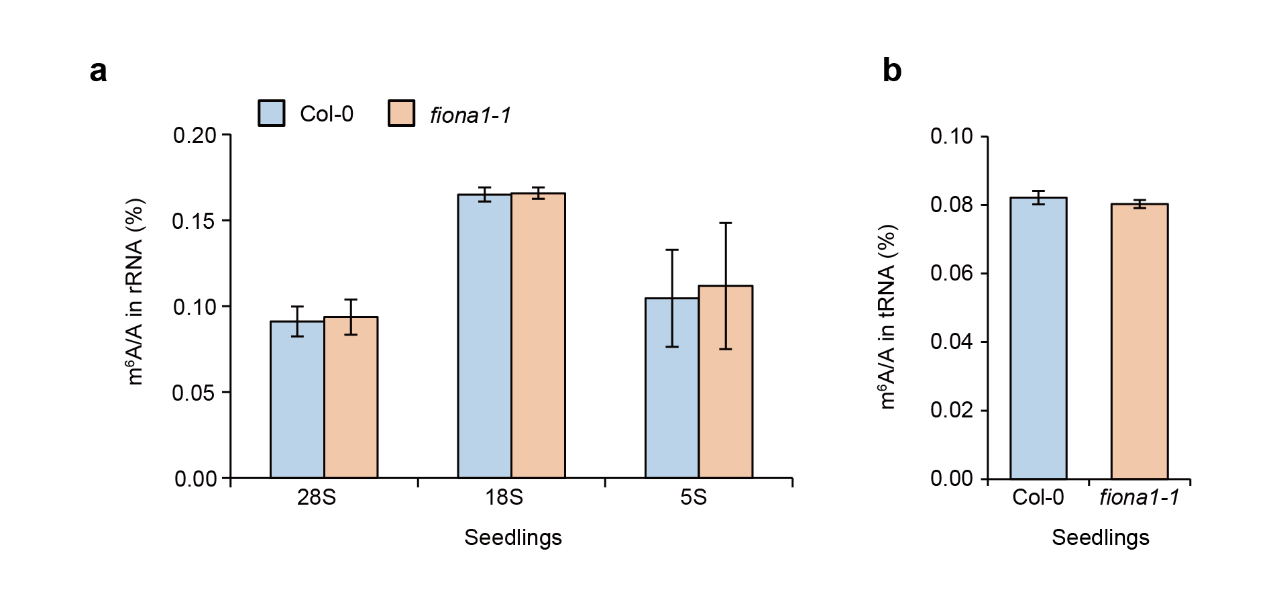


**Additional file 1: Fig. S6 LC-MS/MS quantification of the m^6^A/A ratios in rRNA and tRNA isolated from Col-0 and *fiona1-1* seedlings. a** Quantification of the m^6^A/A ratios in 28S, 18S and 5S rRNA from 12-day-old Col-0 and *fiona1-1* seedlings. **b** Quantification of the m^6^A/A ratios in tRNA from 12-day-old Col-0 and *fiona1-1* seedlings. Data are means ± SD for 3 biological replicates × 3 technical replicates.


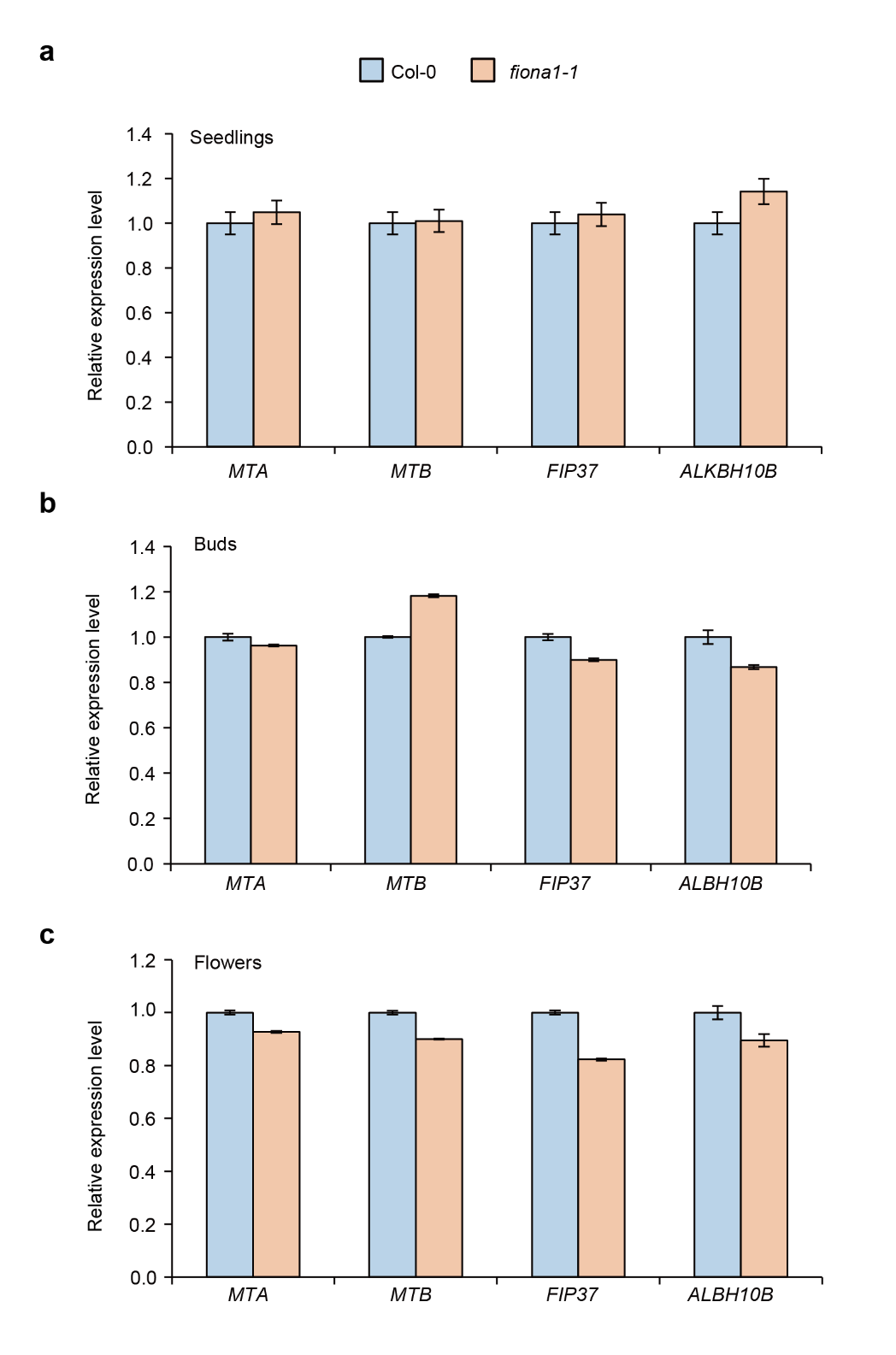


**Additional file 1: Fig. S7 Relative expression levels of m^6^A-related regularity genes in Col-0 and *fiona1-1* plants. a-c** The relative expression levels of m^6^A writer subunits (*MTA*, *MTB*, and *FIP37*) and m^6^A demethylase *ALKBH10B* in seedlings (**a)**, buds **(b)**, and flowers (**c)**. *ACTIN2* was used as a reference gene for RT-qPCR. Data is means ± SD for 3 biological replicates × 3 technical replicates.


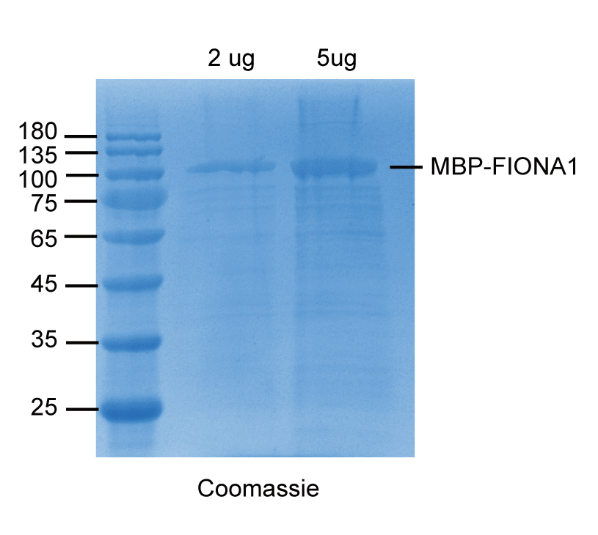


**Additional file 1: Fig. S8** **SDS-PAGE gel showing the purified recombinant Arabidopsis FIONA1 proteins for *in vitro* methylation assays.** MBP tagged FIONA1 was expressed and purified from *E. Coli*. The molecular mass of MBP tag was 42.5 kDa, and molecular mass of FIONA1 protein was 57.5 kDa.


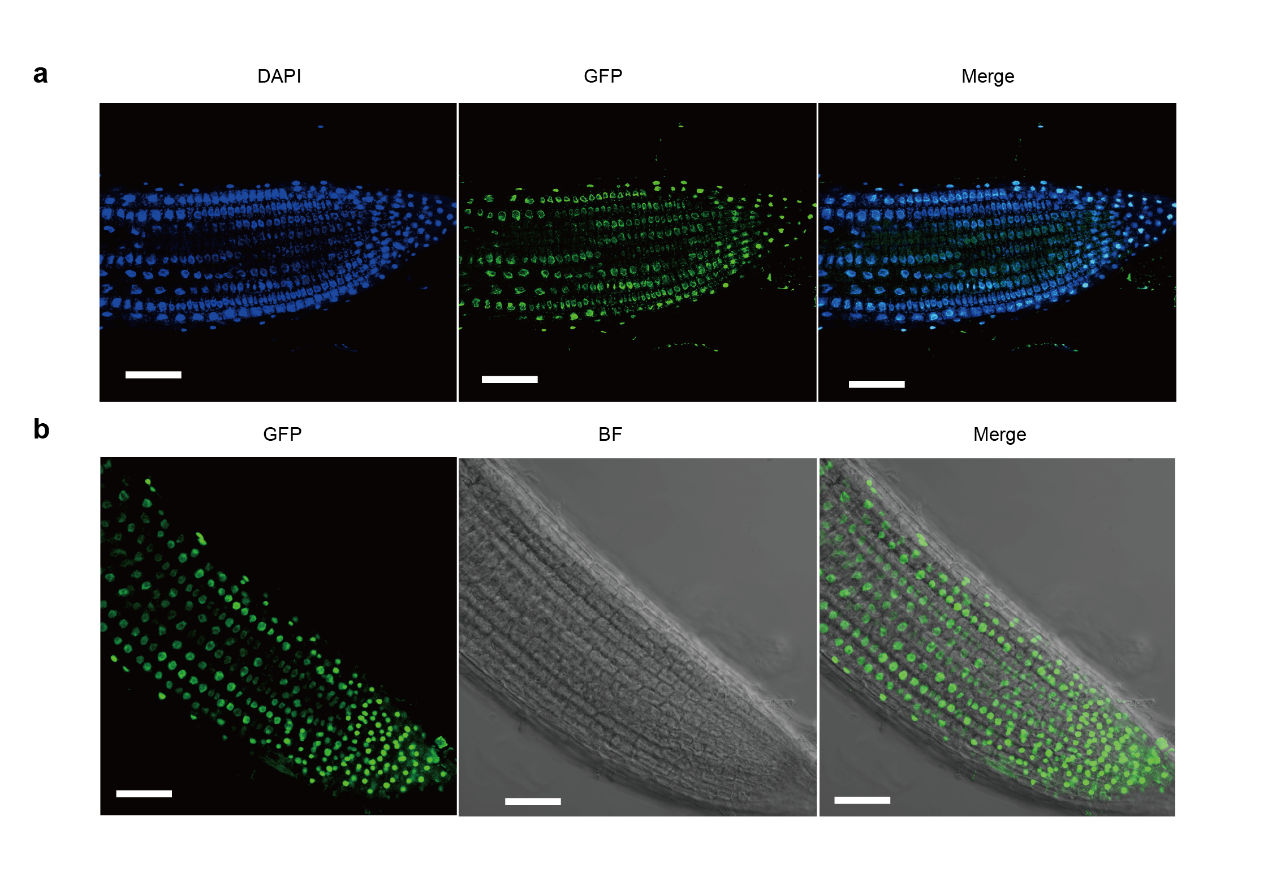


**Additional file 1: Fig. S9 FIONA1 is a nuclear localized protein in Arabidopsis.** **a-b** Confocal microscopy showing the subcellular localization of FIONA1 in root tips of 10-day-old *FIONA1:FIONA1-GFP/fiona1-1* transgenic seedlings. DAPI, 4',6-diamidino-2-phenylindole, a fluorescent dye binding to DNA; GFP, GFP fluorescence; BF, bright-field image. Scale bar = 50 μm.


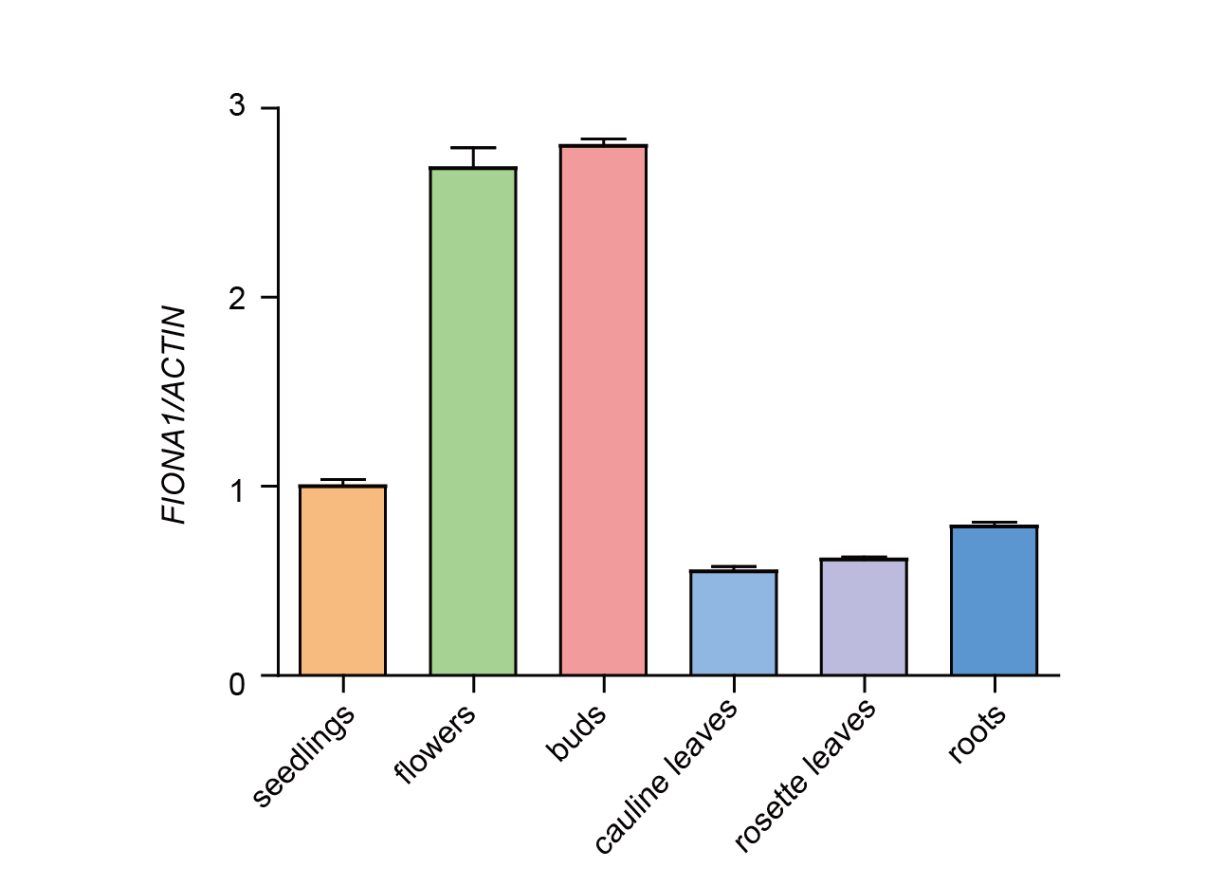


**Additional file 1: Fig. S10 FIONA1 is ubiquitously expressed in diverse Arabidopsis tissues.** Relative gene expression was measured using qPCR with *ACTIN2* as a reference gene in different tissues, prior to normalization to *FIONA1* expression levels in 14-day-old seedlings. Data are means ± SD for 3 biological replicates × 3 technical replicates.

**
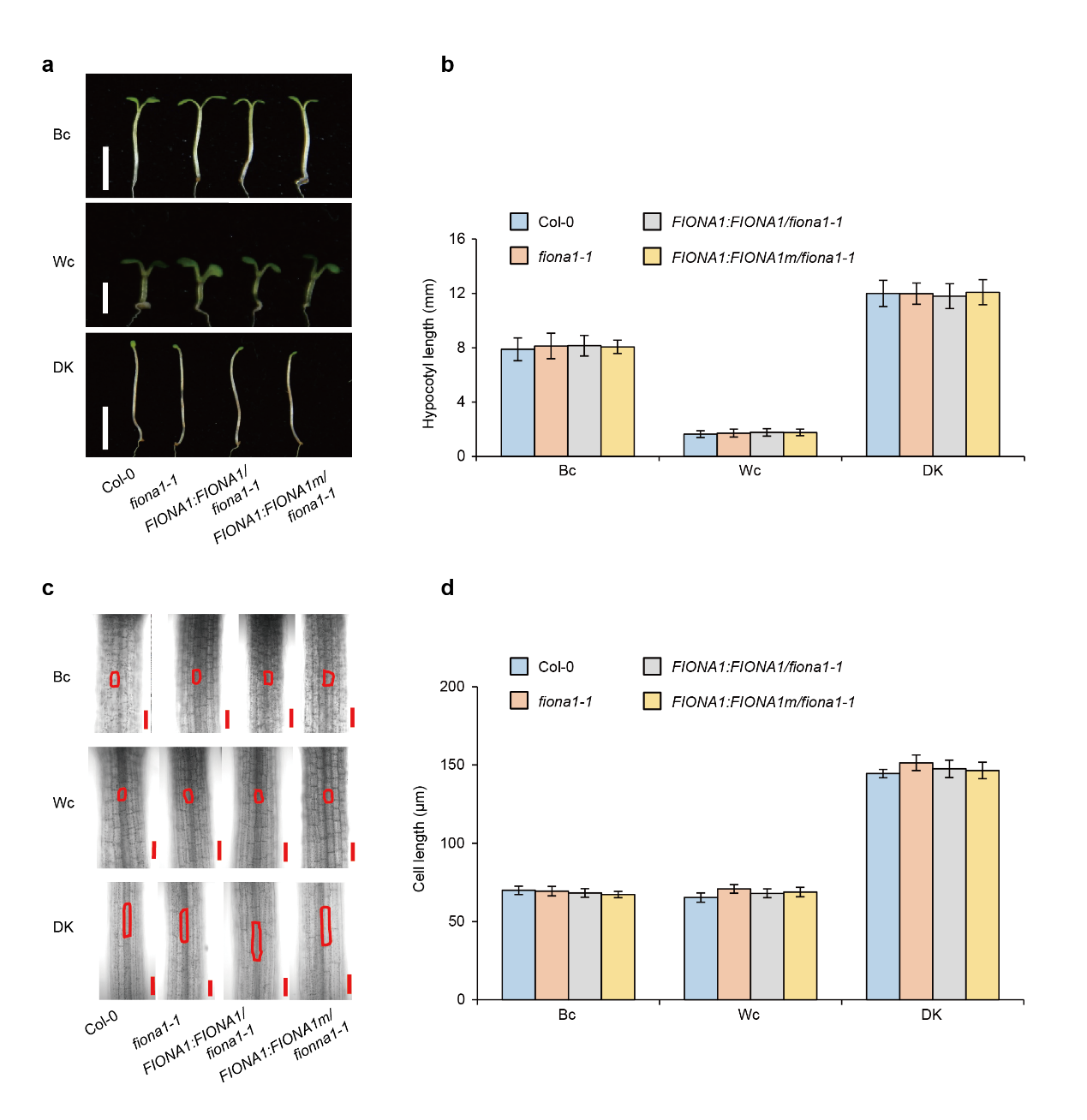
**

**Additional file 1: Fig. S11 Hypocotyl phenotypes of the indicated genotypic seedlings under continuous blue light, white light, and dark. a** Representative phenotype of Col-0, *fiona1-1*, *FIONA1:FIONA1/fiona1-1*, and *FIONA1:FIONA1m/fiona1-1* seedlings grown under Bc (Bar = 5 mm), Wc (Bar = 2 mm), and Dk conditions (Bar = 5 mm) for 7 days. **b** Hypocotyl lengths of the seedlings shown in **(a)**. Data are means ± SE (n ≥ 25). **c** Confocal microscope of hypocotyl epidermal cells of Col-0, *fiona1-1*, *FIONA1:FIONA1/fiona1-1*, and *FIONA1:FIONA1m/fiona1-1* seedlings grown in under Bc, Wc, and Dk conditions for 7 days. The cell sizes were marked with red lines. Bar = 100 μm. **d** The hypocotyl epidermal cell lengths shown in **(c)**. Data are means ± SE (n ≥ 20).


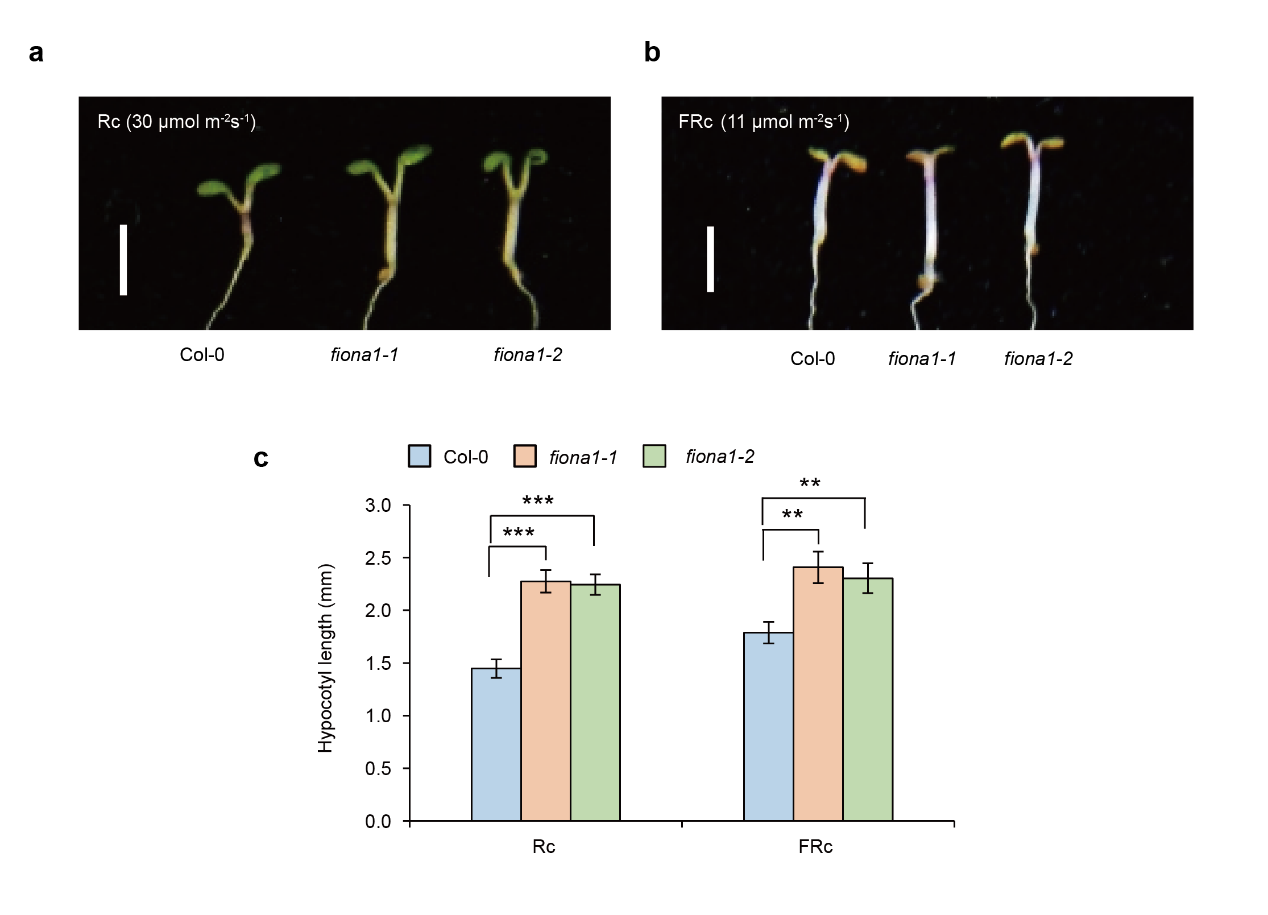


**Additional file 1: Fig. S12 Disruption of *FIONA1* leads to hyposensitivity of *fiona1* mutants to red and far red lights.** **a-b** Representative phenotypes of Col-0, *fiona1-1*, and *fiona1-2* seedlings grown under Rc **(a)** and FRc **(b)** conditions. Bar = 2 mm. **c** Hypocotyl lengths of the seedlings shown in **(a)** and **(b)**. Data are means ± SE (n ≥ 20). ** *p* < 0.05, *** *p* < 0.001 by *t* test (two-tailed).


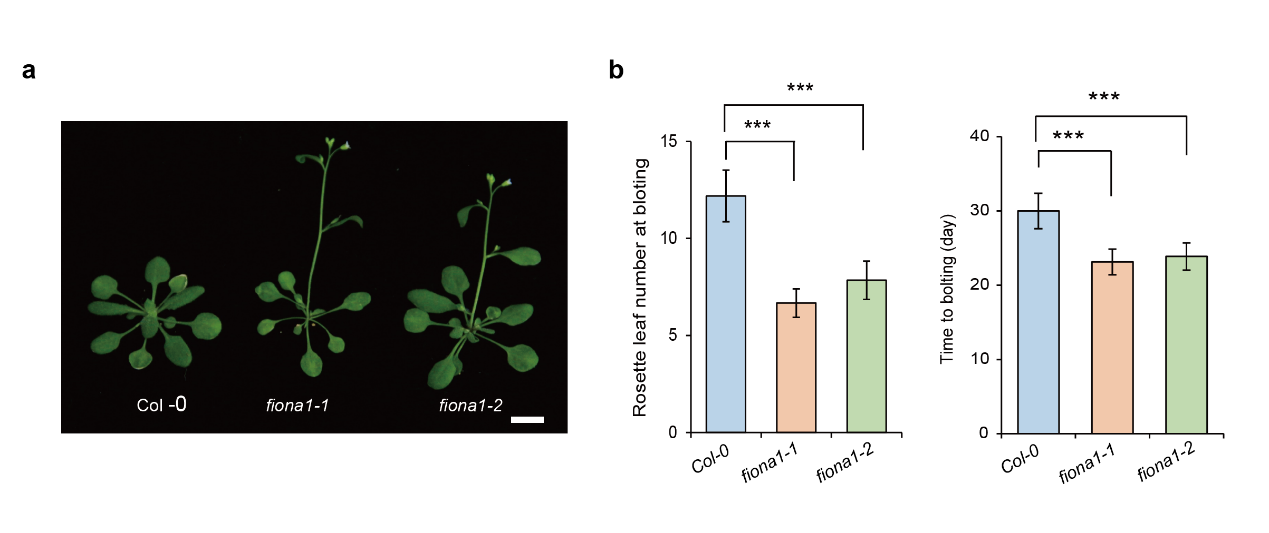


**Additional file 1: Fig. S13** **Disruption of *FIONA1* leads to early flowering.** **a** Phenotypes of the floral transition in Col-0, *fiona1-1*, and *fiona1-2* plants under LD (16L/8D) condition. Bar = 1 cm. **b** Statistical analysis of flowering time in Col-0, *fiona1-1*, and *fiona1-2* plants under LD (16L/8D) condition. Flowering time was presented as rosette leaf number and days to bolting at the bolting stage. Data are means ± SD (n ≥ 20). *** *p* < 0.001 by *t* test (two-tailed).


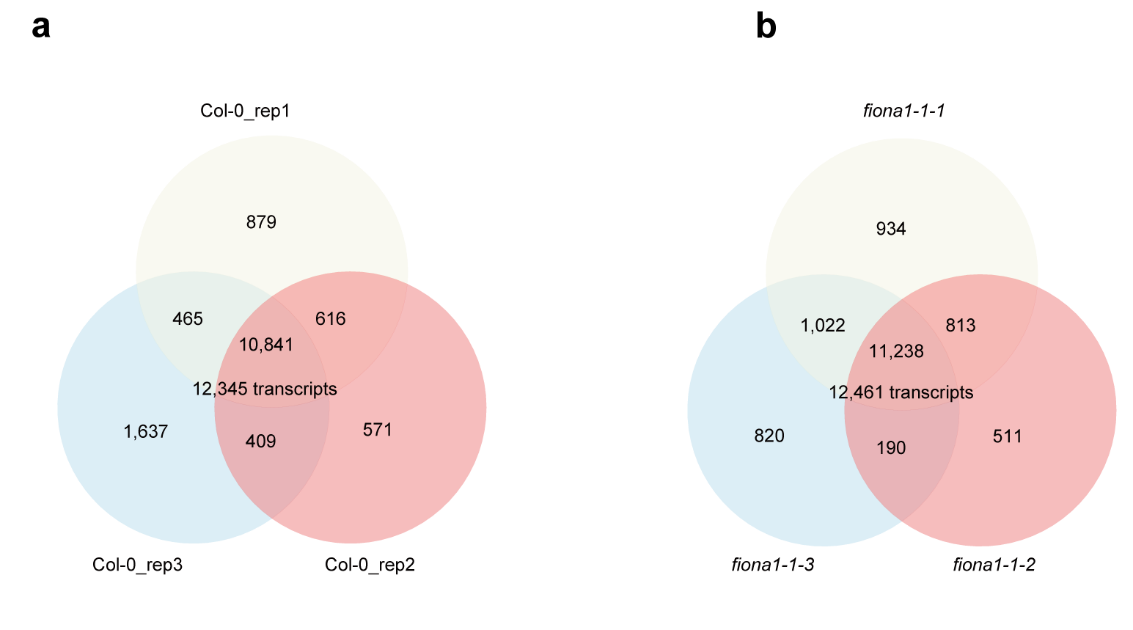


**Additional file 1: Fig. S14** **Transcriptome m^6^A profiling in Col-0 and *fiona1-1*.** **a-b** Overlapping m^6^A peaks identified in Col-0 (**a**) and *fiona1-1* (**b**) in three biological replicates.


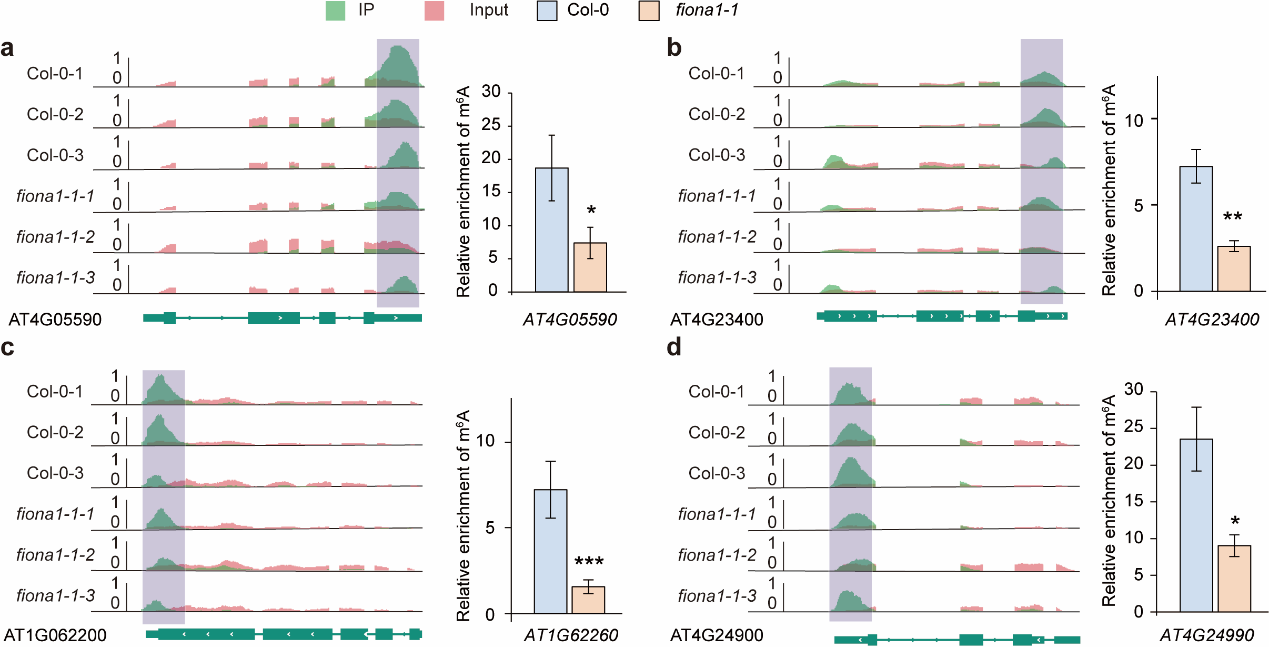


**Additional file 1: Fig. S15** **Representative integrative genomics viewer of hypomethylated m^6^A peaks in *fiona1-1* and verification of m^6^A-seq results.** **a-d** Disruption of *FIONA1* in *fiona1-1* reduces m^6^A peak levels compared to those in Col-0 seedlings from m^6^A- seq in three biological replicates and verification of m^6^A-seq results by m^6^A-IP-qPCR. Poly(A)+ RNA was isolated from 12-day-old Col-0 and *fiona1-1* seedlings. Data are means ± SE for 3 biological replicates × 3 technical replicates. * *p* < 0.05, ** *p* < 0.01, *** *p* < 0.001 by *t* test (two-tailed).


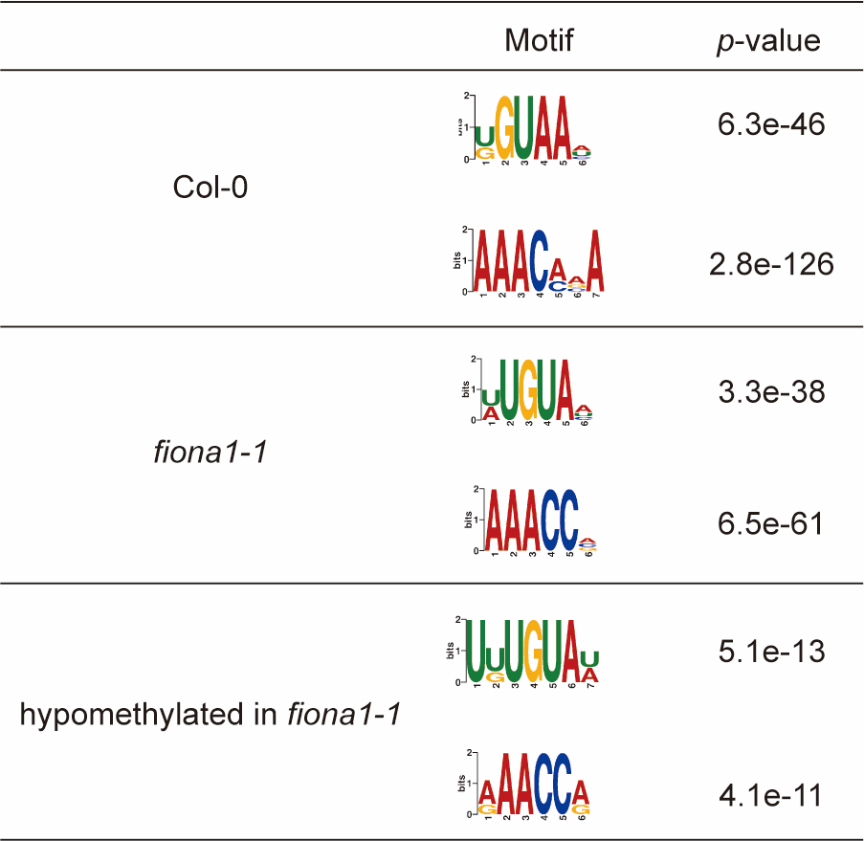


**Additional file 1: Fig. S16 m^6^A-binding motif identified by MEME.** m^6^A-binding motif identified by MEME in WT, *fiona1-1* and hypomethylated in *fiona1-1*, respectively


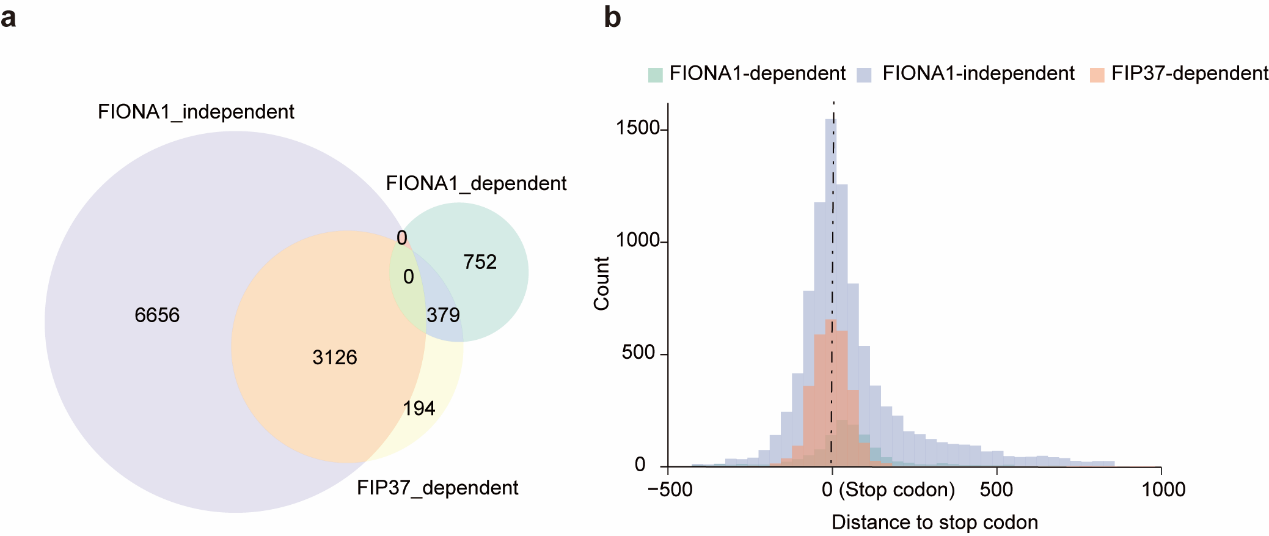


**Additional file 1: Fig. S17** **Differences in methylation sites between FIONA1 and m^6^A writer complex containing MTA/MTB/FIP37.** **a** Overlap of “FIONA1-dependent m^6^A”, “FIONA1-independent m^6^A” and “FIP37-dependent m^6^A peaks”. **b** Histogram plots showing the distance of stop codon to m^6^A sites from “FIONA1-dependent m^6^A”, “FIONA1-independent m^6^A” and “FIP37-dependent m^6^A peaks”.


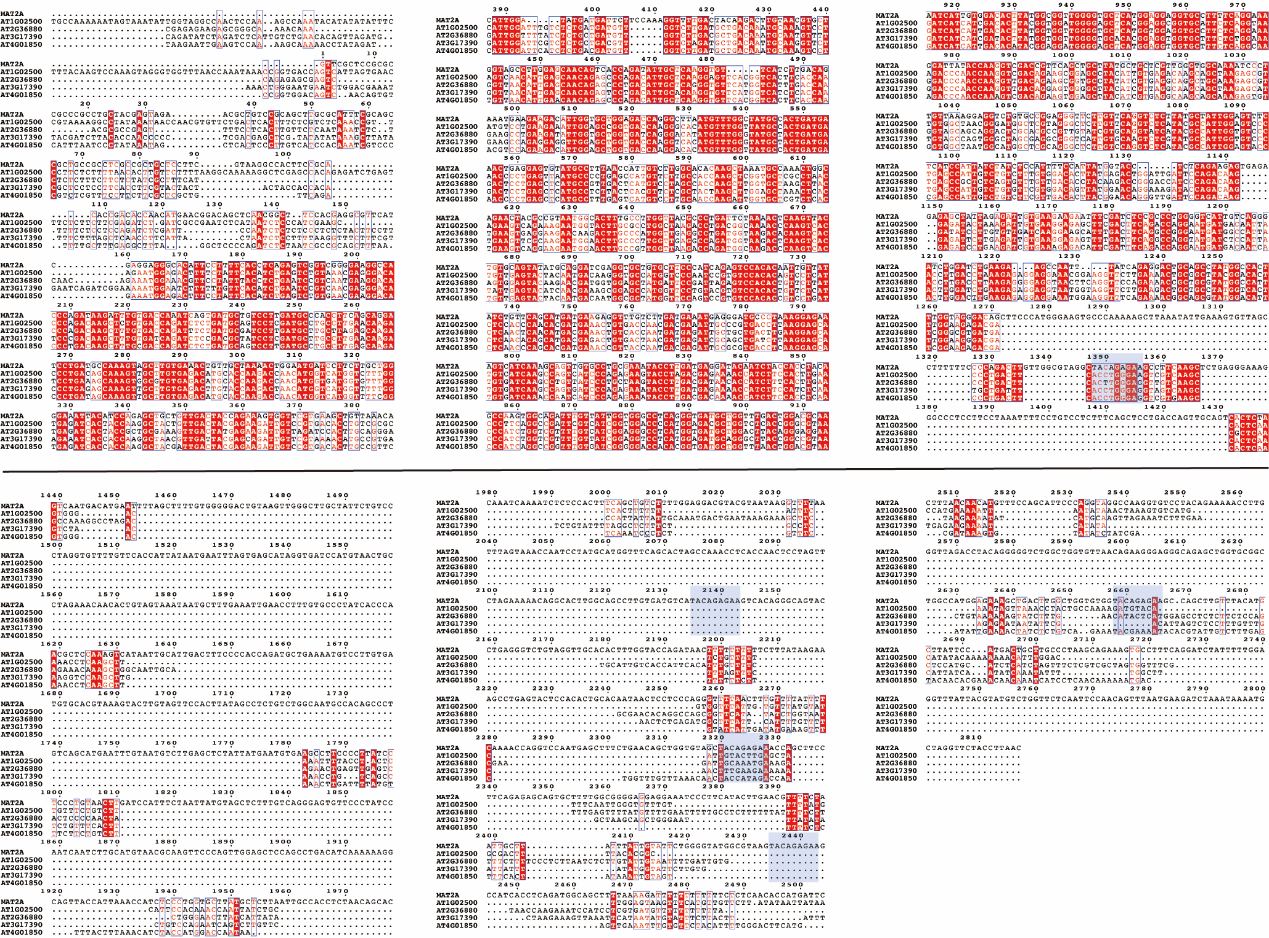


**Additional file 1: Fig. S18** **Homologous sequence alignment between mammalian *MAT2A* gene and Arabidopsis SAM synthetases *MAT1* (*AT1G02500*), *MAT2* (*AT4G01850*), *MAT3* (AT2G36880) and *MAT4* (*AT3G17390*).** The structured nonamer UACAGAGAA sequence (highlighted in light color) only exists in mammalian *MAT2A* gene, but not in Arabidopsis SAM synthetases transcripts.


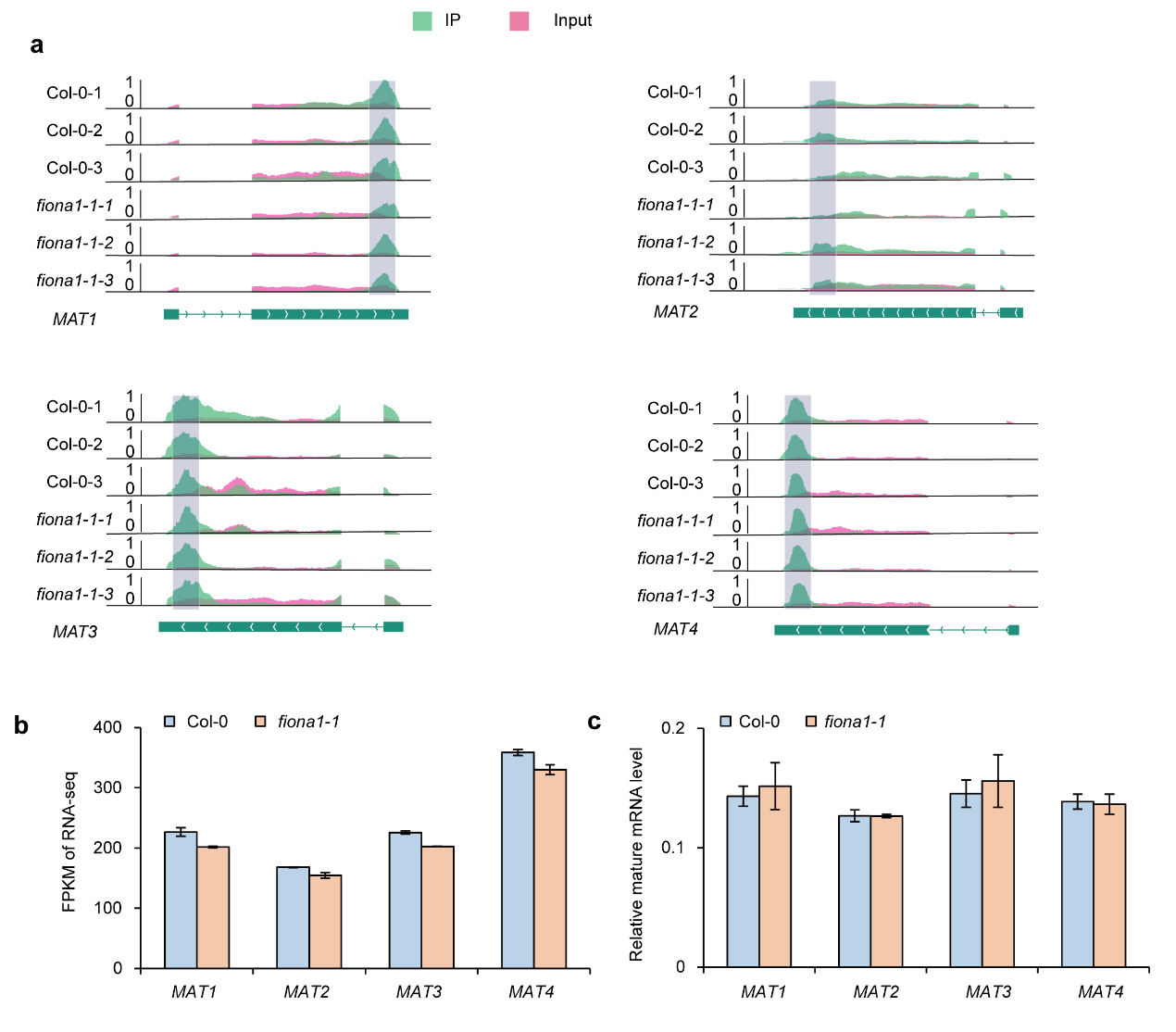


**Additional file 1: Fig. S19** **m^6^A level and transcriptional expression results of SAM synthetase genes *MAT1* (*AT1G02500*), *MAT2* (*AT4G01850*), *MAT3* (*AT2G36880*) and *MAT4* (*AT3G17390*) in *fiona1-1* and Col-0 plants. a** Integrative genomics viewer of m^6^A modification in *MAT1-MAT4* mRNA, and their m^6^A levels were not significantly reduced in *fiona1-1* compared with Col-0. **b** RNA-seq results showed no significant changes in expression levels of the above four genes between Col-0 and *fiona1-1* plants. **c** RT-qPCR results showed no significant differences in expression levels of the four SAM synthetase genes.


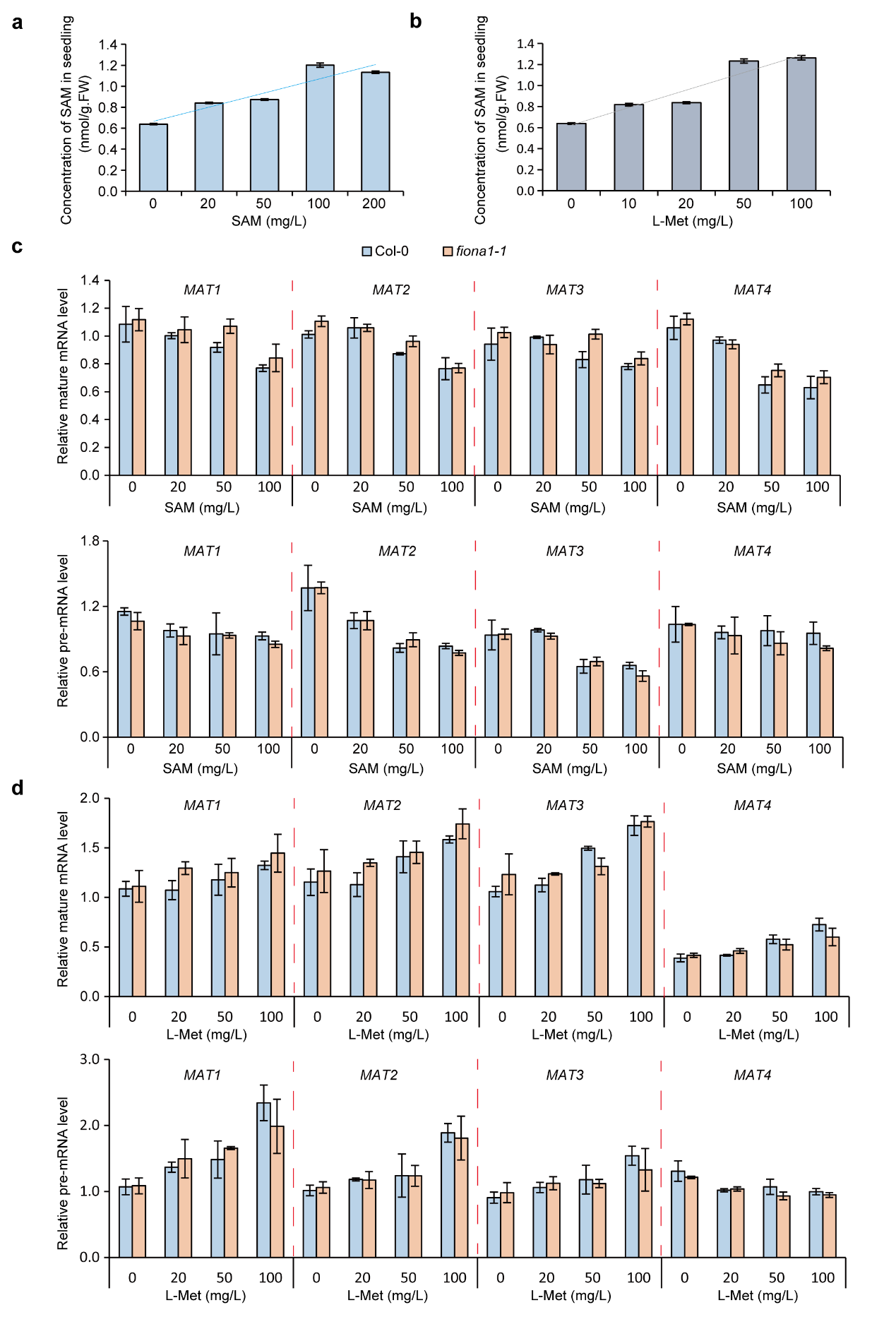


**Additional file 1: Fig. S20** **FIONA1 does not affect the transcript expression levels of Arabidopsis SAM synthetases under normal and high SAM conditions. a, b** The SAM concentration inside 12-day Arabidopsis seedlings treated with different concentrations of SAM and L-Met. **c, d** The expression levels of mature mRNA and pre-mRNA of four SAM synthase genes in 12-day *fiona1-1* and Col-0 treated with different concentrations of SAM and L-Met.


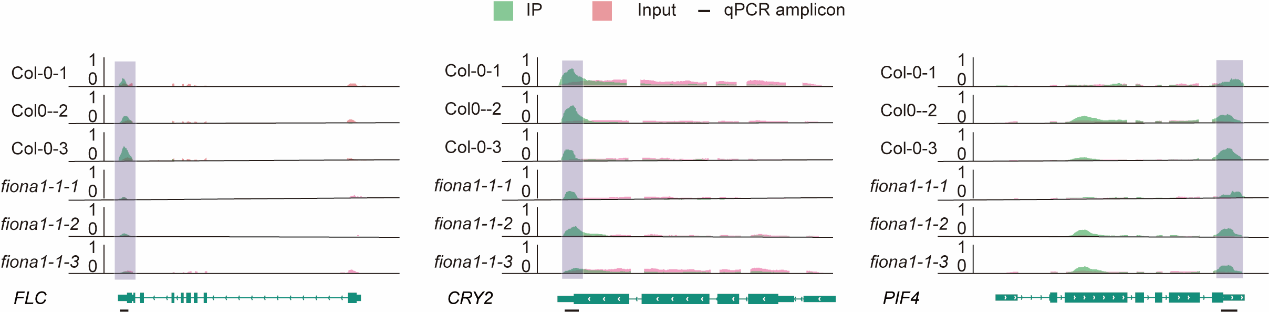


**Additional file 1: Fig. S21** **Genomics viewer showing the m^6^A-seq results for *PIF4*, *CRY2*, and *FLC* mRNA in Col-0 and *fiona1-1*.** The corresponding transcript structure is shown beneath, with thick boxes and lines representing exons and introns, respectively. The shaded region of the transcript structure shows the m^6^A peak; The black line below the transcript structure shows the amplification region of m^6^A-IP-qPCR in (Fig. 6a).


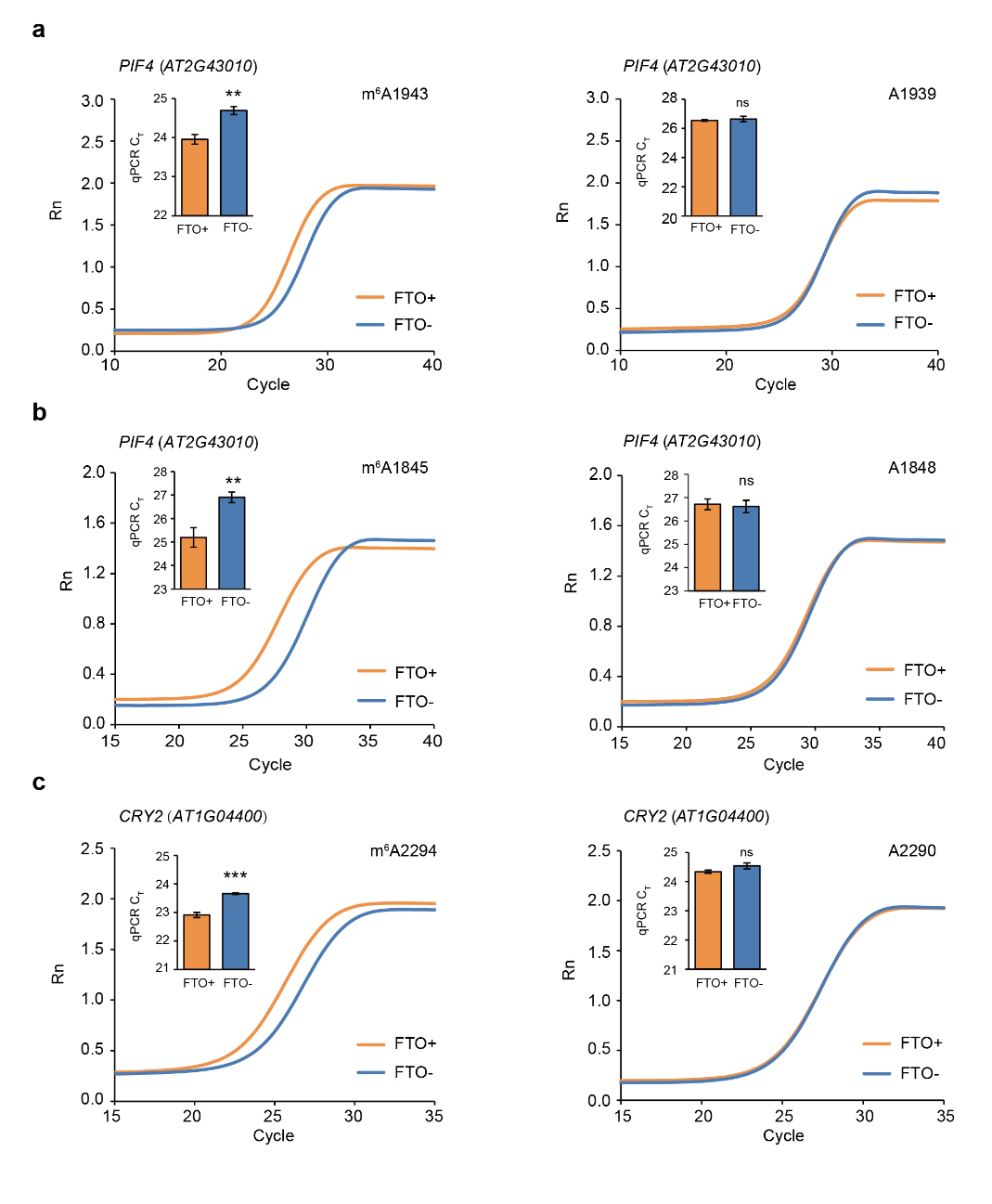


**Additional file 1: Fig. S22** **The FTO-assisted SELECT for identification of m^6^A sites in *PIF4* and *CRY2* mRNA. a-c** Real-time fluorescence amplification curves and bar plot of the threshold cycle (C_T_) of qPCR showing SELECT results for detecting m^6^A1943 and A1939 site (for input control) in *PIF4* transcript **(a)**, m^6^A1845 and A1848 site (for input control) in *PIF4* transcript **(b)**, and m^6^A2294 and A2290 site (for input control) in *CYR2* transcript **(c)**. Rn is the raw fluorescence for the associated well normalized to the fluorescence of the passive reference dye (ROX). Data are means ± SD for 3 biological replicates × 2 technical replicates. ns, non-significant, ** *p* < 0.01, *** *p* < 0.001 by *t* test (two-sided).


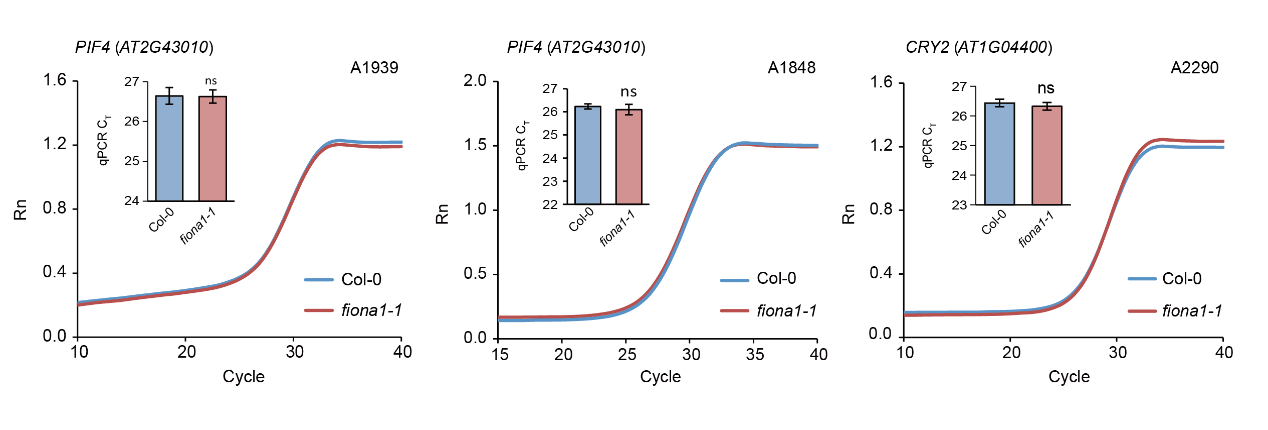


**Additional file 1: Fig. S23** **SELECT for identification of the FIONA1-targeted m^6^A sites in *PIF4* and *CRY2* transcripts.** Real-time fluorescence amplification curves and bar plot of the threshold cycle (C_T_) of qPCR showing SELECT results for detecting A1939 and A1848 sites (for input control) in *PIF4* transcript and A2290 site (for input control) in *CRY2* transcript in Col-0 and *fiona1-1* seedlings (Supports Fig. 5c). Rn is the raw fluorescence for the associated well normalized to the fluorescence of the passive reference dye (ROX). Data are means ± SD for 3 biological replicates × 2 technical replicates. ns, non-significant by *t*-test (two-tailed).


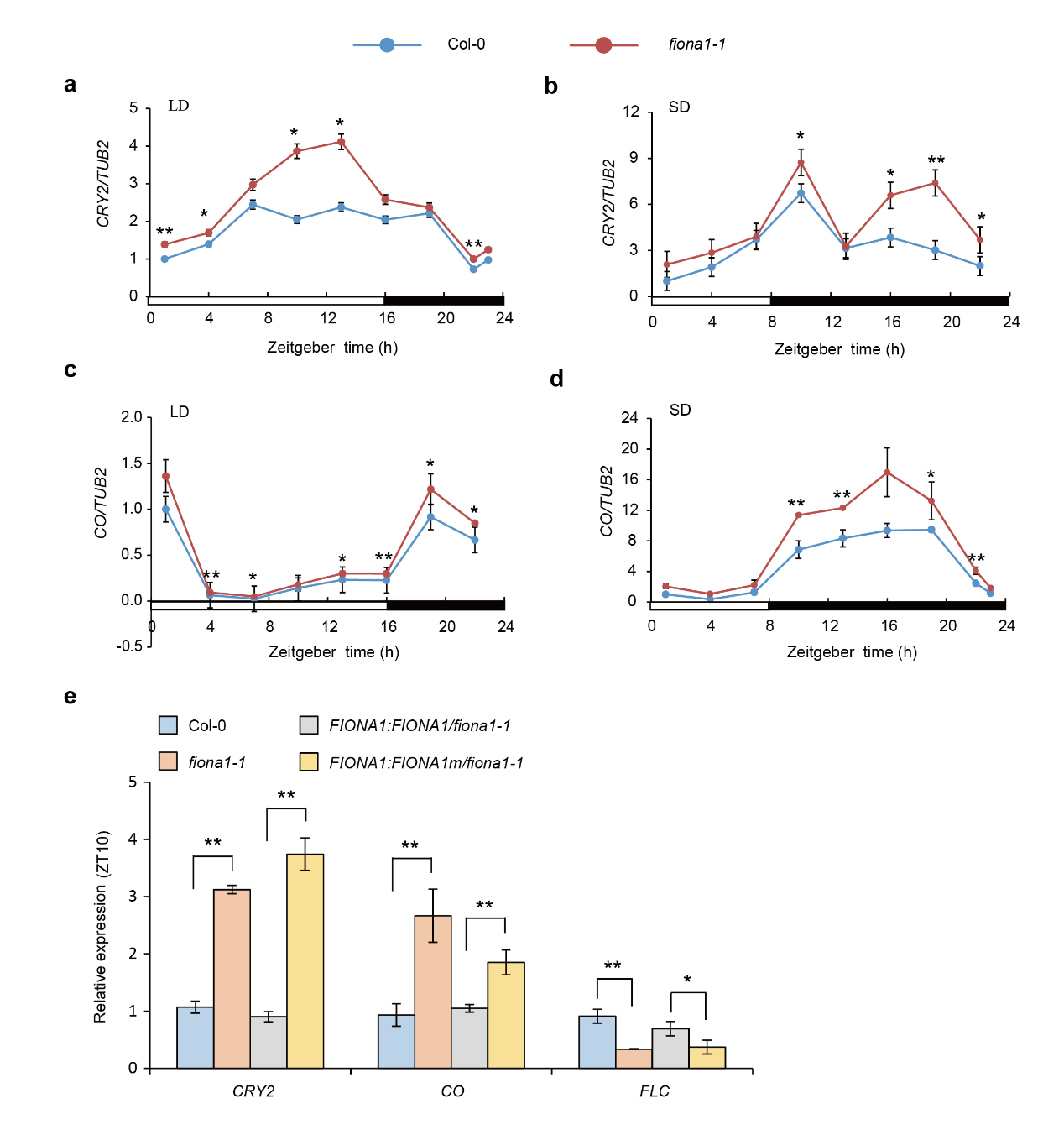


**Additional file 1: Fig. S2****4** **The expression level of *CRY2*, *CO*, and *FLC* in the indicated genotypic plants.** **a-d** Diurnal time courses of *CRY2* and *CO* expression in the Col-0 and *fiona1-1* under LD (16L/8D) and SD (8L/16D) conditions. 12-day-old seedlings were harvested at Zeitgeber time 1 (ZT1) and at 3 h intervals. **e** The relative expression levels of *CRY2*, *CO*, and *FLC* transcripts in the indicated genotypic plants at Zeitgeber time 10 (ZT10) under SD (8L/16D) conditions. *TUB2* was used as the internal control gene. Data are means ± SD for 3 biological replicates × 3 technical replicates. * *p* < 0.05, ** *p* < 0.01 by *t*-test (two-tailed).


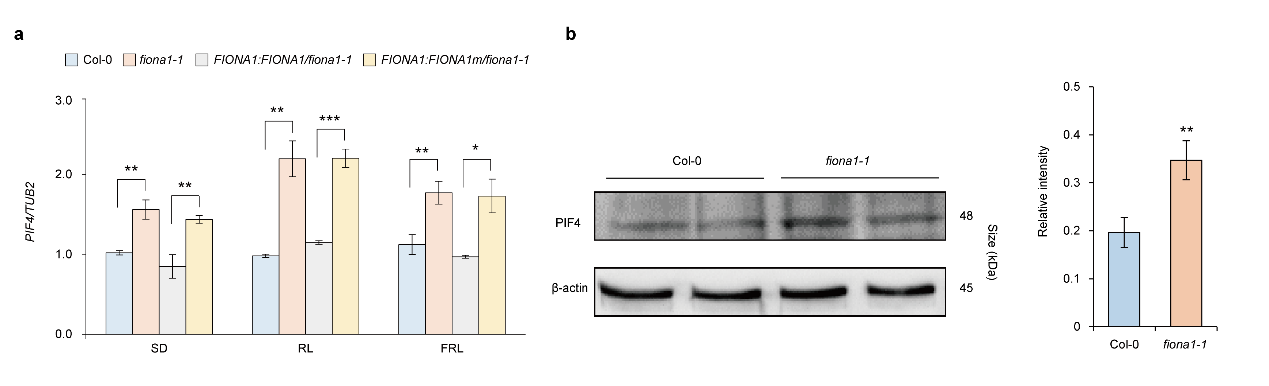


**Additional file 1: Fig. S2****5** **The relative expression levels of *PIF4* in the indicated genotypic plants under SD, Rc, and FRc. a**12-day-old seedlings were harvested at ZT10 under SD (8L/16D) conditions, and 7-day-old seedlings were collected grown under continuous red light (30 μmol m^-2^s^-1^) and far-red light (11μmol m^-2^s^-1^) conditions. Expression levels of *PIF4* were determined by real-time qPCR, and values were normalized against *TUB2* expression. Data are means ± SD for 3 biological replicates × 3 technical replicates. * *p* < 0.05, ** *p* < 0.01, *** *p* < 0.001 by *t*-test (two-tailed). **b** Immunoblot assays of PIF4 protein levels in 12-day *fiona1-1* and Col-0. The PIF4 protein levels were quantified relative to β-actin level. Data are mean ± SD (n= 4). ** *p* < 0.01 by *t* test (two-tailed).

**
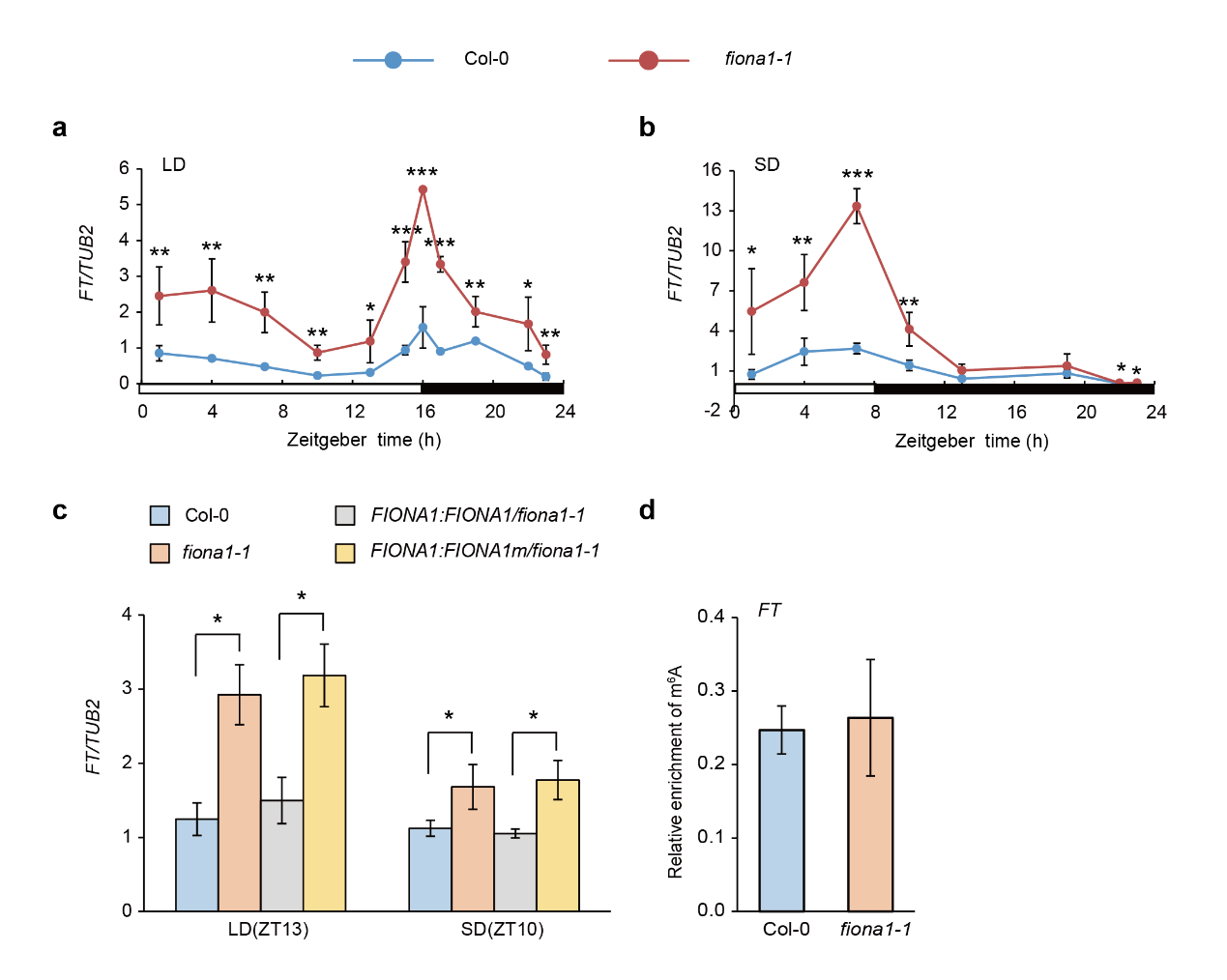
**

**Additional file 1:** **Fig. S26** **The expression level of *FT* in the indicated genotypic plants. a-b** Diurnal time courses of *FT* expression in Col-0 and *fiona1-1* under LD (16L/8D) and SD (8L/16D) conditions. 12-day-old seedlings were harvested at Zeitgeber time 1 (ZT1) and at 3 h intervals. **c** The relative expression of *FT* in the 12-day-old indicated genotypic seedlings at ZT13 under LD condition and at ZT10 under SD conditions. Expression levels of *FT* in **(a-c)** were determined by RT-qPCR, and values were normalized against *TUB2* expression. Data are means ± SE for 3 biological replicates × 3 technical replicates). * *p* < 0.05, ** *p* < 0.01, *** *p* < 0.001 by *t*-test (two-tailed). **d** m^6^A-IP-qPCR results showing the relative m^6^A levels of *FT* in Col-0 and *fiona1-1* plants. Data are means ± SE for 3 biological replicates × 2 technical replicates. 12-day-old seedlings at ZT13 were used in this analysis.
