# Supplementary material for: FIONA1 is an RNA N6-methyladenosine methyltransferase affecting Arabidopsis photomorphogenesis and flowering
Source: Genome Biol. 2022 Jan 31;23:40. doi: 10.1186/s13059-022-02612-2 (PMC8802475; doi:10.1186/s13059-022-02612-2)
Supplement: Supplementary file 2 — Additional file 2. Uncropped western blotting and gel analysis. [file 13059_2022_2612_MOESM2_ESM.docx]

Uncropped western blotting and gel analysis
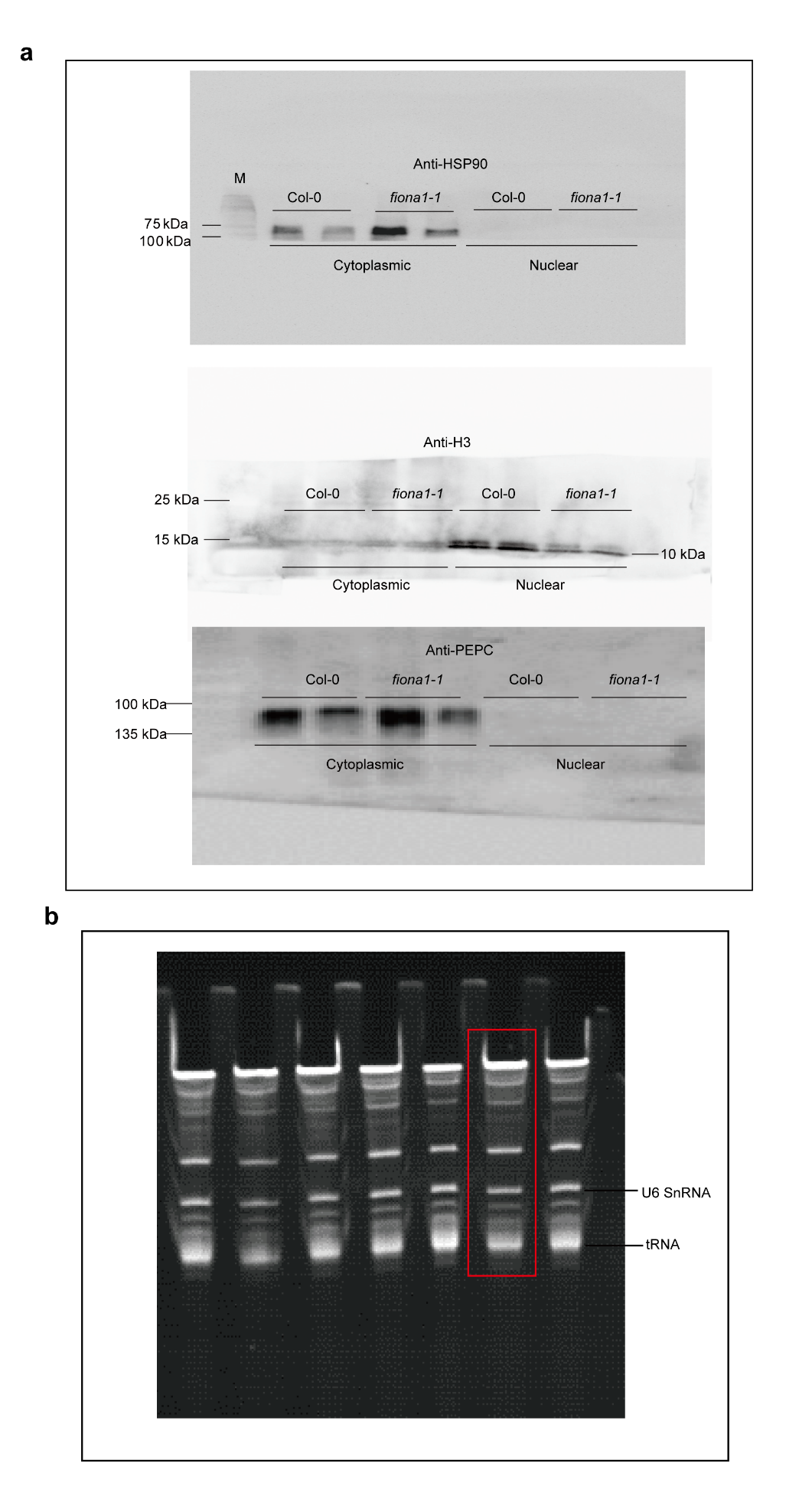


Uncropped western blotting and gel for Additional file 1: Fig. S3


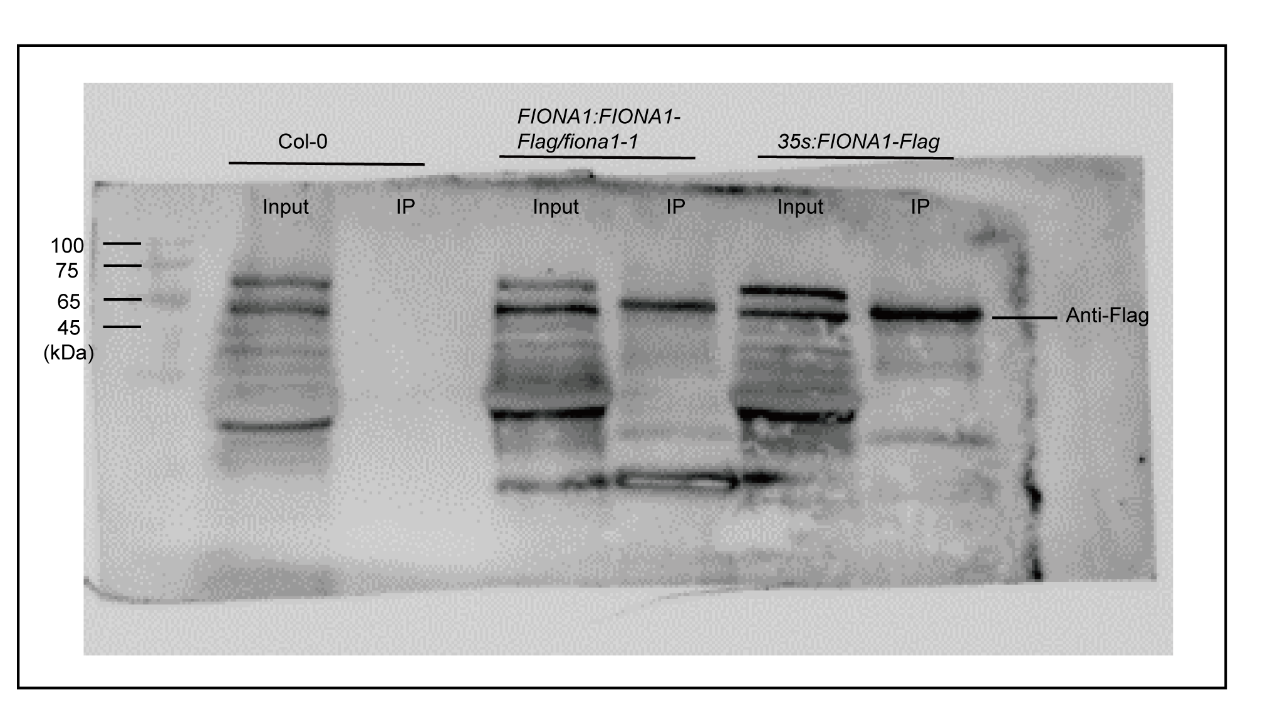


Uncropped western blotting for Additional file 1: S4d


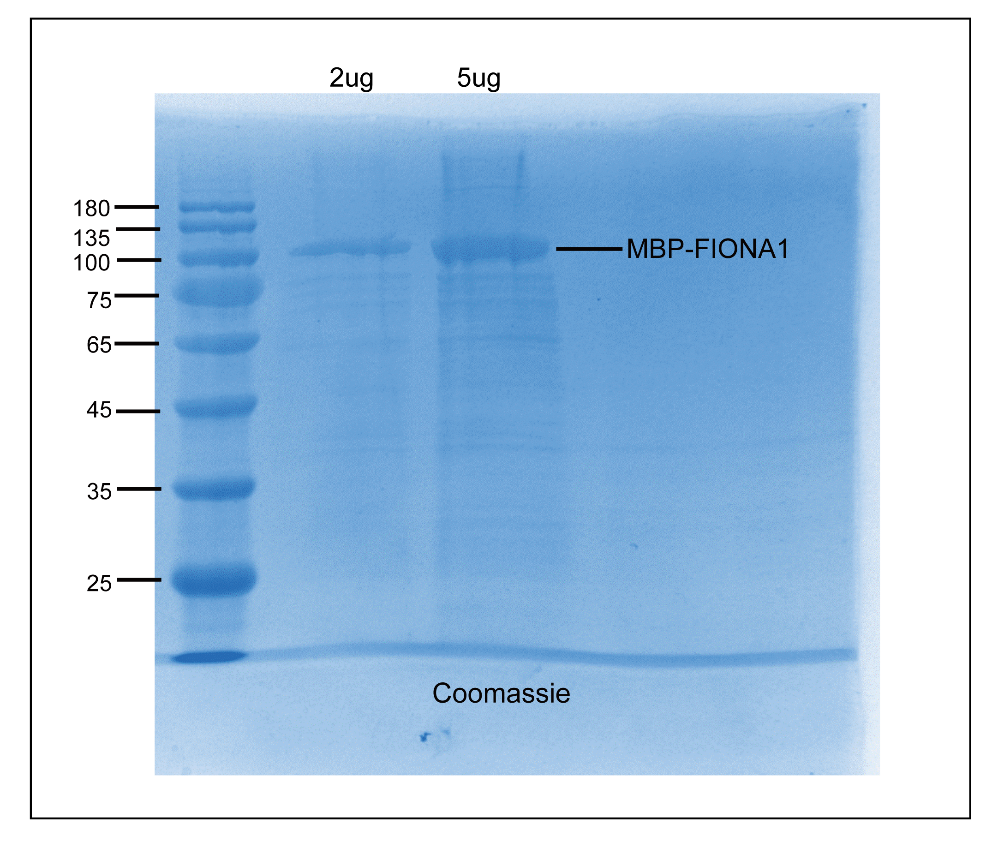


Uncropped SDS-PAGE gel for Additional file 1: S8.


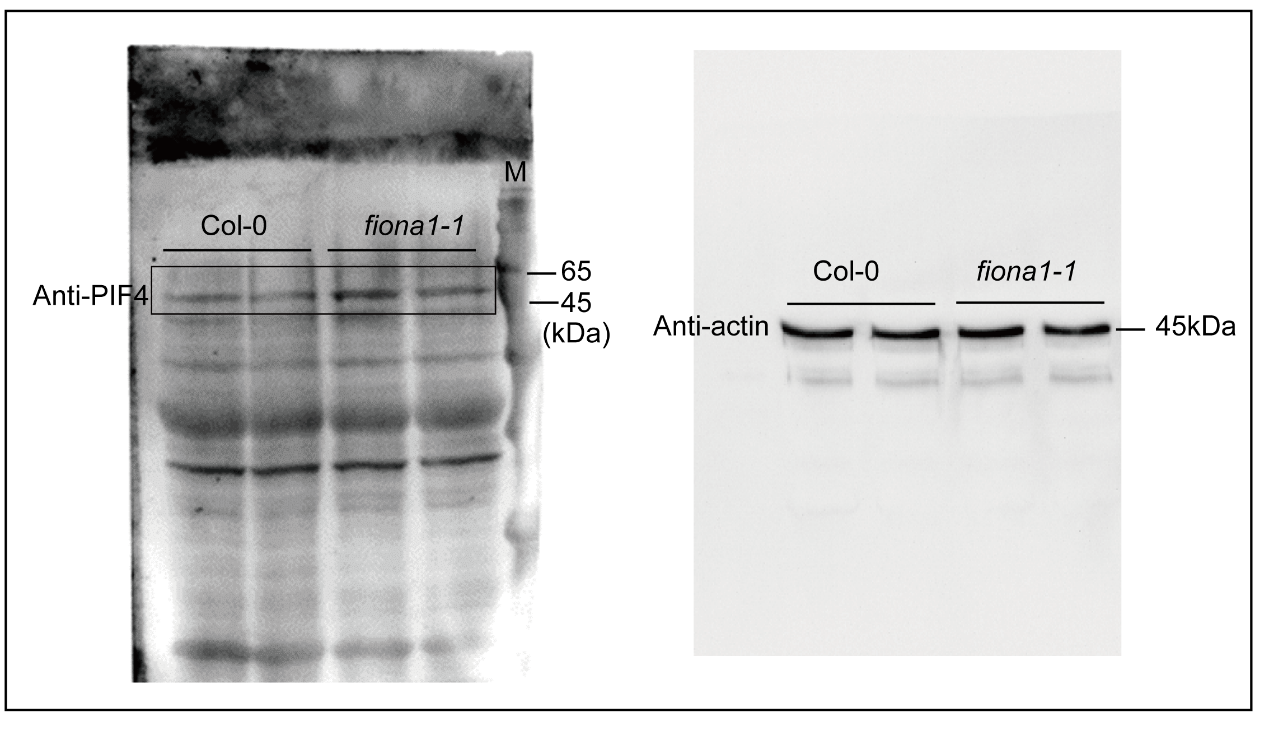


Uncropped western blotting for Additional file 1: S25b
