# Supplementary material for: FIONA1 is an RNA N6-methyladenosine methyltransferase affecting Arabidopsis photomorphogenesis and flowering
Source: Genome Biol. 2022 Jan 31;23:40. doi: 10.1186/s13059-022-02612-2 (PMC8802475; doi:10.1186/s13059-022-02612-2)
Supplement: Supplementary file 10 — Additional file 10. Review history. [file 13059_2022_2612_MOESM10_ESM.docx]

Review History

**First round of review**

**Reviewer 1**

**Are you able to assess all statistics in the manuscript, including the appropriateness of statistical tests used?**

Yes, and I have assessed the statistics in my report.

**Comments to author:**

This interesting manuscript reports on the role of an mRNA m6A methyltransferase FIONA1, the homolog of human METTL16, on Arabidopsis photomorphogenesis and flowering. The presented data are extensive and of high quality, and they point to a role for the FIONA1-mediated m6A post-transcriptional regulation for flowering and phytochrome signaling-dependent photomorphogenesis. The function of FIONA1 is distinct from human METTL16, which install m6A in mRNAs of SAM synthetases to regulate SAM level. The authors provide evidences that FIONA1 installs m6A in U6 snRNA and a small subset of poly(A) + RNA, including those involved in photomorphogenesis and floral transition. I have some specific comments as follows:

1. Page 2, line 35: It's better to present the specific transcripts related to phytochrome signaling and floral transition, i.e. PIF4, CRY2, CO, and FLC.

2. Page 2, line 38: change "Human" to "human".

3. Page 5, line 101: "U6 snRNA" should be described with more details.

4. Page 10, line 209-211: The conclusion seems a little absolute since the m6A methyltransferases or demethylases could be regulated at post-transcriptional levels, which do not affect their mRNA expression levels.

5. Page 11, line 236-238: The background about phytochrome A and phytochrome B should be described to make it clearly.

6. Page 13, line 279-282: How many hypermethylated m6A peaks were identified in the fiona-1 mutant compared to the Col-0? As FIONA acts as the m6A methyltransferase targeting poly(A)+ RNA as well as U6 snRNA, disruption of FIONA is expected to lead to significantly more hypomethylated m6A peaks than hypermethylated m6A peaks in gene transcripts.

7. Page 15, line 320-322: The sentence "mRNA modification m6A regulates alternative splicing in …" need to be modified. Add "of" after "removal".

8. Page 20, line 423-426: change "Arabidopsis" to "plant". Additionally, the recent literatures regarding MTA-mediated m6A modification on fruit ripening (Genome Biology, 2021, 22:168) and VIR-mediated m6A on salt stress (Plant Journal, 2021, in press. DOI: 10.1111/tpj.15270) could be updated here.

9. Fig 1 and 2: Whether there exist be significant differences in total m6A levels, hypocotyl length, and cell length between col-0 and FIONA1:FIONA1/fiona1-1? The statistical analysis is needed to support the conclusions that the intact FIONA1 can rescue the mutant phenotypes.

**Reviewer 2**

**Are you able to assess all statistics in the manuscript, including the appropriateness of statistical tests used?**

No, I do not feel adequately qualified to assess the statistics.

**Comments to author:**

In this study, the authors reported the m6A methylation activity FIONA1 in Arabidopsis and characterized FIONA1 as the homologous protein of METTL16 in mammals. FIONA1 has been reported to function in regulating phytochrome signaling and flowering previously. This study showed that the function of FIONA1 required its methylation activity. FIONA1 has different functions with METTL16 in mammals that it neither installs m6A in the mRNAs of Arabidopsis SAM synthetases nor affects their transcript expression levels and it does not have to require the UACm 6 AGAGAA motif to methylate mRNAs. FIONA1 installs m6A in U6 snRNA and a subset of poly(A)+ RNA including several phenotypic related transcripts. In general, the interesting point of the study was characterizing FIONA1 as the homologous protein of METTL16 in mammals. The disadvantage was the novelty of this study. The function of FIONA1 in Arabidopsis and METTL16 in mammals has been previously reported. Although the authors showed the relationship between FIONA1 and METTL16, they have not explained the differences between FIONA1 and METTL16 in mammals.

There are some specific suggestions as follows:

1, FIONA1 is required for the m6A modification of 2,572 genes, however, the hypomethylation of these genes did not affect their mRNA abundance and alternative splicing. What is the function of the m6A modification on RNAs mediated by FIONA1? In contrast, m6A in photomorphogenic and flowering related transcripts which are catalyzed by FIONA1 mediates their RNA abundance, and different genes have different regulatory mode. How to explain the inconsistency?

2, FIONA1 installs m6A modification in U6 snRNA in Arabidopsis which is similar with that of METTL16 in mammals. But the roles of FIONA1 during development are different with that of METTL16. What is the biological significance of the m6A modification in U6 snRNA mediated by METTL16 across species?

3, A comparison between the substrates of FIONA1, METTL3 and METTL16 will provide important information about their functional specificity and will explain why they are involved in different biological processes.

**Reviewer 3**

**Are you able to assess all statistics in the manuscript, including the appropriateness of statistical tests used?**

Yes, and I have assessed the statistics in my report.

**Comments to author:**

In this manuscript, Wang et al., found that FIONA1, a METTL16 homolog in Arabidopsis, installs m6A in U6 snRNA and a small subset of poly(A)+ RNA. fiona1 mutants generated by CRISPR/Cas9 affects hypocotyl elongation and flowering time. The authors further show that FIONA1 installs m6A in several transcripts related to reported phenotypes, such as PIF4, CRY2, CO and FLC, and affects their mRNA stability to regulate phytochrome signaling and flowering time. The results presented in this manuscript are primarily m6A-seq data, whereas the mechanisms regarding FIONA1 function and its regulation of downstream genes are preliminary. More pieces of solid experimental evidence need to be provided to support the major conclusions.

1. The authors performed two replicates of the m6A-seq to compare the difference in WT and fiona1 mutants. It is not clear whether these were biological or technique replicates. For either case, two replicates for each genotype were insufficient, especially for revealing the subtle differences between WT and fiona1 (i.e. fiona1 mutants only had an around 10% reduced content of m6A modifications in poly(A)+ RNA as shown in Figure 1D). More biological replicates (at least 3 biological replicates for each genotype) should be examined. In addition, it is also unclear how many biological replicates they performed RNA-seq.

2. The motif XGACC in which X stands for A/G/C the authors identified in the hypomethylated m6A peaks in fiona1 mutants resembles the known canonical m6A motif RRACH. In addition, the hypomethylated m6A peaks in fiona1 were predominantly enriched in stop codon and 3'UTR, which is similar to those have been observed for the known m6A writer complex mutants. This indicates that FIONA1 and other known m6A writer proteins may share some common targets. In view of the novelty and significance of results required for Genome Biology, it is necessary to compare the hypomethylated peaks in fiona1 and other mutants of known m6A writer proteins, and to address how FIONA1 acts redundantly with other m6A methyltransferases to mediate m6A modification on some common targets or specifically on different groups of targets.

3. In lines 355-358, the authors wrote "Based on our m6A-seq and RNA-seq results, we selected one photomorphogenic-related gene PIF4 and three flowering-related genes CRY2, CO and FLC for mechanism study". However, based on the m6A-seq and RNA-seq gene lists they provided in the Additional file 2, PIF4 is neither present in Table S4 (Hypomethylated m6A peaks identified in fiona1-1) nor in Table S5 (Differential expressed genes identified in fiona1 compared to WT by RNA-seq); CO is not present in Table S4, but appears with very high padj in Table S5; although CRY2 and FLC each has a hypomethylated m6A site, they are not in the differentially expressed gene list in Table S5. However, all changes in expression of these genes are significant by real-time PCR (Figure 5). Moreover, the authors should clearly state how they found out the hypomethylated sites in fiona1 mutants with FDR < 0.05 and fold change > 2. Based on the data they listed in Table S2 (WT) and Table S3 (fiona1), for example, the m6A enrichment fold of the FLC locus is 11.69473 in WT, whereas the fold is 10.04573 in fiona1. The difference is very small. In addition, the enrichment fold of 10.04573 of the FLC locus in fiona1 is also inconsistent with the data shown in Additional file Figure S19 where there is a complete loss of m6A at the FLC locus. All these inconsistencies do not build up the confidence on the results presented in this manuscript.

4. Only 1.4% m6A hypomethylated genes are overlapped with the differential expressed genes (lines 316-318), indicating that the regulation of RNA stability by FIONA1 as revealed by the authors is not a major mechanism. Have the authors checked other possibilities, such as translation or alternative polyadenylation?

5. The authors need to explain why FIONA1 has opposite effects on the transcript stability of CO, PIF4, CRY2 and FLC.

6. There are too many grammatic errors throughout the manuscript, English editing is required to improve the quality of the language.

**Authors Response**

**Point-by-point responses to the reviewers’ comments:**

Reviewer #1: This interesting manuscript reports on the role of an mRNA m6A methyltransferase FIONA1, the homolog of human METTL16, on Arabidopsis photomorphogenesis and flowering. The presented data are extensive and of high quality, and they point to a role for the FIONA1-mediated m6A post-transcriptional regulation for flowering and phytochrome signaling-dependent photomorphogenesis. The function of FIONA1 is distinct from human METTL16, which install m6A in mRNAs of SAM synthetases to regulate SAM level. The authors provide evidences that FIONA1 installs m6A in U6 snRNA and a small subset of poly(A) + RNA, including those involved in photomorphogenesis and floral transition.

*Response：We would like to thank the reviewers for their constructive and positive feedback. Based on the reviewers’ suggestions we have carried out substantially revised the manuscript. We believe that our manuscript has been improved by incorporating and implementing the reviewers’ comments. Details of the changes made during the revision are highlighted in red in the manuscript.*

I have some specific comments as follows:

1. Page 2, line 35: It's better to present the specific transcripts related to phytochrome signaling and floral transition, i.e. PIF4, CRY2, CO, and FLC.

*Response: Thanks for the suggestions. We have added the new sentence “We further showed that FIONA1 installs m6A in several phenotypic related transcripts, including PIF4, CRY2, CO, and FLC, thereby affecting downstream mRNA stability and regulating in phytochrome signaling and floral transition.” in our revised manuscript (See Line37-40).*

2. Page 2, line 38: change "Human" to "human".

Response#3: Considering the preciseness of the description and the latest research report, we have modified this sentence to “FIONA1 as Arabidopsis U6 m6A methyltransferase is distinct from mammalian METTL16 and worm METT-10” (See Line 41-42).

3. Page 5, line 101: "U6 snRNA" should be described with more details.

*Response: Thanks for the suggestions. We have added the sentence “The U6 snRNA is essential components of the spliceosome, contributing to splicing of nuclear pre-mRNAs and serving as ribozyme catalysts of two consecutive transesterifications to ligate two exons concomitant with removal of an intron [43]” in our manuscript (See Line 100-103).*

4. Page 10, line 209-211: The conclusion seems a little absolute since the m6A methyltransferases or demethylases could be regulated at post-transcriptional levels, which do not affect their mRNA expression levels.

*Response: We thank the reviewer for this comment. We have revised this sentence to: The results exclude the effects of MTA-MTB m6A methyltransferase complex or m6A demethylase on the m6A level of poly(A)+ RNA in fiona1-1 (See Line 214-216).*

5. Page 11, line 236-238: The background about phytochrome A and phytochrome B should be described to make it clearly.

*Response: Thanks for the suggestion. We added the description “In higher plants, phytochrome A and phytochrome B are the most abundant members of the photoreceptors family and their deficiencies are most evident under continuous Rc and FRc, respectively. Although phyA and phyB activities occur under different light conditions, the end-point responses (e.g., hypocotyl growth, cotyledon unfolding, flowering, etc.) controlled by which are largely the same [53].” in our manuscript (See Line 263-268).*

6. Page 13, line 279-282: How many hypermethylated m6A peaks were identified in the fiona-1 mutant compared to the Col-0? As FIONA acts as the m6A methyltransferase targeting poly(A)+ RNA as well as U6 snRNA, disruption of FIONA is expected to lead to significantly more hypomethylated m6A peaks than hypermethylated m6A peaks in gene transcripts.

*Response: In the revision we performed new m6A-seq in three biological replicates of fiona1-1 and WT plants. We identified 3,031 hypermethylated m6A peaks and 1,137 hypomethylated m6A peaks in fiona1-1 compared with Col-0 (Fig. 5a, b; Additional file 2: Table S4). Considering 10,840 m6A confident peaks identified in poly(A)+ RNA of Col-0 (Additional file 1: Fig. S14a), the fraction of the potential FIONA-methylated m6A sites (1,137 hypomethylated m6A peaks) was around 10% of total m6A sites in Col-0 poly(A)+ RNA, in consistent with the 10~15% of m6A level decreased in the fiona1-1 mutant plants. The m6A modification is regulated by complicated regulation pattern with lots of m6A regulator. Our findings suggest that FIONA1 is not the main m6A methyltransferase in plants, and only responsible for 10%~15% m6A modification, other unknown reason may be responsible for those hypermethylated m6A peaks.*

7. Page 15, line 320-322: The sentence "mRNA modification m6A regulates alternative splicing in …" need to be modified. Add "of" after "removal".

*Response: We added the word to the sentence “mRNA modification m6A regulates alternative splicing in mammals and mammalian METTL16 affects the splicing of MAT2A mRNA for intron retention [12, 13, 32, 58]. It was predicated that removal of m6A modification from U6 snRNA also could affect splicing [59, 60].” (See Line 355- 358).*

8. Page 20, line 423-426: change "Arabidopsis" to "plant". Additionally, the recent literatures regarding MTA-mediated m6A modification on fruit ripening (Genome Biology, 2021, 22:168) and VIR-mediated m6A on salt stress (Plant Journal, 2021, in press. DOI: 10.1111/tpj.15270) could be updated here. *Response: We have modified this word and added the sentence ‘Characterization of plant mRNA m6A writer complex, erasers, and readers revealed that mRNA modification m6A regulates developmental timing, leaf morphogenesis, flowering transition, trichome morphology, leaf growth, nitrate signaling, viral response, fruit ripening, ABA response, and salt tolerance [22-30, 69, 70].’ in our revised manuscript. (See Line 503-506).*

9. Fig 1 and 2: Whether there exist be significant differences in total m6A levels, hypocotyl length, and cell length between col-0 and FIONA1:FIONA1/fiona1-1? The statistical analysis is needed to support the conclusions that the intact FIONA1 can rescue the mutant phenotypes.

*Response: Thank for the suggestions. We have compared the significant differences in total m6A levels, hypocotyl length, and cell length between Col-0 and FIONA1:FIONA1/fiona1-1, and there were no significant differences between them. Please see the revised Fig.1and Fig.3.*

Reviewer#2:

In this study, the authors reported the m6A methylation activity FIONA1 in Arabidopsis and characterized FIONA1 as the homologous protein of METTL16 in mammals. FIONA1 has been reported to function in regulating phytochrome signaling and flowering previously. This study showed that the function of FIONA1 required its methylation activity. FIONA1 has different functions with METTL16 in mammals that it neither installs m6A in the mRNAs of Arabidopsis SAM synthetases nor affects their transcript expression levels and it does not have to require the UACm 6 AGAGAA motif to methylate mRNAs. FIONA1 installs m6A in U6 snRNA and a subset of poly(A)+ RNA including several phenotypic related transcripts. In general, the interesting point of the study was characterizing FIONA1 as the homologous protein of METTL16 in mammals. The disadvantage was the novelty of this study. The function of FIONA1 in Arabidopsis and METTL16 in mammals has been previously reported. Although the authors showed the relationship between FIONA1 and METTL16, they have not explained the differences between FIONA1 and METTL16 in mammals.

*Response：We thank this referee for the thorough review. Mammalian METTL16 and worm METT-10 as U6 m6A methyltransferase regulates SAM homeostasis through installation of m6A in SAM synthetase pre-mRNA with two different mechanisms (Cell. 2017, 169, 824-835; Cell. 2021, 184, 3125-3142). In the revision, we performed new experiments to demonstrate that FIONA1 as Arabidopsis U6 m6A methyltransferase is distinct from mammalian METTL16 and worm METT-10: 1) FIONA1 does not install m6A in pre-mRNAs of SAM synthetases to regulate intercellular SAM level under normal and high SAM conditions; 2) FIONA1 can install m6A on plant mRNA m6A motifs in vitro and in vivo; 3) The methylation activity of FIONA1 does not depend on stem-loop structure.*

There are some specific suggestions as follows: 1, FIONA1 is required for the m6A modification of 2,572 genes, however, the hypomethylation of these genes did not affect their mRNA abundance and alternative splicing. What is the function of the m6A modification on RNAs mediated by FIONA1? In contrast, m6A in photomorphogenic and flowering related transcripts which are catalyzed by FIONA1 mediates their RNA abundance, and different genes have different regulatory mode. How to explain the inconsistency?

*Response: FIONA1 as a U6 m6A methyltransferase can install m6A on a small subset of mRNA. We reperformed m6A-seq in three biological replicates of fiona1-1 and WT plants and identified 1,137 hypomethylated m6A peaks corresponding 1,128 transcripts/genes in fiona1-1 (Fig. 5a, b; Additional file 2: Table S4), which could potential FIONA1 methylation targets. m6A readers recognizes m6A to serve m6A functions in mRNA processing and metabolism, including promoting mRNA degradation, mRNA stabilization, and mRNA translation, etc. Thus, the function of FIONA1 is to install m6A, however, the m6A role in RNA processing depends on m6A reader, no m6A writer. FIONA1-mediated m6A formation in different genes could be recognized by m6A readers to affect mRNA degradation, stabilization, and translation, etc. In the manuscript, we have identified four FIONA1 targets CO, CRY2, FLC, and PIF4. Disruption of FIONA1 reduces m6A on PIF4, CO, CRY2, and FLC transcripts, leading upregulated expression levels of PIF4, CO, and CRY2 and downregulated FLC expression. Clearly, m6A on PIF4, CO, and CRY2 promotes mRNA degradation while m6A on FLC promotes mRNA stabilization. These two types of m6A functions are mediated by different m6A readers.*

*Note: The raw sequence data of RNA-seq, m6A-seq this paper have been deposited in the Genome Sequence Archive in the National Genomics Data Center (NGDC), Beijing Institute of Genomics, Chinese Academy of Sciences, under accession number (CRA004052). (Shared URL: https://ngdc.cncb.ac.cn/gsa/s/h0BnTqTa).*

2, FIONA1 installs m6A modification in U6 snRNA in Arabidopsis which is similar with that of METTL16 in mammals. But the roles of FIONA1 during development are different with that of METTL16. What is the biological significance of the m6A modification in U6 snRNA mediated by METTL16 across species?

*Response: Mammalian METTL16 and worm METT-10 were discovered as U6 m6A methyltransferase, their functions on U6 snRNA still remain unclear. According to the published literature, the functional roles of mammalian METTL16 and worm METT-10 in developments (or phenotypes) were from METTL16/METT-10-mediated m6A methylation on SAM synthetase, which affects splicing and SAM homeostasis (Cell. 2017, 169, 824-835; Cell. 2021, 184, 3125-3142).*

*In the revision, we performed new experiments to demonstrate that FIONA1 as Arabidopsis U6 m6A methyltransferase is distinct from mammalian METTL16 and worm METT-10: 1) FIONA1 does not install m6A in pre-mRNAs of SAM synthetases to regulate intercellular SAM level under normal and high SAM conditions (Additional file1: Fig. S19, 20); 2) FIONA1 can install m6A on plant mRNA m6A motifs in vitro and in vivo (Fig.2; Fig.5e; Fig.6c). 3) The methylation activity of FIONA1 does not depend on stem-loop structure (Fig.2).*

*The function of m6A in U6 snRNA was predicted to affect splicing [32, 60]. Although mammalian METTL16 and worm METT-10 were discovered as U6 m6A methyltransferase, their regulatory functions on U6 still remain unclear. Here we preliminarily investigated whether m6A in U6 affects splicing. Our analysis showed that disruption of FIONA1 only affects 43 genes’ alternative splicing events and none of them are related with the observed phenotypes (Additional file 2: Table S6), suggesting removal of U6 m6A does not globally affect splicing. Our results rule out the possible effects of loss of U6 m6A methylation on the phenotypes in fiona1-1 mutant. It needs further explore the m6A function on U6 snRNA. (See Line554-562).*

3, A comparison between the substrates of FIONA1, METTL3 and METTL16 will provide important information about their functional specificity and will explain why they are involved in different biological processes.

*Response: We thank referee for the suggestion. Firstly, we performed new experiments compared the functional differences between FIONA1 and METTL16 (or METT-10). In the revision, we found several unique features of FIONA1, which are distinct from mammalian METTL16 and worm METT-10. 1) FIONA1 neither installs m6A in the transcripts of Arabidopsis SAM synthetases (MAT1-4) nor affect their transcript expression levels under normal or high SAM conditions (Additional file1: Fig. S19, 20). 2) FIONA1 can install m6A on plant mRNA m6A motifs in vitro and in vivo. We performed in vitro methylation experiments to reveal that FIONA1 not only methylates U6 m6A motif, but also assembles m6A for RNA containing GGAAC or UGUA motif (Fig.2). The single-based m6A site validation by SELECT method in fiona1-1 and WT plants further confirmed that FIONA1 can install m6A in UGUAU and GGACC sequences of PIF4 mRNA and UGUAG sequence of CRY2 mRNA in vivo (Fig. 6c). 3) The methylation activity of FIONA1 does not depend on stem-loop structure. We found FIONA1 exhibits higher activity towards U6 m6A motif in a linear RNA over in a stem-loop structured RNA (Fig.2).*

*Secondly, we compared FIONA1 potential methylation targets and non-FIONA1 targets with the published FIP37 potential methylation targets and revealed that FIONA1 and FIP37 methylate different m6A sites and FIP37 targets are largely overlapped with nonFIONA targets (Additional file 1: Fig. S17). The fraction of the potential FIONAmethylated m6A sites (1,137 hypomethylated m6A peaks) was around 10% of total m6A sites in Col-0 poly(A)+ RNA, in consistent with the 10~15% of m6A level decreased in the fiona1-1 mutant plants. The m6A writer complex comprising the key subunits MTA, MTB, and FIP37 is responsible for the majority of poly(A)+ RNA m6A methylation. More than 80% of m6A level in poly(A)+ RNA was reduced in the fip37-4 LEC1:FIP37 plants. Thus, our results suggest that FIONA1 methylates no more than 15% of m6A sites in poly(A)+ RNA; the rest m6A sites in poly(A)+ RNA are installed by the m6A writer complex containing MTA/MTB/FIP37.*

Reviewer#3:

In this manuscript, Wang et al., found that FIONA1, a METTL16 homolog in Arabidopsis, installs m6A in U6 snRNA and a small subset of poly(A)+ RNA. fiona1 mutants generated by CRISPR/Cas9 affects hypocotyl elongation and flowering time. The authors further show that FIONA1 installs m6A in several transcripts related to reported phenotypes, such as PIF4, CRY2, CO and FLC, and affects their mRNA stability to regulate phytochrome signaling and flowering time. The results presented in this manuscript are primarily m6A-seq data, whereas the mechanisms regarding FIONA1 function and its regulation of downstream genes are preliminary. More pieces of solid experimental evidence need to be provided to support the major conclusions. Response#15：We thank this referee for the thorough review. In the revision, we have performed new experiments to support the conclusions: 1) FIONA1 as Arabidopsis U6 m6A methyltransferase is distinct from its homologs, Mammalian METTL16 and worm METT-10. We found that FIONA1 does not install m6A in pre-mRNAs of SAM synthetases to regulate intercellular SAM level under normal and high SAM conditions; it can install m6A on plant mRNA m6A motifs in vitro and in vivo and its methylation activity does not depend on stem-loop structure. 2) Our results demonstrate that FIONA1-mediated m6A post-transcriptional regulation is an autonomous regulator for flowering and phytochrome signaling-dependent photomorphogenesis. 3) Our results suggest that FIONA1 methylates no more than 15% of m6A sites in poly(A)+ RNA; the rest m6A sites in poly(A)+ RNAs are installed by the m6A writer complex containing MTA/MTB/FIP37. They methylate different m6A sites in poly(A)+ RNA.

1. The authors performed two replicates of the m6A-seq to compare the difference in WT and fiona1 mutants. It is not clear whether these were biological or technique replicates. For either case, two replicates for each genotype were insufficient, especially for revealing the subtle differences between WT and fiona1 (i.e. fiona1 mutants only had an around 10% reduced content of m6A modifications in poly(A)+ RNA as shown in Figure 1D). More biological replicates (at least 3 biological replicates for each genotype) should be examined. In addition, it is also unclear how many biological replicates they performed RNA-seq.

*Response: In the revision, we reperformed m6A-seq in three biological replicates of fiona1-1 and WT plants and identified 1,137 hypomethylated m6A peaks corresponding 1,128 transcripts/genes in fiona1-1 (Fig. 5a, b; Additional file 2: Table S4), which could be potential FIONA1 methylation targets. Considering 10,840 m6A confident peaks identified in poly(A)+ RNA of Col-0 (Additional file 1: Fig. S14a), the fraction of the potential FIONA-methylated m6A sites (1,137 hypomethylated m6A peaks) was around 10% of total m6A sites in Col-0 poly(A)+ RNA, in consistent with the 10~15% of m6A level decreased in the fiona1-1 mutant plants. We performed RNA-seq in two biological replicates. The raw sequence data of RNA-seq, m6A-seq this paper have been deposited in the Genome Sequence Archive in the National Genomics Data Center (NGDC), Beijing Institute of Genomics, Chinese Academy of Sciences, under accession number (CRA004052). (Shared URL:* [*https://ngdc.cncb.ac.cn/gsa/s/h0BnTqTa*](https://ngdc.cncb.ac.cn/gsa/s/h0BnTqTa)*).*

2. The motif XGACC in which X stands for A/G/C the authors identified in the hypomethylated m6A peaks in fiona1 mutants resembles the known canonical m6A motif RRACH. In addition, the hypomethylated m6A peaks in fiona1 were predominantly enriched in stop codon and 3'UTR, which is similar to those have been observed for the known m6A writer complex mutants. This indicates that FIONA1 and other known m6A writer proteins may share some common targets. In view of the novelty and significance of results required for Genome Biology, it is necessary to compare the hypomethylated peaks in fiona1 and other mutants of known m6A writer proteins, and to address how FIONA1 acts redundantly with other m6A methyltransferases to mediate m6A modification on some common targets or specifically on different groups of targets.

*Response: Thanks for the suggestion. In the revision, we compared FIONA1 potential methylation targets and non-FIONA1 targets with the published FIP37 potential methylation targets (Dev Cell. 2016, 38, 186-200) and revealed that FIONA1 and FIP37 methylate different m6A sites and FIP37 targets are largely overlapped with non-FIONA targets (Additional file 1: Fig. S17). More than 80% of m6A level in poly(A)+ RNA was reduced in the fip37-4 LEC1:FIP37 plants while only 10~15% of m6A level was decreased in the fiona1-1 mutant plants. Thus, our results suggest that FIONA1 methylates no more than 15% of m6A sites in poly(A)+ RNA; the rest m6A sites in poly(A)+ RNA are installed by the m6A writer complex containing MTA/MTB/FIP37.*

3. In lines 355-358, the authors wrote "Based on our m6A-seq and RNA-seq results, we selected one photomorphogenic-related gene PIF4 and three flowering-related genes CRY2, CO and FLC for mechanism study". However, based on the m6A-seq and RNA-seq gene lists they provided in the Additional file 2, PIF4 is neither present in Table S4 (Hypomethylated m6A peaks identified in fiona1-1) nor in Table S5 (Differential expressed genes identified in fiona1 compared to WT by RNA-seq); CO is not present in Table S4, but appears with very high padj in Table S5; although CRY2 and FLC each has a hypomethylated m6A site, they are not in the differentially expressed gene list in Table S5. However, all changes in expression of these genes are significant by real-time PCR (Figure 5).

*Response: In the revision, we reperformed m6A-seq in three biological replicates and RNA-seq in two biological replicates. The m6A-seq and RNA-seq results showed that m6A at 3' UTR of CRY2 (CRYPTOCHROMES 2) and FLC (FLOWERING LOCUS C) transcripts were significantly reduced in fiona1-1 (Additional file 1: Fig. S21; Additional file 2: Table S4) and their mRNA expression levels were also significantly changed (Additional file 2: Table S5). CO (CONSTANS) is a key regulator of the photoperiodic flowering pathway [62]. The RNA-seq showed that CO mRNA was significantly upregulated in fiona1-1, consistent with previous report [47]. We did not identify m6A peak on CO transcript in the m6A-seq results, which might be the low expression level of CO at Zeitgeber time (ZT) 13 of LD condition. Thus, we chose three flowering-related genes CRY2, FLC, and CO for further validation. PIF4 (PHYTOCHROME INTERACTING FACTOR4) directly interacts with light-activated phytochromes and is a positive regulator in cell elongation [63]. The hypocotyl elongation of pif4 mutants are specifically defective in responsiveness to red light, the double mutant of PIF4 and its close homolog PIF5 (pif4pif5) show hypersensitivity to far-red light, suggesting that they redundantly control the far-red light responses [64]. In consistent with phenotype of hypocotyl cell elongation selectively under Rc) and FRc in fiona1 mutants, RNA-seq revealed upregulation of PIF4 expression level in fiona1-1 (Additional file 2: Table S5). Among three biological replicate m6A-seq, we observed the significant m6A decreases in two biological replicates of fiona1-1 samples (Additional file 1: Fig. S21). Therefore, we selected PIF4 for further validation. We have stated the reason for picking these genes in the revised manuscript (See Line 419- 438).*

*Additionally, we performed m6A-IP-qPCR and SELECT methods to validate these four genes are FIONA1 targets in vivo. SELECT method is a qPCR method for quantitative detection of m6A locus and fraction in mRNA at single-base resolution. The method has been widely used in many published literatures for determining m6A sites and writer/eraser targeted m6A sites (Nature. 2021, 591, 317-321; Nat Chem Biol. 2019, 15, 865-871). SELECT validated that two m6A sites in UGUm6A1943U and GGm6A1845CC sequences in PIF4 and one m6A site in UGUm6A2294G sequence in CRY2 were methylated by FIONA1 in vivo. In the revision, we performed in vitro methylation experiments to reveal that FIONA1 not only methylates U6 m6A motif, but also installs m6A on plant mRNA m6A motifs like GGAAC or UGUA motif. Thus, we confirm that FIONA1 can install m6A on non-U6 m6A motifs in phenotype-related transcripts CO, CRY2, FLC, and PIF4.*

Moreover, the authors should clearly state how they found out the hypomethylated sites in fiona1 mutants with FDR < 0.05 and fold change > 2. Based on the data they listed in Table S2 (WT) and Table S3 (fiona1), for example, the m6A enrichment fold of the FLC locus is 11.69473 in WT, whereas the fold is 10.04573 in fiona1. The difference is very small. In addition, the enrichment fold of 10.04573 of the FLC locus in fiona1 is also inconsistent with the data shown in Additional file Figure S19 where there is a complete loss of m6A at the FLC locus. All these inconsistencies do not build up the confidence on the results presented in this manuscript.

*Response: We cannot directly use the ratio of m6A enrichment in fiona1 vs m6A enrichment in Col-0 to identify the differential m6A peaks in fiona1, because the input RNA expression levels between these two samples are different. Thus, we used the exomePeak software to identified FIONA1-dependent m6A peaks. The exomePeak software is developed specifically for identification of m6A differential peaks between two treated samples, and has been widely used in many literatures (Bioinformatics. 2013, 29, 1565-1567; Nat Cell Biol. 2018, 20, 1074-1083; Cell. 2018,172, 90-105). ExomePeak consists of two major steps: determining and statistical modeling putative differentially m6A sites and significance test for the inferred regions. In the first step, exomePeak determines the putative DMSs to be tested for differential methylation in treated vs. untreated conditions. DMSs are determined based on the corresponding methylation sites in untreated or treated samples predicted by developed peak calling software HEPeak. Then, exomePeak models the reads variation of a putative DMS by a beta-binominal distribution. In the second step, MeTDiff computes the statistical significance using a likelihood ratio test, based on which the DMSs between two conditions are predicted.*

4. Only 1.4% m6A hypomethylated genes are overlapped with the differential expressed genes (lines 316-318), indicating that the regulation of RNA stability by FIONA1 as revealed by the authors is not a major mechanism. Have the authors checked other possibility, such as translation or alternative polyadenylation?

*Response: FIONA1, as an m6A writer, installs m6A in specific phenotype related transcripts, and different readers are recruited to recognized those m6A to regulate different mRNA processing and metabolism including promoting mRNA degradation, mRNA stabilization, and mRNA translation, etc. Thus, the function of FIONA1 is to install m6A, however, the m6A role in RNA processing depends on m6A reader, no m6A writer. FIONA1-mediated m6A formation in different genes could be recognized by m6A readers to affect mRNA degradation, stabilization, and translation, etc. Here, our purpose is to identify FIONA1 as U6 m6A writer that can install m6A on specific phenotype-related transcripts, leading to the phenotypes.*

5. The authors need to explain why FIONA1 has opposite effects on the transcript stability of CO, PIF4, CRY2 and FLC.

*Response: The m6A readers recognizes m6A to serve m6A functions in mRNA processing and metabolism, including promoting mRNA degradation, mRNA stabilization, and mRNA translation, etc. Thus, the function of FIONA1 is to install m6A, however, the m6A role in RNA processing depends on m6A reader, no m6A writer. FIONA1-mediated m6A formation in different genes could be recognized by different m6A readers to affect mRNA degradation, stabilization, or translation, etc. In the manuscript, we have identified four FIONA1 targets CO, CRY2, FLC, and PIF4. Disruption of FIONA1 reduces m6A on PIF4, CO, CRY2, and FLC transcripts, leading upregulated expression levels of PIF4, CO, and CRY2 and downregulated FLC expression. Clearly, m6A on PIF4, CO, and CRY2 promotes mRNA degradation while m6A on FLC promotes mRNA stabilization. These two types of m6A functions are mediated by different m6A readers.*

6. There are too many grammatic errors throughout the manuscript, English editing is required to improve the quality of the language.

*Response: We have revised the manuscript carefully.*

**Second round of review**

**Reviewer 1**

The authors have addressed my questions and improved the manuscript substantially.

**Reviewer 2**

Thank you for the author's responces. The arthors have addressed most of our concerns and questions. I have no other suggestions.
